# Global Solutions of the One-Dimensional Compressible Euler Equations with Nonlocal Interactions via the Inviscid Limit

**DOI:** 10.1007/s00205-025-02097-w

**Published:** 2025-05-24

**Authors:** José A. Carrillo, Gui-Qiang G. Chen, Difan Yuan, Ewelina Zatorska

**Affiliations:** 1 Mathematical Institute, University of Oxford, Oxford, OX2 6GG UK; 2 School of Mathematical Sciences, Beijing Normal University and Laboratory of Mathematics and Complex Systems, Beijing, China; 3 Mathematics Institute, University of Warwick, Coventry, CV4 7AL UK

## Abstract

We are concerned with the global existence of finite-energy entropy solutions of the one-dimensional compressible Euler equations with (possibly) damping, alignment forces, and the nonlocal interactions of Newtonian repulsion and quadratic confinement. Both the polytropic gas law and the general gas law are analyzed. This is achieved by constructing a sequence of solutions of the one-dimensional compressible Navier–Stokes-type equations with density-dependent viscosity on expanding intervals with the stress-free boundary condition and then taking the vanishing viscosity limit. The main difficulties in this paper arise from the appearance of the nonlocal terms. In particular, some uniform higher moment estimates of the solutions for the compressible Navier–Stokes equations on the expanding intervals with stress-free boundary condition are obtained by careful design of the approximate initial data.

## Introduction

Hydrodynamic models of collective behavior provide a comprehensive framework for characterizing the behavior of vast assemblies of interacting individuals. In the most interesting cases, these models can only be formally derived from the particle-type systems capturing precise interactions between the individuals. In the cases where full mathematical rigor is attainable, it is usually established through the mean-field limit techniques or the BBGKY hierarchies; see [5, 7, 28] and the classical references therein on the matter. In this paper, we are interested in a specific one-dimensional (1-D) example of such models, which captures local repulsion, and nonlocal attraction and repulsion forces, as well as nonlocal alignment. This model is described by the compressible Euler equations (CEEs) with the corresponding interaction forces:

1.1 $$\begin{aligned} {\left\{ \begin{array}{ll} \rho _t+m_x=0,\\ m_t+(\frac{m^2}{\rho }+P(\rho ))_x =\lambda m-\rho \partial _xW*\rho +\displaystyle \int _{\mathbb {R}}\varpi (x-y)\big (\rho (x) m(y)-\rho (y) m(x)\big )\,\textrm{d}y, \end{array}\right. } \end{aligned}$$

for $t>0$ and $x\in \mathbb {R}$, where $\rho \geqq 0$ is the density, *m* is the momentum, and *P* is the pressure.

The nonlocal attraction-repulsion interaction force is described by potential *W* of the form

$$\begin{aligned} W(x)=-|x|+\frac{x^2}{2}. \end{aligned}$$

The long-range attraction between the individuals is captured by the quadratic confinement part $\frac{1}{2}x^2,$ while the short-range repulsion is described by the Newtonian part of the potential $-|x|.$

The consensus in velocities (the alignment) among individuals is described by the weight $\varpi$ that satisfies

$$ \varpi \in L^{\infty }(\mathbb {R}),\quad \varpi (x)=\varpi (-x), \quad \varpi \geqq 0. $$

Finally, the linear term $\lambda m$ in our system stands for the damping, if the coefficient $\lambda$ is non-positive.

### The equation of state

We consider a general pressure law $P(\rho )$ satisfying hypotheses ($\mathcal {H}$) formulated below:

- ($\mathcal {H}.1$) The pressure function $P(\rho )\in C^1([0,\infty ))\cap C^4((0,\infty ))$ satisfies the hyperbolic and genuinely nonlinear conditions
  1.2 $$\begin{aligned} P'(\rho )>0,\quad 2P'(\rho )+\rho P''(\rho )>0\qquad \,\, \text {for }\rho >0. \end{aligned}$$
- ($\mathcal {H}.2$) There exist constants $\rho _{*}>0$, $\gamma _1\in (1,3)$, and $\kappa _1>0$ such that
  1.3 $$\begin{aligned} P(\rho )=\kappa _{1} \rho ^{\gamma _1}\big (1+\mathcal {P}_1(\rho )\big ) \qquad \text{ for } \rho \in [0, \rho _{*}) \end{aligned}$$
  for some function $\mathcal {P}_1(\rho )\in C^4((0,\infty ))$ satisfying
  $$|\mathcal {P}_1^{(j)}(\rho )|\leqq C_{*}\rho ^{\gamma _1-1-j} \qquad \text{ for } \rho \in (0,\rho _{*}) \,\, \text{ and } j=0,\ldots ,4,$$
  where $C_{*}>0$ depends only on $\rho _{*}$.
- ($\mathcal {H}.3$) There exist constants $\rho ^{*}> \rho _{*}>0$, $\gamma _2\in (1,\gamma _{1}]$, and $\kappa _{2}>0$ such that
  1.4 $$\begin{aligned} P(\rho )=\kappa _2\rho ^{\gamma _2}\big (1+\mathcal {P}_2(\rho )\big ) \qquad \text{ for } \rho \in [\rho ^{*},\infty ) \end{aligned}$$
  for some function $\mathcal {P}_2(\rho )\in C^{4}((0,\infty ))$ satisfying
  $$\begin{aligned} |\mathcal {P}_{2}^{(j)}(\rho )|\leqq C^{*}\rho ^{-\epsilon -j}\qquad \text{ for } \rho \in [\rho ^{*},\infty ),\ \epsilon >0,\ \text{ and } j=0,\ldots ,4, \end{aligned}$$
  where $C^{*}>0$ depends only on $\rho ^{*}$.

Without loss of generality, by scaling, $\kappa _1$ may be chosen to be equal to $\kappa _1=\frac{(\gamma _1-1)^2}{4\gamma _1}$.

A special example of such an equation of state is the pressure-density relation for the *polytropic gases*

1.5 $$\begin{aligned} P(\rho )=\kappa \rho ^{\gamma }\qquad \text { for }\gamma \in (1,\infty ), \end{aligned}$$

where $\gamma$ is the adiabatic exponent. In this case, we can choose $\kappa =\frac{(\gamma -1)^2}{4\gamma }$.

### Initial data

We consider the Cauchy problem for (1.1) with the initial data

1.6 $$\begin{aligned} (\rho , m)|_{t=0}=(\rho _0, m_0)(x) \end{aligned}$$

such that $\rho _0\in L^1_+(\mathbb {R})$ and $\frac{m_0}{\sqrt{\rho _0}}\in L^2(\mathbb {R})$ with finite initial total mass and initial second moment

1.7 $$\begin{aligned} M:=\int _{\mathbb {R}} \rho _0(x)\,\textrm{d}x<\infty , \qquad M_2:=\int _{\mathbb {R}}x^2\rho _0(x)\,\textrm{d}x<\infty . \end{aligned}$$

We further assume that the initial data are of finite energy:

1.8 $$\begin{aligned} \mathcal {E}_0:= \int _{\mathbb {R}}\Big (\frac{1}{2} \Big |\frac{m_0}{\sqrt{\rho _0}}\Big |^2 + \rho _0e(\rho _0)+\frac{1}{2}\rho _0 W*\rho _0\Big )(x) \,\text {d}x<\infty , \end{aligned}$$

where $e(\rho )$ is the internal energy

$$\begin{aligned} e(\rho )=\int _0^\rho \frac{P(\sigma )}{\sigma ^2} \,\textrm{d}\sigma . \end{aligned}$$

In particular, for the polytropic case (1.5),

$$\begin{aligned} e(\rho ):=\frac{\kappa }{\gamma -1}\rho ^{\gamma -1}. \end{aligned}$$

### Main objectives of the paper

The goal of the present paper is to establish the global-in-time existence of finite-energy entropy solutions of system (1.1) without restriction on the size of initial data. Our method of choice is the vanishing viscosity limit for the strong solutions of the compressible Navier–Stokes equations (CNSEs). More precisely, we construct the solution by means of a sequence of approximate problems

1.9 $$\begin{aligned} {\left\{ \begin{array}{ll} \displaystyle \rho ^{\varepsilon }_t+ (\rho ^{\varepsilon }u^{\varepsilon })_x=0,\\ \displaystyle (\rho ^{\varepsilon }u^{\varepsilon })_t+\big (\rho ^{\varepsilon }(u^{\varepsilon })^2+P(\rho ^{\varepsilon })\big )_x= \varepsilon (\mu (\rho ^{\varepsilon })u^{\varepsilon }_x)_x+\lambda \rho ^{\varepsilon }u^{\varepsilon }-\rho ^{\varepsilon }\partial _xW*\rho ^{\varepsilon }\\ \qquad \qquad \qquad \qquad \qquad \qquad \qquad \qquad \qquad \displaystyle +\rho ^{\varepsilon }\int _{\mathbb {R}}\varpi (x-y)(u^{\varepsilon }(y)-u^{\varepsilon }(x))\rho ^{\varepsilon }(y)\,\textrm{d}y, \end{array}\right. } \end{aligned}$$

on bounded intervals

1.10 $$\begin{aligned} \Omega _T^\varepsilon =\big \{(t,x)\ :\, b^-_\varepsilon (t)\leqq r\leqq b^+_\varepsilon (t),\,0\leqq t\leqq T \big \}, \end{aligned}$$

with the free boundaries $b^\pm _\varepsilon (t)$, expanding to infinity as $\varepsilon \rightarrow 0^+$.

In the above system, the viscosity coefficient is equal to $\varepsilon \mu (\rho )=\varepsilon \rho ^{\alpha },$ $\alpha \geqq 0$, and the parameter $\varepsilon \in (0,1]$ denotes the inverse of the Reynolds number. A derivation of CNSEs (1.9) from the Boltzmann equations (without the nonlocal term) may be found in Liu-Xin-Yang [37], in which the viscosity is not constant but depends on the temperature. This dependence can be translated into the dependence of the viscosity on the density for the isentropic flow.

### The state of the art

There is a huge literature devoted to the study of the existence of solutions to CEEs either via the analysis of the vanishing artificial viscosity limit (see [2, 24, 30] for instance), or by constructing the finite difference scheme. The global existence of solutions with large initial data in $L^{\infty }$ was first established by DiPerna [27] for $\gamma =1+\frac{2}{2n+1}$ with $n\geqq 2$ integer by using the artificial viscosity method for the density equation. For the general interval $1<\gamma \leqq \frac{5}{3},$ the global existence problem was solved by Ding-Chen-Luo [25, 26] and Chen [12] by approximation via the Lax-Friedrichs scheme. The case of adiabatic exponent $\gamma >\frac{5}{3}$ was covered by Lions-Perthame-Tadmor [36] and Lions-Perthame-Souganidis [35] via the vanishing viscosity method. We also refer to [34] for blow-up results on CEEs and to Chen-LeFloch [15, 16] for relevant results on CEEs with general pressure law.

Concerning approximation by CNSEs, the $L^{\infty }$ estimates for the approximate solutions are not expected when the initial data only are of finite energy. Therefore, we work with the finite-energy framework, which was first considered by LeFloch-Westdickenberg [33] for $1<\gamma <\frac{5}{3}$, and was late developed by Chen-Perepelitsa [17] to the whole physical range of adiabatic exponents $\gamma >1.$ In particular, they established a compensated compactness framework (based on the earlier work by Tartar [45] and Murat [39]) and proved the vanishing viscosity limit of the solutions of the 1-D CNSEs to the corresponding finite-energy solutions of CEEs for large initial data for all $\gamma >1$ in [17]. In this result, the initial density at the far-field is allowed to be positive, and the viscosity is independent of the density, *i.e.,* $\alpha =0.$ This framework has been subsequently extended by Chen-Schrecker [19] and Chen-Wang [20] to study spherically symmetric Euler equations. More recently, Chen-He-Wang-Yuan [13] established the global existence of finite-energy solutions of the multi-dimensional Euler-Poisson equations for both compressible gaseous stars and plasmas with large spherically symmetric initial data. Adapting the approach developed in [13], He-Wang [29] proved the vanishing viscosity limit for the 1-D CNSEs. For CEEs with general pressure law, Shrecker-Schulz [41, 42] proved the vanishing viscosity limit for the 1-D CNSEs under asymptotically isothermal assumptions. More recently, Chen-Huang-Li-Wang-Wang [14] proved the vanishing physical viscosity limit for CNSPEs with general pressure law for large spherically symmetric initial data, in which an $L^p$ compensated compactness framework for general pressure law was also established. Finally, the existence of solutions for a general class of density-degenerate viscous models has been studied in [22, 23].

The main challenges tackled in this paper in comparison to the previous results are due to the presence of the nonlocal terms. The existence of global-in-time solutions to the nonlocal Euler system (1.1) on the whole line has never been proven before. In contrast, it is known that the classical solutions of (1.1) without pressure and alignment, *i.e.,* $P(\rho )=\varpi =0$, may blow up in finite time [6] and that they can be approximated by a degenerate vanishing viscosity method [9]. More recently, Carrillo-Galtung [10] showed that, for such pressureless Euler systems in 1-D, the Lagrangian and entropy solutions are equivalent, which was then used to explore the long-time-asymptotics of the solutions. Moreover, Chaudhuri *et al.* [11] studied the two-velocity reformulation of system (1.9) and derived an energy-type inequality, in the spirit of the Bresch-Desjardins estimate [3, 4]. This was then used to construct the weak solutions and to study their long-time behavior leading to the same density profiles as in [6] or [10]. We also consider the alignment terms but only for bounded communication kernels. The global existence of solutions with initial data of bounded variation (BV) in one dimension is proven by Amadori-Christoforou [1] for all-to-all coupled cases leading to flocking behavior. More general alignment kernels as in [43, 44] are also interesting and challenging questions that are not covered in this work.

The weak solutions to CNSEs with nonlocal attraction-repulsion forces in three dimensions were recently constructed in [38]; see also [8] for the constant viscosity case, where the long-time behavior of the solutions was considered. Finally, global-in-time well-posedness theory for the Euler equations with Riesz interactions and linear damping was recently established in [21] for the initial data near the equilibrium state and on the torus. We construct global entropy solutions to (1.1) via global weak solutions to the nonlocal Navier–Stokes system (1.9) with stress-free boundary conditions. The proof that these solutions are uniformly bounded, in terms of $\varepsilon$, despite the presence of nonlocal terms is another novelty. A new procedure for approximating the initial data, with the bounded second moment of the density, is implemented to obtain the propagation in time of the second moment of the density so that the confinement is controlled; see Lemma 3.2.

This paper has the following structure: in §2, we first introduce the definitions of finite-energy entropy solutions for CEEs with nonlocal interactions and present the main result—Theorem 2.2. We then describe the construction of the approximate solutions for CNSEs and state the inviscid limit theorem, Theorem 2.3. The rest of the paper is then devoted to the proof of this result for two types of equations of state: the polytropic equation of state (1.5), and the general pressure satisfying hypotheses ($$\mathcal {H}$$). In §3, we derive the basic estimates for both the density and the velocity, and prove the $$H^{-1}_\textrm{loc}$$–compactness of entropy dissipation measures for the approximate solutions for the polytropic case. In §4, we prove the global existence of finite-energy entropy weak solutions for this case, $$ {i.e.},$$ we prove Theorem 2.3. In §5, we develop the arguments from §3 and §4 to prove the global existence of finite-energy entropy solutions for CEEs for the general pressure law case. In this case, we prove the inviscid limit by proving the $$W^{-1,p}_\textrm{loc}$$ compactness of entropy dissipation measures for the approximate solutions for $$1\leqq p<2$$. Finally, in Appendix A, we explain how the approximate initial data sequence $$(\rho ^\varepsilon _0,\rho ^\varepsilon _0u^{\varepsilon }_0)(x)$$ can be constructed with desired estimates, regularity, and boundary compatibility for the polytropic equation of state.

## Definitions and Main Theorems

In this section, we define the notion of entropy solutions of system (1.1) and then formulate our main results of this paper.

### Entropy and entropy flux pairs for CEEs

Recall that a pair $(\eta (\rho ,m),q(\rho ,m))$ is called an entropy-entropy flux pair (entropy pair, for short) of the Euler system (1.1) if it satisfies

2.1 $$\begin{aligned} \nabla q(\rho ,m)=\nabla \eta (\rho ,m)\nabla \Big (\begin{matrix}m\\ \frac{m^2}{\rho }+P(\rho )\end{matrix}\Big ); \end{aligned}$$

see Lax [32]. Furthermore, $\eta (\rho ,m)$ is called a weak entropy if

$$\begin{aligned} \eta |_{\rho =0}=0\qquad \,\, \text {for any fixed }u=\frac{m}{\rho }. \end{aligned}$$

From [36], it is well known (*cf*. [17, 18, 36]) that, for the polytropic case, any weak entropy $(\eta ,q)$ can be represented by

2.2 $$\begin{aligned} {\left\{ \begin{array}{ll} \displaystyle \eta ^\psi (\rho ,m)=\eta (\rho ,\rho u)=\int _{\mathbb {R}}\chi (\rho ;s-u)\psi (s)\,\textrm{d}s,\\ \displaystyle q^\psi (\rho ,m)=q(\rho ,\rho u)=\int _{\mathbb {R}}(\theta s+(1-\theta )u)\chi (\rho ;s-u)\psi (s)\, \textrm{d}s, \end{array}\right. } \end{aligned}$$

where $\chi (\rho ;s-u)=[\rho ^{2\theta }-(s-u)^2]_{+}^{\mathfrak {b}}$ with $\mathfrak {b}=\frac{3-\gamma }{2(\gamma -1)}>-\frac{1}{2}$ and $\theta =\frac{\gamma -1}{2}$ is the weak entropy kernel. In particular, when $\psi (s)=\frac{1}{2}s^2,$ the entropy pair is the pair of the mechanical energy and the associated flux:

$$\begin{aligned} \eta ^{*}(\rho ,m)=\frac{m^2}{2\rho }+\rho e(\rho ),\quad q^{*}(\rho ,m)=\frac{m^3}{2\rho ^2}+m (\rho e(\rho ))'. \end{aligned}$$

From (2.1), any entropy satisfies that

2.3 $$\begin{aligned} \eta _{\rho \rho }-\frac{P'(\rho )}{\rho ^{2}}\eta _{uu}=0, \end{aligned}$$

with $u=\frac{m}{\rho }$. It has been proved in [15, 16, 35, 36] that any regular weak entropy can be generated by the convolution of a smooth function $\psi (x)$ with a fundamental solution $\chi (\rho ,u,s)$ of the entropy equation (2.3), *i.e.*,

2.4 $$\begin{aligned} \eta ^{\psi }(\rho ,u)=\int _{\mathbb {R}}\chi (\rho ,u,s)\psi (s)\,\textrm{d}s. \end{aligned}$$

The corresponding entropy flux is generated from the flux kernel $\sigma (\rho ,u,s)$ (see (2.7), below), *i.e.*,

2.5 $$\begin{aligned} q^{\psi }(\rho ,u)=\int _{\mathbb {R}}\sigma (\rho ,u,s)\psi (s)\,\textrm{d}s. \end{aligned}$$

The entropy kernel $\chi =\chi (\rho ,u,s)$ is a fundamental solution of the entropy equation (2.3):

2.6 $$\begin{aligned} \left\{ \begin{aligned} \displaystyle&\chi _{\rho \rho }-\frac{P'(\rho )}{\rho ^2}\chi _{uu}=0,\\ \displaystyle&\chi \vert _{\rho =0}=0,\quad \chi _{\rho }\vert _{\rho =0}=\delta _{u=s}. \end{aligned} \right. \end{aligned}$$

As pointed out in [15], equation (2.6) is invariant under the Galilean transformation, which implies that $\chi (\rho ,u,s)=\chi (\rho ,u-s,0)=\chi (\rho ,0,s-u)$. For simplicity, we write it as $\chi (\rho ,u,s)=\chi (\rho ,u-s)$ below when no confusion arises. The corresponding entropy flux kernel $\sigma (\rho ,u,s)$ satisfies the Cauchy problem for $\sigma -u\chi$:

2.7 $$\begin{aligned} \left\{ \begin{aligned} \displaystyle&(\sigma -u\chi )_{\rho \rho }-\frac{P'(\rho )}{\rho ^2}(\sigma -u\chi )_{uu}=\frac{P''(\rho )}{\rho }\chi _{u},\\ \displaystyle&(\sigma -u\chi )\vert _{\rho =0}=0,\quad (\sigma -u\chi )_{\rho }\vert _{\rho =0}=0. \end{aligned} \right. \end{aligned}$$

It can be checked that $P(\rho )$, described by (1.2)–(1.4), satisfies all the conditions in [15, 16]. In particular, $\sigma -u\chi$ is Galilean invariant; see [15].

### Existence of solutions to CEEs

We are now ready to define the notion of solutions to system (1.1) and to formulate our main result.

#### Definition 2.1

A pair of functions $(\rho , m)(t,x)$ with $\rho \in L^\infty (\mathbb {R}_+; L^1_+(\mathbb {R}))$ and $\frac{m}{\sqrt{\rho }} \in L^\infty (\mathbb {R}_+; L^2(\mathbb {R}))$ for $\mathbb {R}_+:=(0,\infty )$ is a finite-energy entropy solution of the Cauchy problem (1.1) and (1.6), with $P(\rho )$ satisfying hypotheses ($\mathcal {H}$), if the following conditions hold:

(i) The total mass is conserved, *i.e.*,
  $$ \int _{\mathbb {R}} \rho (t,x)\,\textrm{d}x=\int _{\mathbb {R}} \rho _0(x)\,\textrm{d}x=:M \qquad \text{ a.e. } t\geqq 0, $$
  and $(\frac{m}{\sqrt{\rho }})(t,x)=0$ *a.e.* on the vacuum states $\{(t,x)\,:\, \rho (t,x)=0\}$.
(ii) For a.e. $t>0$, the total energy is not increasing:
  $$\begin{aligned} \int _{\mathbb {R}}\Big (\frac{1}{2} \Big |\frac{m}{\sqrt{\rho }}\Big |^2 +\rho e(\rho )+\frac{1}{2} \rho W*\rho \Big )(t,x)\, \textrm{d}x \leqq \mathcal {E}_0. \end{aligned}$$
(iii) For any $\Psi (t,x)\in C^1_0([0,\infty )\times \mathbb {R})$,
  2.8 $$\begin{aligned}&\int _{\mathbb {R}^{2}_+} \big (\rho \Psi _t + m\Psi _x\big )\,\textrm{d}x\textrm{d}t +\int _{\mathbb {R}} \rho _0(x) \Psi (0,x)\,\textrm{d}x=0, \end{aligned}$$
  2.9 $$\begin{aligned}&\int _{\mathbb {R}^{2}_+}\Big (m\Psi _t +\big (\frac{m^2}{\rho } +P(\rho )\big )\Psi _x\Big )\,\textrm{d}x\textrm{d}t +\int _{\mathbb {R}} m_0(x)\Psi (0,x)\,\textrm{d}x\nonumber \\&\quad =\int _{{\mathbb {R}^2_+}}\Big (\rho \partial _xW*\rho - \lambda m-\int _{\mathbb {R}}\varpi (x-y)\big (\rho (x) m(y)-\rho (y) m(x)\big )\,\textrm{d}y\Big ) \Psi \,\textrm{d}x\textrm{d}t, \end{aligned}$$
  where $$\mathbb {R}^{2}_+:=\mathbb {R}_+\times \mathbb {R}$$.
(iv) For any convex function $\psi (s)$ with subquadratic growth at infinity and any entropy pair $(\eta ^{\psi },q^{\psi })$ defined in (2.4)–(2.5),
  $$\begin{aligned}&\eta ^{\psi }(\rho ,m)_t+q^{\psi }(\rho ,m)_x\nonumber \\&-\eta ^{\psi }_m\Big (\lambda m+\rho \int _{\mathbb {R}}\varpi (x-y)\big (\rho (x) m(y)-\rho (y) m(x)\big )\,\textrm{d}y-\rho \partial _x W*\rho \Big )\leqq 0 \end{aligned}$$
  is satisfied in the sense of distributions.

We now state the main result of this paper.

#### Theorem 2.2

(Existence of solutions of CEEs with nonlocal interactions) Consider problem (1.1) with initial data (1.6) satisfying (1.7)–(1.8). Let $P(\rho )$ satisfy hypotheses ($\mathcal {H}$). Then there exists a global-in-time finite-energy entropy solution $(\rho , m )(t,x)$ of problem (1.1) and (1.6) in the sense of Definition 2.1. In particular, there exists a global-in-time finite-energy entropy solution for problem (1.1) and (1.6) with polytropic equation of state (1.5).

#### Remark 2.1

The interaction potential can also be extended to the more general form: $W(x)=-|x|+\frac{x^2}{2}+\tilde{W} (x),$ with $\tilde{W} (x)$ smooth and $|\tilde{W} (x)|\leqq C x^2$ at infinity. Even more, we can consider $W(x)=-|x|+\frac{x^2}{2}+\frac{|x|^{\nu }}{\nu }$ for $1<\nu <2.$

#### Remark 2.2

We can allow the communication kernel $\varpi (x)$ to be in $C(\mathbb {R}\backslash \{0\})$ with

$$ \varpi (x)\textbf{I}_{B(0,R)}\in L^{\frac{\gamma }{\gamma -1}}(\mathbb {R}), \quad \varpi (x)\textbf{I}_{\mathbb {R}\backslash B(0,R)}\in L^{\infty }(\mathbb {R}) \qquad \text {for any } R>0. $$

For example, we can choose the singular communication weight $\varpi (x)$ as $\varpi (x)=\frac{1}{|x|^{a}}$ for $a<\frac{\gamma -1}{\gamma }.$

#### Remark 2.3

Our results also hold for an asymptotically isothermal pressure law $(\gamma _2=1)$, that is, $P(\rho )/\rho = O(1)$ in the limit $\rho \rightarrow \infty$; see [41, 42].

### Existence of solutions to CNSEs and their inviscid limit

In this paper, we do not prove Theorem 2.2 directly. Instead, the solution from Definition 2.1 is obtained as a limit of the regular solutions of CNSEs (1.9) on a truncated domain $\Omega _T^\varepsilon$ with the moving boundary. We define that

2.10 $$\begin{aligned} \Omega _T^\varepsilon =\big \{(t,x)\ :\, b^-_\varepsilon (t)\leqq r\leqq b^+_\varepsilon (t),\,0\leqq t\leqq T \big \}, \end{aligned}$$

where $\{x=b^{\pm }_\varepsilon (t)\,:\,0<t\leqq T\}$ are the free boundaries determined by

2.11 $$\begin{aligned} \displaystyle \frac{\textrm{d}}{\textrm{d}t}b^\pm _\varepsilon (t)=u^\varepsilon (t,b^\pm _\varepsilon (t)), \quad b^\pm _\varepsilon (0)=\pm b_\varepsilon \qquad \quad \text{ for } t>0. \end{aligned}$$

On the free boundaries $x=b^\pm _\varepsilon (t)$, the stress-free boundary condition is imposed:

2.12 $$\begin{aligned} \big (P(\rho ^{\varepsilon })-\varepsilon \mu (\rho ^{\varepsilon }) u^{\varepsilon }_x\big )(t,b^\pm _\varepsilon (t)) =0 \qquad \text{ for } t>0. \end{aligned}$$

The initial intervals $[-b_\varepsilon , b_\varepsilon ]$ approximate the whole line when $\varepsilon \rightarrow 0^+$. More specifically, we assume explicitly that

$$ b_\varepsilon =\varepsilon ^{-p}\qquad \text {with}\,\,\,\,\, p>\left\{ \begin{array}{ll}\frac{\gamma }{\gamma -\alpha } &  \quad \text {for polytropic gases,}\\ \frac{\gamma _1}{\gamma _1-\alpha }&  \quad \text {for general pressure law.} \end{array}\right. $$

Let $(\rho _0, m_0)(x)$ satisfy (1.7)–(1.8). The initial data for the approximate system are given by

2.13 $$\begin{aligned} (\rho ^{\varepsilon },\rho ^{\varepsilon } u^{\varepsilon })(0,x) =(\rho _0^{\varepsilon },\rho _0^{\varepsilon }u_0^{\varepsilon })(x)\qquad \,\text{ for } x\in [-b_\varepsilon ,b_\varepsilon ], \end{aligned}$$

where $(\rho _0^{\varepsilon }, u_0^{\varepsilon })(x)$ are obtained by the smooth approximation of the original data $(\rho _0,m_0)$, constructed in Appendix A. In particular, they satisfy Lemmas A.1–A.6 from Appendix A.

We further define that

2.14 $$\begin{aligned} \mathcal {E}_{0}^{\varepsilon }:&= \int _{-b_\varepsilon }^{b_\varepsilon }\Big (\frac{1}{2}\rho _0^{\varepsilon }|u_0^{\varepsilon }|^2+ \rho _0^{\varepsilon }e(\rho _0^{\varepsilon })+\frac{1}{2}\rho _0^{\varepsilon }\,W*\rho ^{\varepsilon }_0\Big )\, \textrm{d}x,\,\,\,&\end{aligned}$$

2.15 $$\begin{aligned} \mathcal {E}_1^{\varepsilon } :&= \varepsilon ^2 \int _{-b_\varepsilon }^{b_\varepsilon } (\rho ^{\varepsilon }_0)^{2\alpha -3}|\rho ^{\varepsilon }_{0x}|^2\,\textrm{d}x, \end{aligned}$$

where the convolution in (2.14) is understood as $\int ^{b_\varepsilon }_{-b_\varepsilon }W(x-y)\rho _0^{\varepsilon }(y)\,\textrm{d}y$.

We are now ready to state the main theorem of our paper.

#### Theorem 2.3

(Inviscid limit for CNSEs with nonlocal interactions) Let the hypotheses of Theorem 2.2 be satisfied. Let $\gamma _1\geqq \gamma _2>1$ and $\frac{2}{3}<\alpha \leqq 1$, and let $\{(\rho ^{\varepsilon },\rho ^{\varepsilon }u^{\varepsilon })\}_{\varepsilon \in (0,1]}$ be a sequence of the strong solutions to (1.9) with initial and boundary data specified in (2.10)–(2.13) and satisfying

$$\begin{aligned}&0< C^{-1}_{\varepsilon }\leqq \rho ^{\varepsilon }_0(x)\leqq C_{\varepsilon },\quad (\rho ^{\varepsilon }_0(b))^{\gamma _1} b_\varepsilon = (\rho ^{\varepsilon }_0(-b_\varepsilon ))^{\gamma _1} b_\varepsilon \leqq C_0 \qquad \text{ for } \varepsilon \in (0,1],\nonumber \\&(\rho ^\varepsilon _0,\rho _0^\varepsilon u^{\varepsilon }_0)(x) \rightarrow (\rho _0,m_0)(x) \quad \text{ as } \varepsilon \rightarrow 0^+ \text{ in } L^{q}_\textrm{loc}(\mathbb {R})\times L^1_\textrm{loc}(\mathbb {R}) \text{ for } q\in \{1,\gamma _2\},\nonumber \\&(\mathcal {E}_0^\varepsilon ,\mathcal { E}_1^\varepsilon )\rightarrow (\mathcal {E}_0,0) \qquad \text{ as } \varepsilon \rightarrow 0^+, \nonumber \\&\int _{-\infty }^{\infty }\rho ^\varepsilon _0(x)\,\textrm{d}x= M, \quad \mathcal {E}^{\varepsilon }_0+\varepsilon ^{-1}\mathcal {E}_1^\varepsilon \le C_0, \quad \nonumber \\&\int ^{\infty }_{-\infty }x^2\rho ^{\varepsilon }_0(x)\,\textrm{d}x\,\rightarrow \,\int ^{\infty }_{-\infty }x^2\rho _0 (x)\,\textrm{d}x=M_2\qquad \text{ as } \varepsilon \rightarrow 0^+,\nonumber \end{aligned}$$

where $C_0>0$ is a constant independent of $\varepsilon \in (0,1]$ which may depend on $(\mathcal {E}_0, M, M_2, \gamma ,\alpha )$, while $C_\varepsilon >0$ is a constant depending on $\varepsilon >0$. Then there exist both a subsequence (still denoted) $(\rho ^{\varepsilon },m^{\varepsilon } )(t,x)$ with $m^{\varepsilon }=\rho ^\varepsilon u^\varepsilon$ and a vector-valued function $(\rho , m)(t,x)$ such that, as $\varepsilon \rightarrow 0^+$,

$$\begin{aligned} \begin{aligned}&(\rho ^{\varepsilon },m^{\varepsilon })(t,x)\rightarrow (\rho ,m)(t,x)\qquad \text { a.e. } (t,x)\in \mathbb {R}_+\times \mathbb {R},\\  &(\rho ^{\varepsilon },m^{\varepsilon })(t,x)\rightarrow (\rho ,m)(t,x)\qquad \text { in } L^{q_1}_\text {loc}(\mathbb {R}^2_+)\times L^{q_2}_\text {loc}(\mathbb {R}^2_+)\\  &\quad \quad \quad \quad \quad \quad \quad \quad \quad \quad \quad \quad \qquad \qquad \qquad \,\,\, \text { with } q_1\in [1,\gamma _2+1) \text { and } q_2\in [ 1,\frac{3(\gamma _2+1)}{\gamma _2+3}) . \end{aligned} \end{aligned}$$

Moreover, $(\rho ,m)(t,x)$ is a global finite-energy entropy solution of the Cauchy problem (1.1) and (1.6) in the sense of Definition 2.1. In particular, the result is true for the polytropic case (1.5).

#### Remark 2.4

In Theorem 2.3, we need $\frac{2}{3}<\alpha \leqq 1$; see (A10). However, note that the condition $\frac{2}{3}<\alpha <\gamma$ is needed only for obtaining the uniform estimates for the approximate solutions; see Lemmas 3.1–4.2 and Lemmas 5.2–5.10. In particular, our results cover the Saint-Venant model for shallow water ($\alpha =1$ and $\gamma =2$), even with the nonlocal terms.

#### Remark 2.5

The regular solutions for the compressible Navier–Stokes problem (1.9) with the new nonlocal terms and the stress-free boundary condition (2.11)–(2.12) can be obtained by following the arguments similar to [31, 40]; see also [11] for the Dirichlet boundary conditions.

## Uniform Estimates of Approximate Solutions

This section is dedicated to several uniform estimates with respect to $\varepsilon \in (0, 1]$ for the regular solutions of (1.9) on the bounded time-dependent domain (2.10) with corresponding boundary conditions. Although some of the estimates hold also for general $P(\rho )$ specified through hypotheses $(\mathcal {H})$, for the purposes of this section, we restrict ourselves to the polytropic equation of state (1.5). The general pressure case is discussed in §5.

We drop the $\varepsilon$-superscript of approximate solutions when no confusion arises. In all of the estimates from now on, $C>0$ is a universal constant independent of $\varepsilon$, which may depend on $\mathcal {E}_0, M, M_2, C_0,T,\gamma$, and $\alpha$, and can be different at each occurrence. For abbreviation, we also denote that

3.1 $$\begin{aligned} V=V(t,x):&=\int _{\mathbb {R}}\varpi (x-y)\Big (m(y)-\frac{m(x)}{\rho (x)}\rho (y)\Big )\,\textrm{d}y\nonumber \\&=\int ^{b^+(t)}_{b^-(t)}\varpi (x-y)\Big (m(y)-\frac{m(x)}{\rho (x)}\rho (y)\Big )\,\textrm{d}y \end{aligned}$$

for $$x\in (b^-(t),b^+(t))$$.

### Conservation of mass and Lagrangian coordinates

We present the reformulation of IBVP (1.9) and (2.10)–(2.13) in the so-called Lagrangian coordinates, which is equivalent to the Eulerian formulation for sufficiently regular solutions. It follows from (1.9)$_{1}$ and (2.11) that

$$\begin{aligned} \frac{\textrm{d}}{\textrm{d}t}\int _{b^-(t)}^{b^+(t)}\rho (t,x)\,\textrm{d}x= (\rho u)(t,b^+(t))-(\rho u)(t,b^-(t))-\int _{b^-(t)}^{b^+(t)}(\rho u )_x(t,x)\,\textrm{d}x=0, \end{aligned}$$

which, due to (A21), yields that

3.2 $$\begin{aligned} \int _{b^-(t)}^{b^+(t)}\rho (t,x)\,\textrm{d}x=\int _{b^-(t)}^{b^+(t)}\rho ^{\varepsilon }_0(x)\,\textrm{d}x= M \qquad \,\, \text{ for } \text{ any } t\geqq 0. \end{aligned}$$

For $x\in [b^-(t),b^+(t)]$ and $t\in [0,T]$, we define the Lagrangian coordinates $(\tau ,\xi )$ as

$$\begin{aligned} \xi =\int _{b^-(t)}^x \rho (t,y)\,\textrm{d}y,\qquad \tau =t, \end{aligned}$$

which transform the domain with the free boundary: $[0,T]\times [b^-(t),b^+(t)]$ into the fixed domain: $[0,T]\times [0,M]$. A direct calculation shows that

$$\begin{aligned} {\left\{ \begin{array}{ll} \displaystyle \frac{\partial \xi }{\partial t}=-\rho u,\quad \frac{\partial \xi }{\partial x}=\rho ,\quad \frac{\partial \tau }{\partial t}=1, \quad \frac{\partial \tau }{\partial x}=0,\\ \displaystyle \frac{\partial x}{\partial \tau }=u,\quad \frac{\partial x}{\partial \xi }=\frac{1}{\rho },\quad \frac{\partial t}{\partial \tau }=1,\quad \frac{\partial t}{\partial \xi }=0. \end{array}\right. } \end{aligned}$$

Applying this transformation, IBVP (1.9) and (2.10)–(2.13) becomes

3.3 $$\begin{aligned} {\left\{ \begin{array}{ll} \displaystyle \rho _\tau +\rho ^2u_{\xi }=0,\\ \displaystyle u_\tau + P(\rho )_\xi -\varepsilon (\mu (\rho )\rho u_{\xi })_{\xi }-\lambda u-V+\rho (W*\rho )_{\xi }=0 \end{array}\right. } \end{aligned}$$

for $$(\tau ,\xi )\in [0,T]\times [0,M]$$, and

3.4 $$\begin{aligned} \big (P(\rho )-\varepsilon \mu (\rho )\rho u_{\xi }\big )(\tau ,0)=0, \quad \big (P(\rho )-\varepsilon \mu (\rho )\rho u_{\xi }\big )(\tau ,M)=0 \quad \text{ for } \tau \in [0,T]. \end{aligned}$$

With a slight abuse of notation, we denote by *V* a function defined in (3.1), taken as a function of $\xi$. The fixed boundaries $\xi =0,M$ correspond to the free boundaries $x=b^{\pm }(t)$ in the Eulerian coordinates.

### Basic energy estimate

To begin with, we obtain the basic energy estimate for problem (1.9) and (2.10)–(2.13).

#### Lemma 3.1

For a smooth solution of problem (1.9) and (2.10)–(2.13), the following estimate holds:

3.5 $$\begin{aligned}&\int ^{b^+(t)}_{b^-(t)}\Big (\frac{1}{2}\rho u^2+\rho e(\rho ) +\frac{1}{2}\rho W*\rho \Big )(t,x)\,\textrm{d}x \nonumber \\&\quad +\int _0^t\int _{b^-(s)}^{b^+(s)} \left( \varepsilon \rho ^{\alpha }|u_x|^2-\lambda \rho u^2\right) (s,x)\,\textrm{d}x\textrm{d}s\nonumber \\&\quad +\frac{1}{2}\int ^t_0\int ^{b^+(s)}_{b^-(s)}\int ^{b^+(s)}_{b^-(s)}\varpi (x-y)\rho (x)\rho (y)|u(y)-u(x)|^2\,\textrm{d}y\textrm{d}x\textrm{d}s = \mathcal {E}^{\varepsilon }_0\le C_0. \end{aligned}$$

Here and hereafter, $C_0>0$ is the constant from the statement of Theorem 2.3, independent of $\varepsilon \in (0,1]$.

#### Proof

It follows from (3.3)$_{1}$ that

3.6 $$\begin{aligned} -P(\rho )u_{\xi }=\frac{P(\rho )}{\rho ^2}\rho _{\tau }=\kappa \rho ^{\gamma -2}\rho _{\tau }=e(\rho )_{\tau }. \end{aligned}$$

Multiplying (3.3)$_{2}$ by *u*,  we have

3.7 $$\begin{aligned}&\Big (\frac{u^2}{2}+e(\rho )\Big )_{\tau }+\varepsilon \rho ^{1+\alpha }(u_{\xi })^2 + \big ((P(\rho )-\varepsilon \mu (\rho )\rho u_{\xi })u\big )_{\xi }\nonumber \\&\,\,-\lambda u^2-u V+\rho u (W*\rho )_{\xi }=0. \end{aligned}$$

Notice that

$$\begin{aligned} -\int ^M_0u V\,\textrm{d}\xi&=-\int ^{b^+(t)}_{b^-(t)}\rho (x)u(x)\Big (\int ^{b^+(t)}_{b^-(t)}\varpi (x-y) \big (u(y)-u(x)\big )\rho (y)\,\textrm{d}y\Big )\textrm{d}x\nonumber \\&=\int ^{b^+(t)}_{b^-(t)}\int ^{b^+(t)}_{b^-(t)}\varpi (x-y)\rho (x) \rho (y)\big (|u(x)|^2-u(x)u(y)\big )\,\textrm{d}y\textrm{d}x\nonumber \\&=\frac{1}{2}\int ^{b^+(t)}_{b^-(t)}\int ^{b^+(t)}_{b^-(t)}\varpi (x-y)\rho (x)\rho (y)|u(y)-u(x)|^2\,\textrm{d}y\textrm{d}x,\nonumber \end{aligned}$$

where we have used the symmetry of $\varpi (x)$.

For the other nonlocal term, our aim is to prove that

3.8 $$\begin{aligned} \int ^M_0\rho u(W *\rho )_{\xi }\,\textrm{d}\xi =\frac{\textrm{d}}{\textrm{d}t}\int ^{b^+(t)}_{b^-(t)}\frac{1}{2}\rho W *\rho \,\textrm{d}x. \end{aligned}$$

We first rewrite the right-hand side (RHS) to obtain

3.9 $$\begin{aligned} \frac{\textrm{d}}{\textrm{d}t}\int ^{b^+(t)}_{b^-(t)}\frac{1}{2}\rho W*\rho \,\textrm{d}x&=\int ^{b^+(t)}_{b^-(t)}\frac{1}{2}(\rho W*\rho )_t\,\textrm{d}x +\frac{1}{2}(\rho W*\rho )(t,b^+(t))\frac{\textrm{d}b^+(t)}{\textrm{d}t} \nonumber \\&\quad \ -\frac{1}{2}(\rho W*\rho )(t,b^-(t))\frac{\textrm{d}b^-(t)}{\textrm{d}t}\nonumber \\&=\frac{1}{2}\int ^{b^+(t)}_{b^-(t)}\left( \rho _t W*\rho +\rho W*\rho _t\right) \,\textrm{d}x \nonumber \\&\quad \ +\frac{1}{2}(u\rho W*\rho )(t,b^+(t)) -\frac{1}{2}(u\rho W*\rho )(t,b^-(t)). \end{aligned}$$

Using the change of the order of integration and the explicit form of $W(x)=-|x|+\frac{|x|^2}{2}$ (in particular, $W(x-y)=W(y-x)$), we obtain

$$\begin{aligned} \int ^{b^+(t)}_{b^-(t)}\rho W*\rho _t\,\textrm{d}x&=\int ^{b^+(t)}_{b^-(t)}\rho (t,x)\big (\int ^{b^+(t)}_{b^-(t)}W(x-y)\rho (t,y)\,\textrm{d}y\big )_t\,\textrm{d}x\nonumber \\&=\int ^{b^+(t)}_{b^-(t)}\rho _t(t,y)\big (\int ^{b^+(t)}_{b^-(t)}W(x-y)\rho (t,x)\,\textrm{d}x\big )\,\textrm{d}y\nonumber \\&\quad \,+(\rho u W*\rho )(t,b^+(t))-(\rho u W*\rho )(t,b^-(t))\nonumber \\&=\int ^{b^+(t)}_{b^-(t)}\rho _t(t,y)\big (\int ^{b^+(t)}_{b^-(t)}W(y-x)\rho (t,x)\,\textrm{d}x\big )\,\textrm{d}y\nonumber \\&\quad \,+(\rho u W*\rho )(t,b^+(t))-(\rho u W*\rho )(t,b^-(t))\nonumber \\&=\int ^{b^+(t)}_{b^-(t)}\rho _t(t,x)\big (\int ^{b^+(t)}_{b^-(t)}W(x-y)\rho (t,y)\,\textrm{d}y\big )\,\textrm{d}x\nonumber \\&\quad \, +(\rho u W*\rho )(t,b^+(t))-(\rho u W*\rho )(t,b^-(t))\nonumber \\&=\int ^{b^+(t)}_{b^-(t)}\rho _t W*\rho \,\textrm{d}x\nonumber \\&\quad \,+(\rho u W*\rho )(t,b^+(t))-(\rho u W*\rho )(t,b^-(t)). \end{aligned}$$

Thus, it follows that the first term on the RHS of (3.9) is equal to

$$\begin{aligned} \frac{1}{2}\int ^{b^+(t)}_{b^-(t)}\big (\rho _t W*\rho +\rho W*\rho _t\big )\,\text {d}x&=\int ^{b^+(t)}_{b^-(t)}\rho _t W*\rho \,\text {d}x +\frac{1}{2}(\rho u W*\rho )(t,b^+(t)) \nonumber \\  &\quad \, -\frac{1}{2}(\rho u W*\rho )(t,b^-(t)). \end{aligned}$$

Therefore, we have proven that

3.10 $$\begin{aligned} \frac{\textrm{d}}{\textrm{d}t}\int ^{b^+(t)}_{b^-(t)}\frac{1}{2}\rho W*\rho \,\textrm{d}x&=\int ^{b^+(t)}_{b^-(t)}\rho _t W*\rho \,\textrm{d}x \nonumber \\&\quad \ +(u\rho W*\rho )(t,b^+(t)) -(u\rho W*\rho )(t,b^-(t)). \end{aligned}$$

On the other hand, it follows directly from (1.9)$_{1}$ that

3.11 $$\begin{aligned} \int ^M_0\rho u (W*\rho )_{\xi }\,\text {d}\xi&=\int ^{b^+(t)}_{b^-(t)}\rho u (W*\rho )_x\,\text {d}x\nonumber \\  &=(\rho u W*\rho )(t,b^+(t))-(\rho u W*\rho )(t,b^-(t))\nonumber \\  &\quad \, -\int ^{b^+(t)}_{b^-(t)}(\rho u)_x W*\rho \,\text {d}x\nonumber \\  &=(\rho u W*\rho )(t,b^+(t))-(\rho u W*\rho )(t,b^-(t))\nonumber \\  &\quad \, +\int ^{b^+(t)}_{b^-(t)}\rho _t W*\rho \,\text {d}x. \end{aligned}$$

Comparing (3.10) with (3.11), we deduce (3.8).

Since $W=-|x|+\frac{|x|^2}{2},$ we see that $W+\frac{1}{2}\geqq 0.$ Then, using (3.2), we obtain

$$ \frac{\textrm{d}}{\textrm{d}t}\int ^{b^+(t)}_{b^-(t)}\frac{1}{2}\rho W*\rho \,\textrm{d}x=\frac{\textrm{d}}{\textrm{d}t}\int ^{b^+(t)}_{b^-(t)}\frac{1}{2}\rho \Big (W+\frac{1}{2}\Big )*\rho \,\textrm{d}x. $$

Integrating (3.7) over $[0,\tau ]\times [0,M]$, using the Grönwall inequality and the stress-free boundary condition (3.4), pulling the resultant equation back to the Eulerian coordinates, we obtain (3.5). This completes the proof. $\square$

### Second moment estimate

We now derive the second-moment estimate for the density.

#### Lemma 3.2

There exists $C>0$, independent of $\varepsilon$, such that

3.12 $$\begin{aligned} \int ^{b^+(t)}_{b^-(t)}x^2\rho (t,x) \,\textrm{d}x\leqq C. \end{aligned}$$

#### Proof

Using (1.9)$_{1}$, we have

$$\begin{aligned}&\frac{\text {d}}{\text {d}t}\int ^{b^+(t)}_{b^-(t)}x^2\rho \,\text {d}x\\  &=(b^+(t))^2(\rho u)(t,b^+(t)) -(b^- (t))^2(\rho u)(t,b^-(t)) +\int ^{b^+(t)}_{b^-(t)} x^2\rho _t\,\text {d}x\\  &=2\int ^{b^+(t)}_{b^-(t)} x\rho u\,\text {d}x, \end{aligned}$$

such that

$$ \frac{\textrm{d}}{\textrm{d}t}\int ^{b^+(t)}_{b^-(t)}x^2\rho \,\textrm{d}x \leqq \int ^{b^+(t)}_{b^-(t)}x^2\rho \,\textrm{d}x+\int ^{b^+(t)}_{b^-(t)}\rho u^2 \,\textrm{d}x. $$

Then we conclude (3.12) by using the Grönwall inequality. $\square$

### Higher-order estimates of the density and the pressure

In this section, we derive several higher-order estimates for the density and the pressure. To start, we analyze the behavior of density $\rho$ on the free boundary. It follows from (3.3)$_{1}$ and (3.4) that

$$\begin{aligned} \frac{\textrm{d}}{\textrm{d}\tau }(\rho ^{\alpha -\gamma }(\tau ,M))=-\frac{\kappa (\alpha -\gamma )}{\varepsilon }. \end{aligned}$$

Then we have

$$\begin{aligned} \rho (\tau ,M)=\rho _0(M)\,\Big (1+\frac{\kappa (\gamma -\alpha )}{\varepsilon }(\rho _0(M))^{\gamma -\alpha }\tau \Big )^{-\frac{1}{\gamma -\alpha }}, \end{aligned}$$

so that

$$\begin{aligned} \rho (t,b^+(t))=\rho _0(b)\,\Big (1+\frac{\kappa (\gamma -\alpha )}{\varepsilon }(\rho _0(b))^{\gamma -\alpha }t\Big )^{-\frac{1}{\gamma -\alpha }}. \end{aligned}$$

We obtain

3.13 $$\begin{aligned} \rho (t,b^+(t))=\rho (t,b^-(t))=\rho _0(b)\Big (1+\frac{\kappa (\gamma -\alpha )}{\varepsilon }(\rho _0(b))^{\gamma -\alpha }t\Big )^{-\frac{1}{\gamma -\alpha }}\leqq \rho _0(b), \end{aligned}$$

where we have used that $\rho _0(b)=\rho _0(-b).$ From (3.13), it follows, in particular, that the third and fourth term on the left-hand side (LHS) of (3.14) below is nonnegative. We have the following result:

#### Lemma 3.3

Let $\alpha \geqq 0$ and $\rho ^{\gamma }_0(\pm b)b\leqq C_0$. Then, for any given $T>0$, there exists $C>0$ such that, for any $t\in [0,T]$,

3.14 $$\begin{aligned}&\varepsilon ^2\int ^{b^+(t)}_{b^-(t)}(\rho ^{2\alpha -3}\rho ^2_x)(t,x)\,\textrm{d}x +\varepsilon \kappa \gamma \int ^t_0\int ^{b^+(t)}_{b^-(t)}(\rho ^{\alpha +\gamma -3}\rho ^2_x)(s,x)\,\textrm{d}x\textrm{d}s\nonumber \\&+\frac{\kappa \gamma }{\varepsilon }\int ^{t}_0 \Big (\rho ^{2\gamma -\alpha }(s,b^+(s))b^+(s)-\rho ^{2\gamma -\alpha }(s,b^-(s))b^-(s)\Big )\,\textrm{d}s\nonumber \\&+\kappa \Big (\rho ^{\gamma }(t,b^+(t))b^+(t)-\rho ^{\gamma }(t,b^-(t))b^-(t)\Big )\leqq C . \end{aligned}$$

#### Proof

It is direct to calculate from (3.3)$_{2}$ that

3.15 $$\begin{aligned} \Big (u+\frac{\varepsilon }{\alpha }(\rho ^{\alpha })_{\xi }\Big )_{\tau }+P(\rho )_{\xi }-\lambda u-V+\rho (W*\rho )_{\xi }=0, \end{aligned}$$

where we have used the fact that

$$\begin{aligned} \mu (\rho )\rho u_{\xi }=\rho ^{1+\alpha }u_{\xi }=-\rho ^{\alpha -1}\rho _{\tau }=-\frac{1}{\alpha }(\rho ^{\alpha })_{\tau }. \end{aligned}$$

Multiplying (3.15) by $u+\frac{\varepsilon }{\alpha }(\rho ^{\alpha })_{\xi },$ we obtain

3.16 $$\begin{aligned}&\Big (\frac{(u+\frac{\varepsilon }{\alpha }(\rho ^{\alpha })_{\xi })^2}{2} +\frac{\kappa \rho ^{\gamma -1}}{\gamma -1}+\frac{1}{2}\rho W*\rho \Big )_{\tau } +\big (p(\rho )u\big )_{\xi }+\varepsilon \kappa \gamma \rho ^{\alpha +\gamma -2}(\rho _{\xi })^2\nonumber \\&+\big (u+\frac{\varepsilon }{\alpha }(\rho ^{\alpha })_{\xi }\big )\big (-\lambda u-V+\rho (W*\rho )_{\xi }\big )=0. \end{aligned}$$

Using (3.3)$_{1}$ and (3.4), we have

$$\begin{aligned} \rho _{\tau }(\tau ,M)=-\frac{\kappa }{\varepsilon }\rho ^{\gamma +1-\alpha }(\tau ,M), \qquad \rho _{\tau }(\tau ,0)=-\frac{\kappa }{\varepsilon }\rho ^{\gamma +1-\alpha }(\tau ,0). \end{aligned}$$

Then we obtain

$$\begin{aligned}&\big (P(\rho )u\big )(\tau ,M)-\big (P(\rho )u\big )(\tau ,0)\nonumber \\&=\kappa \rho ^{\gamma }(\tau ,M)\frac{\textrm{d}b^+(\tau )}{\textrm{d}\tau }-\kappa \rho ^{\gamma }(\tau ,0)\frac{\textrm{d}b^-(\tau )}{\textrm{d}\tau }\nonumber \\&=\kappa \big (\rho ^{\gamma }(\tau ,M)b^+(\tau )\big )_{\tau } -\kappa \big (\rho ^{\gamma }(\tau ,0)b^-(\tau )\big )_{\tau }\nonumber \\&\quad +\kappa \gamma \Big (\rho ^{\gamma -1}(\tau ,0)\rho _{\tau }(\tau ,0)b^{-}(\tau )-\rho ^{\gamma -1}(\tau ,M)\rho _{\tau }(\tau ,M)b^{+}(\tau )\Big )\nonumber \\&=\kappa \big (\rho ^{\gamma }(\tau ,M)b^+(\tau )\big )_{\tau }-\kappa \big (\rho ^{\gamma }(\tau ,0)b^-(\tau )\big )_{\tau }\nonumber \\&\quad +\frac{\kappa \gamma }{\varepsilon }\Big (\rho ^{2\gamma -\alpha }(\tau ,M)b^+(\tau )-\rho ^{2\gamma -\alpha }(\tau ,0)b^-(\tau )\Big ).\nonumber \end{aligned}$$

Integrating (3.16) over [0, *M*] yields

3.17 $$\begin{aligned}&\frac{\textrm{d}}{\textrm{d}\tau }\int ^{M}_0\Big (\frac{1}{2}\big (u+\frac{\varepsilon }{\alpha }(\rho ^{\alpha })_{\xi }\big )^2 +\frac{\kappa \rho ^{\gamma -1}}{\gamma -1}\Big )\,\textrm{d}\xi +\varepsilon \kappa \gamma \int ^M_0\rho ^{\alpha +\gamma -2}(\rho _{\xi })^2\,\textrm{d}\xi \nonumber \\&\qquad +\kappa \Big (\rho ^{\gamma }(\tau ,M)b^+(\tau )-\rho ^{\gamma }(\tau ,0)b^-(\tau )\Big )_{\tau } \nonumber \\&\qquad +\frac{\kappa \gamma }{\varepsilon }\Big (\rho ^{2\gamma -\alpha }(\tau ,M)b^+(\tau )-\rho ^{2\gamma -\alpha }(\tau ,0)b^-(\tau )\Big )\nonumber \\&=\int ^M_0\big (u+\frac{\varepsilon }{\alpha }(\rho ^{\alpha })_{\xi }\big )\big (-\lambda u-V+\rho (W*\rho )_{\xi }\big )\,\textrm{d}\xi . \end{aligned}$$

For the last term in (3.17), we have

$$\begin{aligned}&\int ^M_0\big (u+\frac{\varepsilon }{\alpha }(\rho ^{\alpha })_{\xi }\big )\big (-\lambda u-V+\rho (W*\rho )_{\xi }\big )\,\textrm{d}\xi \nonumber \\&\leqq \int ^M_0\big (u+\frac{\varepsilon }{\alpha }(\rho ^{\alpha })_{\xi }\big )^2\,\textrm{d}\xi +\int ^M_0\big (-\lambda u-V+\rho (W*\rho )_{\xi }\big )^2\,\textrm{d}\xi \nonumber \\&\leqq \int ^M_0\big (u+\frac{\varepsilon }{\alpha }(\rho ^{\alpha })_{\xi }\big )^2\,\textrm{d}\xi +C\int ^M_0u^2\,\textrm{d}\xi +C\int ^M_0 V^2\,\textrm{d}\xi + C\int ^M_0\big (\rho (W*\rho )_{\xi }\big )^2\,\textrm{d}\xi \nonumber \\&\leqq \int ^M_0\big (u+\frac{\varepsilon }{\alpha }(\rho ^{\alpha })_{\xi }\big )^2\,\textrm{d}\xi + C\int ^{b^+(t)}_{b^-(t)}\rho \big (\partial _xW*\rho \big )^2\,\textrm{d}x+C,\nonumber \end{aligned}$$

where we have used the fact that

$$\begin{aligned} \int ^M_0 V^2\,\textrm{d}\xi&=\int ^{b^+(t)}_{b^-(t)}\rho V^2\,\textrm{d}x \\&=\int ^{b^+(t)}_{b^-(t)}\rho (t,x)\Big (\int ^{b^+(t)}_{b^-(t)}\varpi (x-y)\big (u(t,y)-u(t,x)\big )\rho (t,y)\,\textrm{d}y\Big )^2\,\textrm{d}x \\&\leqq C\int ^{b^+(t)}_{b^-(t)}\bigg (\rho (t,x)\Big (\int ^{b^+(t)}_{b^-(t)}\big (\rho (t,y)+(\rho u^2)(t,y)\big )\,\textrm{d}y\Big )^2 +u^2(t,x)\bigg )\,\textrm{d}x \\&\leqq C.\nonumber \end{aligned}$$

Notice that

3.18 $$\begin{aligned} (\partial _xW*\rho )(t,x) =\int ^{b^+(t)}_{b^-(t)}\big (1-2H(x-y)+x-y\big )\rho (t,y)\,\textrm{d}y, \end{aligned}$$

where $H(\cdot )$ is the Heaviside function. We now rewrite the RHS of (3.18) by using

$$\begin{aligned} \int ^{b^+(t)}_{b^-(t)}\rho (t,y)\,\textrm{d}y=M,\qquad \int ^{b^+(t)}_{b^-(t)}H(x-y)\rho (t,y)\,\textrm{d}y=\int ^x_{b^-(t)}\rho (t,y)\,\textrm{d}y. \end{aligned}$$

We obtain

$$\begin{aligned} (\partial _xW*\rho )(t,x)=M-2\int ^x_{b^-(t)}\rho (t,y)\,\textrm{d}y+xM-\int ^{b^+(t)}_{b^-(t)}y\rho (t,y)\,\textrm{d}y. \end{aligned}$$

It follows from (3.2) and (3.12) that

$$\Big |\int ^{b^+(t)}_{b^-(t)}|x|\rho (t,x) \,\textrm{d}x\Big |\leqq \int ^{b^+(t)}_{b^-(t)}(x^2+1)\rho (t,x) \,\textrm{d}x\leqq C.$$

Then we have

3.19 $$\begin{aligned}&\int ^{b^+(t)}_{b^-(t)}\rho (t,x)(\partial _xW*\rho )^2(t,x)\,\textrm{d}x\nonumber \\&\leqq \int ^{b^+(t)}_{b^-(t)}\rho \Big (3M+|x|M+\int ^{b^+(t)}_{b^-(t)}|y|\rho (t,y)\,\textrm{d}y\Big )^2\,\textrm{d}x\leqq C. \end{aligned}$$

Integrating (3.16) over $[0,\tau ]$ leads to

$$\begin{aligned}&\int ^M_0\Big (\frac{1}{2}\big (u+\frac{\varepsilon }{\alpha }(\rho ^{\alpha })_{\xi }\big )^2 +\frac{\kappa }{\gamma -1}\rho ^{\gamma -1}\Big )\,\textrm{d}\xi +\varepsilon \kappa \gamma \int ^{\tau }_0\int ^M_0\rho ^{\alpha +\gamma -2}(\rho _{\xi })^2\,\textrm{d}\xi \textrm{d}s\nonumber \\&\,\,\,+\kappa \Big (\rho ^{\gamma }(\tau ,M)b^+(\tau )-\rho ^{\gamma }(\tau ,0)b^-(\tau )\Big )\nonumber \\&\quad +\frac{\kappa \gamma }{\varepsilon }\int ^{\tau }_0\Big (\rho ^{2\gamma -\alpha }(s,M)b^+(s)-\rho ^{2\gamma -\alpha }(s,0)b^-(s)\Big )\,\textrm{d}s\nonumber \\&\leqq C\int ^M_0\Big (\frac{1}{2}\big (u_0+\frac{\varepsilon }{\alpha }(\rho ^{\alpha }_0)_{\xi }\big )^2 +\frac{\kappa }{\gamma -1}\rho ^{\gamma -1}_0\Big )\,\textrm{d}\xi +\kappa \big (\rho ^{\gamma }_0(M)+\rho ^{\gamma }_0(0)\big )b+C.\nonumber \end{aligned}$$

This completes the proof. $\square$

Our next aim is to show that domain $\Omega _T$ expands to the whole physical space $[0,T]\times \mathbb {R}$, that is, $\displaystyle \inf _{t\in [0,T]}b(t)\rightarrow \infty$ as $b\rightarrow \infty$. We have the following result:

#### Lemma 3.4

([29], Lemma 2.3) Choose $0\leqq \alpha <\gamma ,$ $p>\frac{\gamma }{\gamma -\alpha },$ and $b:=\varepsilon ^{-p}.$ Then, for any given $T>0$, there exists $\varepsilon _0>0$ such that, for $\varepsilon \in (0,\varepsilon _0]$,

$$\begin{aligned} \pm b^\pm (t)\geqq \frac{1}{2} b \qquad \text{ for } t\in [0,T]. \end{aligned}$$

In particular, the proof of Lemma 3.4 is independent of the nonlocal terms. A similar result for the general pressure law is proven later; see Lemma 5.6.

#### Lemma 3.5

(Higher integrability of the density) Let $(\rho ,u)$ be the smooth solution of (1.9) and (2.10)–(2.13), and let the assumption of Lemma 3.4 hold. Then, for any $K\Subset [b^-(t),b^+(t)]$ for any $t \in [0,T]$, there exists $C(K)>0$ independent of $\varepsilon \in (0,1]$ such that

3.20 $$\begin{aligned} \int _0^T\int _K\rho ^{\gamma +1}(t,x)\,\textrm{d}x \textrm{d}t\leqq C(K). \end{aligned}$$

#### Proof

We divide the proof into two steps.

*Step 1.* For given $K\Subset [b^-(t),b^+(t)]$ for any $t\in [0,T]$, there exist $r_1$ and $r_2$ such that $K\Subset (r_1,r_2)\Subset [b^-(t),b^+(t)]$. Let *w*(*x*) be a smooth function with $\text {supp}\,w\subseteq (r_1,r_2)$ and $w(x)=1$ for $x\in K$. Multiplying (1.9)$_{2}$ by *w*(*x*), we have

3.21 $$\begin{aligned} (\rho u w)_t+\big ((\rho u^2+P(\rho ))w\big )_x&=(\rho u^2+P(\rho ))w_x+\varepsilon (\rho ^{\alpha }w u_x)_x-\varepsilon \rho ^{\alpha }u_xw_x \nonumber \\&\quad \ +\lambda \rho uw+\rho Vw-\rho w \partial _xW*\rho . \end{aligned}$$

Integrating (3.21) over $$[r_1,x)$$ to obtain

3.22 $$\begin{aligned} (\rho u^2+P(\rho ))w&=\varepsilon \rho ^{\alpha }wu_x+\int ^{x}_{r_1}\Big ((\rho u^2+P(\rho ))w_y-\varepsilon \rho ^{\alpha }u_yw_y\Big )\,\textrm{d}y \nonumber \\&\quad -\frac{\textrm{d}}{\textrm{d}t}\int ^x_{r_1}\rho uw\,\textrm{d}y+\int ^x_{r_1}\lambda \rho uw\,\textrm{d}y-\int ^x_{r_1}\rho w \partial _x W*\rho \,\textrm{d}y\nonumber \\&\quad +\int ^x_{r_1}\rho w V\,\textrm{d}y. \end{aligned}$$

Multiplying (3.22) by $\rho w$ and performing a direct calculation, we obtain

3.23 $$\begin{aligned} \rho P(\rho ) w^2&=\varepsilon \rho ^{\alpha +1}w^2u_x-\Big (\rho w\int ^x_{r_1}\rho uw\,\textrm{d}y\Big )_t-\Big (\rho uw\int ^x_{r_1}\rho uw\,\textrm{d}y\Big )_x\nonumber \\&\quad +\rho uw_x\int ^x_{r_1}\rho uw\,\textrm{d}y+\rho w\int ^x_{r_1}\Big ((\rho u^2+P(\rho ))w_y-\varepsilon \rho ^{\alpha }u_yw_y\Big )\,\textrm{d}y\nonumber \\&\quad +\lambda \rho w\int ^x_{r_1}\rho u w\,\textrm{d}y-\rho w\int ^x_{r_1}\rho (\partial _xW*\rho ) w\,\textrm{d} y+\rho w\int ^x_{r_1}\rho V w\,\textrm{d} y\nonumber \\&:=\sum ^{8}_{i=1}K_{i}. \end{aligned}$$

*Step 2.* To estimate $K_i, i=1,\ldots , 8$, in (3.23), we first notice that

$$\begin{aligned} \int _{b^{-}(t)}^{b^+(t)}\rho |u|\, \textrm{d}x\leqq \int _{b^-(t)}^{b^+(t)} (\rho +\rho u^2)\,\textrm{d}x\leqq C. \end{aligned}$$

Then it follows from (3.2) and (3.5) that

$$\begin{aligned} \bigg | \int _0^T\int _{r_1}^{r_2} K_2\, \textrm{d}x\textrm{d}t \bigg |&=\bigg | \int _0^T\int _{r_1}^{r_2} \Big (\rho w\int ^x_{r_1}\rho uw\,\textrm{d}y\Big )_t\, \textrm{d}x\textrm{d}t \bigg |\nonumber \\&\leqq \bigg |\int ^{r_2}_{r_1}\Big (\rho w\int ^x_{r_1}\rho u w\,\textrm{d}y\Big )(T,x)\,\textrm{d}x\bigg |\nonumber \\&\quad +\bigg |\int ^{r_2}_{r_1}\Big (\rho w\int ^x_{r_1}\rho u w\,\textrm{d}y\Big )(0,x)\,\textrm{d}x\bigg |\nonumber \\&\leqq C.\nonumber \end{aligned}$$

Similarly to [13, Lemma 3.5], we obtain

$$\begin{aligned} \bigg |\int _0^T\int _{r_1}^{r_2} K_1\,\textrm{d}x\textrm{d}t \bigg |&\leqq C(r_1,r_2) +\varepsilon \int ^T_0\int ^{r_2}_{r_1}\rho ^{\gamma +1}w^2\,\textrm{d}x\textrm{d}t,\nonumber \\ \bigg | \int _0^T\int _{r_1}^{r_2}K_3\, \textrm{d}x\textrm{d}t \bigg |&=\bigg | \int _0^T\int _{r_1}^{r_2} \Big (\rho uw\int ^x_{r_1}\rho uw\,\textrm{d}y\Big )_x\, \textrm{d}x\textrm{d}t \bigg |=0,\nonumber \\ \bigg | \int _0^T\int _{r_1}^{r_2} K_4\, \textrm{d}x\textrm{d}t \bigg |&=\left| \int _0^T\int _{r_1}^{r_2} \Big (\rho uw_x\int ^x_{r_1}\rho uw\,\textrm{d}y\Big )\, \textrm{d}x\textrm{d}t \right| \leqq C.\nonumber \end{aligned}$$

Using the fact that $\alpha \leqq \gamma ,$ we have

$$\begin{aligned}&\bigg | \int _0^T\int _{r_1}^{r_2} K_5\, \textrm{d}x\textrm{d}t \bigg |\nonumber \\&=\bigg | \int _0^T\int _{r_1}^{r_2} (\rho uw\int ^x_{r_1}\Big ((\rho u^2+P(\rho ))w_y-\varepsilon \rho ^{\alpha }w_yu_y\,\textrm{d}y\Big )\, \textrm{d}x\textrm{d}t \bigg |\nonumber \\&\leqq C +\bigg |\varepsilon \int ^T_0\int ^{r_2}_{r_1}\rho w\int ^x_{r_1}\rho ^{\alpha }w_yu_y\,\textrm{d}x\textrm{d}t\bigg |\nonumber \\&\leqq C(r_1,r_2),\nonumber \end{aligned}$$

where we have used the following estimate

$$\begin{aligned}&\bigg |\varepsilon \int _0^T\int _{r_1}^{r_2} \rho w\int ^x_{r_1}\rho ^{\alpha }w_yu_y\, \textrm{d}y\textrm{d}x\textrm{d}t \bigg |\nonumber \\&\leqq \varepsilon \int ^T_0\int ^{r_2}_{r_1}|\rho ^{\alpha }w_xu_x|\,\textrm{d}x\textrm{d}t\nonumber \\&\leqq \Big (\varepsilon \int ^T_0\int ^{r_2}_{r_1}\rho ^{\alpha }|u_x|^2\,\textrm{d}x \textrm{d}t\Big )^{\frac{1}{2}}\Big (\varepsilon \int ^T_0\int ^{r_2}_{r_1}\rho ^{\alpha }|w_x|^2\,\textrm{d}x\textrm{d}t\Big )^{\frac{1}{2}}\nonumber \\&\leqq C\Big (\varepsilon \int ^T_0\int ^{r_2}_{r_1}\rho ^{\alpha }\,\textrm{d}x\textrm{d}t\Big )^{\frac{1}{2}} \leqq C\Big (\varepsilon \int ^T_0\int ^{r_2}_{r_1}\rho ^{\gamma }\,\textrm{d}x\textrm{d}t\Big )^{\frac{1}{2}}\nonumber \\&\leqq C(r_1,r_2).\nonumber \end{aligned}$$

Moreover, we have

$$\begin{aligned} \bigg |\int _0^T\int _{r_1}^{r_2} K_6\,\textrm{d}x\textrm{d}t \bigg |&\leqq C, \end{aligned}$$

and

$$\begin{aligned}&\int _0^T\int _{r_1}^{r_2} K_7\,\textrm{d}x\textrm{d}t \\&\,=\int _0^T\int _{r_1}^{r_2} \rho (t,x) w(x)\Big (\int ^x_{r_1}\rho (t,y) (\partial _xW*\rho )(t,y) w(y) \,\textrm{d}y\Big ) \textrm{d}x\textrm{d}t \nonumber \\&\,= \int _0^T\int _{r_1}^{r_2} \rho (t,x) w(x) \int ^x_{r_1}\rho (t,y)\Big ( \int ^{b^+(t)}_{b^-(t)}\big (1-2H(x-z)+x-z\big )\rho (t,z)\,\textrm{d}z\Big ) w(y)\,\textrm{d}y\textrm{d}x\textrm{d}t, \nonumber \\&\int _0^T\int _{r_1}^{r_2} K_8\,\textrm{d}x\textrm{d}t\nonumber \\&\, =\int ^T_0\int ^{r_2}_{r_1}\rho (t,x)w(x) \int ^x_{r_1}\rho (t,y)\Big (\int _{b^{-}(t)}^{b^+(t)}\varpi (y-z)\big (u(z)-u(y)\big )\rho (t,z)\,\textrm{d}z\Big ) w(y)\,\textrm{d}y\textrm{d}x\textrm{d}t,\nonumber \end{aligned}$$

which are clearly bounded by *C*. Integrating (3.23) over $[0,T]\times [r_1, r_2],$ and collecting all the estimates in this step, we obtain (3.20). The proof is completed. $\square$

## Inviscid Limit for Polytropic Gases

To prove Theorem 2.3, we intend to apply the compensated compactness framework from Chen-Perepelitsa [17]. For clarity of presentation, we first focus on the polytropic case (1.5), while the general pressure case will be discussed in §5 below.

We first explore some important properties of several special entropy-entropy flux pairs.

### Choice of a special entropy and entropy flux pair

Taking $\psi (s)=\frac{1}{2} {s}{|s|}$ in (2.2), the corresponding entropy and entropy flux are represented as

4.1 $$\begin{aligned} {\left\{ \begin{array}{ll} \displaystyle \eta ^{\#}(\rho ,\rho u)=\frac{1}{2} \rho \int _{-1}^1 (u+\rho ^{\theta } s) |u+\rho ^{\theta }s| [1-s^2]_+^{\mathfrak {b}} \text {d}s,\\[1mm] \displaystyle q^{\#}(\rho , \rho u)=\frac{1}{2} \rho \int _{-1}^1 (u+\theta \rho ^{\theta }s)(u+\rho ^{\theta } s) |u+\rho ^{\theta }s| [1-s^2]_+^{\mathfrak {b}} \text {d}s, \end{array}\right. } \end{aligned}$$

where $\mathfrak {b}=\frac{3-\gamma }{2(\gamma -1)}>-\frac{1}{2}$ and $\theta =\frac{\gamma -1}{2}>0$. A direct calculation shows that

4.2 $$\begin{aligned} |\eta ^{\#}(\rho ,\rho u)|\leqq C_{\gamma } \big (\rho |u|^2+\rho ^{\gamma }\big ), \qquad \,\, q^{\#}(\rho ,\rho u)\geqq C_{\gamma }^{-1} \big (\rho |u|^3+\rho ^{\gamma +\theta }\big ), \end{aligned}$$

where and whereafter $C_\gamma >0$ is a universal constant depending only on $\gamma >1$. We regard $\eta ^{\#}$ as a function of $(\rho ,m)$ to obtain

$$\begin{aligned}&\eta ^{\#}_\rho =\int _{-1}^1 \big (-\frac{1}{2} u+(\theta +\frac{1}{2})\rho ^{\theta } s\big )\,|u+\rho ^{\theta }s| [1-s^2]_+^{\mathfrak {b}} \text {d}s,\qquad \nonumber \\&\eta ^{\#}_m=\int _{-1}^1|u+\rho ^{\theta }s| [1-s^2]_+^{\mathfrak {b}} \text {d}s. \end{aligned}$$

We can directly check that

4.3 $$\begin{aligned} |\eta ^{\#}_m|\leqq C_\gamma \big (|u|+\rho ^\theta \big ),\qquad \,\, |\eta ^{\#}_\rho |\leqq C_\gamma \big (|u|^2+\rho ^{2\theta }\big ). \end{aligned}$$

### Higher integrability of the velocity

The special entropy pair introduced in §4.1 allows us to derive a better estimate for the integrability of the velocity vector field.

The following lemma is important to control the trace estimates for the higher integrability on the velocity; see (4.11). In fact, we have the boundary parts $(u \eta ^{\#})(t,b^\pm (t))$ and $q^{\#}(t,b^\pm (t))$, and it is impossible to have the uniform trace bound (independent of $\varepsilon$) for each of them.

#### Lemma 4.1

([13], Lemma 3.6) For the entropy pair defined in (4.1), the following cancelation property holds:

4.4 $$\begin{aligned} |q^{\#}-u\eta ^{\#}|\leqq C_\gamma \big (\rho ^{\gamma } |u|+ \rho ^{\gamma +\theta } \big ). \end{aligned}$$

#### Lemma 4.2

Let $(\rho ,u)$ be the smooth solution of (1.9) and (2.10)–(2.13), and let the assumption of Lemma 3.4 hold. Then, for any $(r_1,r_2)\Subset [b^-(t), b^+(t)]$, there exists $C(r_1,r_2)>0$ independent of $\varepsilon \in (0,1]$ such that

4.5 $$\begin{aligned} \int _0^T\int _{r_1}^{r_2} \big (\rho |u|^3+\rho ^{\gamma +\theta }\big )(t,x)\, \textrm{d}x \textrm{d}t\leqq C(r_1,r_2). \end{aligned}$$

#### Proof

We divide the proof into four steps.

*Step 1.* Multiplying (1.9)$_{1}$ by $\eta ^{\#}_\rho$ and (1.9)$_{2}$ by $\eta ^{\#}_m$, we have

4.6 $$\begin{aligned}&\eta ^{\#}_t+q^{\#}_x = \eta ^{\#}_m\, \Big (\varepsilon (\rho ^{\alpha } u_x)_x+\lambda \rho u+\rho V-\rho \partial _xW*\rho \Big ). \end{aligned}$$

Using (2.9), a direct calculation shows that

$$\begin{aligned} \frac{\textrm{d}}{\textrm{d}t}\int _x^{b^+(t)}\eta ^{\#}\,\textrm{d}y&=\eta ^{\#}(t,b^+(t)) \frac{\textrm{d}}{\textrm{d}t}b^+(t)+\int _x^{b^+(t)}\eta ^{\#}_t(t,y)\,\textrm{d}y\nonumber \\&= (u\eta ^{\#})(t, b^+(t))+\int _x^{b^+(t)} \eta ^{\#}_t(t,y)\,\textrm{d}y.\nonumber \end{aligned}$$

Integrating (4.6) over $[x,b^+(t))$, we have

4.7 $$\begin{aligned} q^{\#}(t,x)&=\Big ( \int _x^{b^+(t)}\eta ^{\#}(t,y)\,\textrm{d}y\Big )_t+\big (q^{\#}-u\eta ^{\#}\big )(t,b^+(t))\nonumber \\&\quad -\varepsilon \int _x^{b^+(t)} \eta ^{\#}_m(\rho ^{\alpha } u_y)_y\,\textrm{d}y- \lambda \int _x^{b^+(t)} \eta ^{\#}_m\rho u\,\textrm{d}y\nonumber \\&\quad +\int _x^{b^+(t)} \eta ^{\#}_m \rho \partial W*\rho \,\textrm{d}y-\int ^{b^+(t)}_x\eta ^{\#}\rho V\,\textrm{d}y\nonumber \\&=\sum ^6_{i=1}I_i. \end{aligned}$$

We now bound each term of the RHS of (4.7).

*Step 2.* First, for the term involving the trace estimates (the second term) in (4.7), it follows from (4.4) and Lemmas 3.1, 3.3, and 4.1 that

4.8 $$\begin{aligned}&\int _0^T\int ^{r_2}_{r_1} \big |(q^{\#}-u\eta ^{\#})(t,b^+(t))\big |\, \textrm{d}x\textrm{d}t\nonumber \\&\leqq C(r_1,r_2)\int _0^T\big (\rho ^{\gamma +\theta }(t,b^+(t))+(\rho ^{\gamma }|u|)(t, b^+(t))\big )\,\textrm{d}t. \end{aligned}$$

It follows from (3.13) that

4.9 $$\begin{aligned} \int _0^T\big (\rho (t,b^+(t)))^{\gamma +\theta }\textrm{d}t&=\int ^T_0\Big (\rho _0(b)\big (1 +\frac{\kappa (\gamma -\alpha )}{\varepsilon }\rho ^{\gamma -\alpha }_0(b)t\big )^{-\frac{1}{\gamma -\alpha }}\Big )^{\gamma +\theta }\,\textrm{d}t\nonumber \\&\leqq (\rho _0(b))^{\gamma + \theta }T\leqq C. \end{aligned}$$

Similarly to [13, 29], we have

4.10 $$\begin{aligned}&\int _0^T\int ^{r_2}_{r_1} \big (\rho ^{\gamma }|u|\big )(t,b^+(t))\, \textrm{d}x\textrm{d}t\leqq C\varepsilon ^{\frac{p(\gamma -\alpha )-\gamma }{2\gamma }} \leqq C, \end{aligned}$$

where $p>\frac{\gamma }{\gamma -\alpha }$. Substituting (4.9)–(4.10) into (4.8), we obtain

4.11 $$\begin{aligned}&\bigg |\int ^T_0\int ^{r_2}_{r_1}I_2\,\textrm{d}x\textrm{d}t\bigg | =\bigg |\int ^T_0\int ^{r_2}_{r_1}(q^{\#}-u\eta ^{\#})(t,b^+(t))\,\textrm{d}x\textrm{d}t\bigg | \leqq C(r_1,r_2). \end{aligned}$$

For the first term in the RHS of (4.7), using (3.5) and (3.14), we verify that

4.12 $$\begin{aligned}&\bigg |\int ^T_0\int ^{r_2}_{r_1}I_1\,\textrm{d}x\textrm{d}t\bigg |\nonumber \\&=\bigg |\int _0^T\int _{r_1}^{r_2}\Big (\int _x^{b^+(t)}\eta ^{\#}(\rho ,\rho u)\, \textrm{d}y\Big )_t\,\textrm{d}x\textrm{d}t\bigg | \nonumber \\&\leqq \bigg |\int _{r_1}^{r_2}\int _{b^-(t)}^{b^+(t)}\eta ^{\#}(\rho ,\rho u)(T,y)\, \textrm{d}y\textrm{d}x\bigg | +\bigg |\int _{r_1}^{r_2}\int _{b^-(t)}^{b^+(t)}\eta ^{\#}(\rho _0,\rho _0 u_0)\, \textrm{d}y\textrm{d}x\bigg |\nonumber \\&\leqq C(r_1,r_2). \end{aligned}$$

*Step 3.* For $I_3,$ we integrate by parts to obtain

4.13 $$\begin{aligned}&-\varepsilon \int _x^{b^+(t)}\eta ^{\#}_m(\rho ^{\alpha } u_y)_y\,\text {d}y\nonumber \\  &=-\varepsilon \Big (\eta ^{\#}_m(t,b^+(t)) (\rho ^{\alpha } u_x)(t,b^+(t))-\eta ^{\#}_m(t,x)(\rho ^{\alpha } u_x)(t,x) \Big ) \nonumber \\  &\quad \,+\varepsilon \int _x^{b^+(t)}\rho ^{\alpha } u_y(\eta ^{\#}_{mu}u_y+\eta ^{\#}_{m\rho }\rho _y)\,\text {d}y:= J_1+J_2. \end{aligned}$$

First, we estimate $J_2$:

$$\begin{aligned} |J_2|&=\bigg |\varepsilon \int ^{b^+(t)}_x\Big (\eta ^{\#}_{m\rho }\rho ^{\alpha }u_y\rho _y+\eta ^{\#}_{mu}\rho ^{\alpha }u^2_{y}\Big )\,\textrm{d}y\bigg |\nonumber \\&\leqq C\varepsilon \bigg |\int ^{b^+(t)}_x\rho ^{\theta +\alpha -1}u_y\rho _y\,\textrm{d}y\bigg | +\varepsilon \int ^{b^+(t)}_x\rho ^{\alpha }u^2_{y}\,\textrm{d}y\nonumber \\&\leqq \varepsilon \int ^{b^+(t)}_x\rho ^{\gamma +\alpha -3}\rho ^2_y\,\textrm{d}y+\varepsilon \int ^{b^+(t)}_x\rho ^{\alpha }u^2_{y}\,\textrm{d}y, \end{aligned}$$

where we have used the fact that $|\eta ^{\#}_{mu}|\leqq C$ and $|\eta ^{\#}_{m\rho }|\leqq C\rho ^{\theta -1}$. We obtain

4.14 $$\begin{aligned} \int ^T_0\int ^{r_2}_{r_1}|J_2|\,\textrm{d}x\textrm{d}t&\leqq \varepsilon \int ^T_0\int ^{r_2}_{r_1}\int ^{b^+(t)}_x\rho ^{\gamma +\alpha -3}\rho ^2_y\,\textrm{d}y\textrm{d}x\textrm{d}t\nonumber \\&\quad +\varepsilon \int ^T_0\int ^{r_2}_{r_1}\int ^{b^+(t)}_x\rho ^{\alpha }u^2_y\,\textrm{d}y\textrm{d}x\textrm{d}t\nonumber \\&\leqq C(r_1,r_2). \end{aligned}$$

For $J_1,$ we have

4.15 $$\begin{aligned} \varepsilon \bigg |\int ^T_0\int ^{r_2}_{r_1} \eta ^{\#}_m\rho ^{\alpha }u_x\,\textrm{d}x\textrm{d}t\bigg |&\leqq \varepsilon \int ^T_0\int ^{r_2}_{r_1}\rho ^{\alpha }u^2_x\,\textrm{d}x\textrm{d}t+\varepsilon \int ^T_0\int ^{r_2}_{r_1}\rho ^{\alpha }(|u|+\rho ^{\theta })^2\,\textrm{d}x\textrm{d}t\nonumber \\&\leqq C +\varepsilon \int ^T_0\int ^{r_2}_{r_1}\rho ^{2\theta +\alpha }\,\textrm{d}x\textrm{d}t+\varepsilon \int ^T_0\int ^{r_2}_{r_1}\rho ^{\alpha }u^2\,\textrm{d}x\textrm{d}t. \end{aligned}$$

Again, as in [13, 29], to control (4.15), we need that

4.16 $$\begin{aligned} \varepsilon \int ^T_0\int ^{r_2}_{r_1}\rho ^{2\theta +\alpha }\,\textrm{d}x\textrm{d}t&=\varepsilon \int ^T_0\int ^{r_2}_{r_1}\rho ^{\gamma -1+\alpha }\,\textrm{d}x\textrm{d}t \leqq C\int ^T_0\int ^{r_2}_{r_1}\rho ^{\frac{\gamma -1}{2}}\,\textrm{d}x\textrm{d}t\nonumber \\&\leqq C\int ^T_0\int ^{r_2}_{r_1}\rho ^{\gamma }\,\textrm{d}x\textrm{d}t+C(r_1,r_2) \nonumber \\&\leqq C(r_1,r_2). \end{aligned}$$

It follows from (3.5) and (3.14) that, for $\beta =\alpha +\frac{\gamma -1}{2},$

4.17 $$\begin{aligned} \varepsilon \rho ^{\beta }(t,x)&=\varepsilon \Big (\rho ^{\beta }(t,x)-\rho ^{\beta }(t,b^-(t))+\rho ^{\beta }(t,b^-(t))\Big )\nonumber \\&\leqq \varepsilon \beta \int ^{b^+(t)}_{b^-(t)}\rho ^{\beta -1}|\rho _x|\,\textrm{d}x+\varepsilon \rho ^{\beta }_0(b)\nonumber \\&\leqq \beta \Big (\varepsilon ^2\int ^{b^+(t)}_{b^-(t)}\rho ^{2\alpha -3}\rho ^2_x \,\textrm{d}x\Big )^{\frac{1}{2}}\Big (\int ^{b^+(t)}_{b^-(t)}\rho ^{2(\beta -\alpha )+1}\,\textrm{d}x\Big )^{\frac{1}{2}}+\varepsilon \rho ^{\beta }_0(b)\nonumber \\&\leqq C\Big (\int ^{b^+(t)}_{b^-(t)}\rho ^{2(\beta -\alpha )+1}\,\textrm{d}x\Big )^{\frac{1}{2}} +C \leqq C. \end{aligned}$$

Using (4.17), we have

4.18 $$\begin{aligned} \varepsilon \int ^T_0\int ^{r_2}_{r_1}\rho ^{\alpha }u^2\,\textrm{d}x\textrm{d}t&\leqq \Big (\int ^T_0\int ^{r_2}_{r_1}\rho |u|^3\,\textrm{d}x\textrm{d}t\Big )^{\frac{2}{3}}\,\Big (\int ^T_0\int ^{r_2}_{r_1}\varepsilon ^3\rho ^{3\alpha -2}\,\textrm{d}x\textrm{d}t\Big )^{\frac{1}{3}}\nonumber \\&\leqq \Big (\int ^T_0\int ^{r_2}_{r_1}\rho |u|^3\,\textrm{d}x\textrm{d}t\Big )^{\frac{2}{3}}\,\Big (\int ^T_0\int ^{r_2}_{r_1}\varepsilon ^3\rho ^{3\beta }\,\textrm{d}x\textrm{d}t+C(r_1,r_2)\Big )^{\frac{1}{3}}\nonumber \\&\leqq C(r_1,r_2) \Big (\int ^T_0\int ^{r_2}_{r_1}\rho |u|^3\,\textrm{d}x\textrm{d}t\Big )^{\frac{2}{3}}, \end{aligned}$$

where we have used the assumption: $\alpha \geqq \frac{2}{3}.$ Inserting (4.16) and (4.18) into (4.15), we find that, for $\delta >0,$

4.19 $$\begin{aligned}&\varepsilon \bigg |\int ^T_0\int ^{r_2}_{r_1} \eta ^{\#}_m\rho ^{\alpha }u_x\,\textrm{d}x\textrm{d}t\bigg | \leqq C(r_1,r_2) \frac{1}{\delta }+\delta \int ^T_0\int ^{r_2}_{r_1}\rho |u|^3\,\textrm{d}x\textrm{d}t. \end{aligned}$$

Using (2.12) and (4.3), we have

$$ \big |\varepsilon (\eta ^{\#}_m\rho ^{\alpha }u_x)(t,b^+(t))\big | =\kappa \big |(\eta ^{\#}_m\rho ^{\gamma })(t,b^+(t))\big | \leqq C_{\gamma }\big (\rho ^{\gamma }|u|+\rho ^{\gamma +\theta }\big )(t,b^+(t)). $$

Similar again to the argument from [13, 29], we obtain

4.20 $$\begin{aligned} \int ^T_0\int ^{r_2}_{r_1}\big |\varepsilon (\eta ^{\#}_m\rho ^{\alpha }u_x)(t,b^+(t))\big | \,\textrm{d}x\textrm{d}t&\leqq C(r_1,r_2) \int ^T_0\big (\rho ^{\gamma }|u|+\rho ^{\gamma +\theta }\big )(t,b^+(t))\,\textrm{d}t\nonumber \\&\leqq C(r_1,r_2). \end{aligned}$$

Combining (4.19) with (4.20) yields

4.21 $$\begin{aligned} \bigg |\int ^T_0\int ^{r_2}_{r_1}J_1\,\textrm{d}x\textrm{d}t\bigg |&\leqq \delta \int ^T_0\int ^{r_2}_{r_1}\rho |u|^3\,\textrm{d}x\textrm{d}t +C(r_1,r_2). \end{aligned}$$

Inserting (4.21) and (4.14) into (4.13), we obtain

4.22 $$\begin{aligned} \bigg |\int ^T_0\int ^{r_2}_{r_1}I_3\,\textrm{d}x\textrm{d}t\bigg |&\leqq \delta \int ^T_0\int ^{r_2}_{r_1}\rho |u|^3\,\textrm{d}x\textrm{d}t +C(r_1,r_2). \end{aligned}$$

*Step 4.* We also obtain the estimates for $I_4$, $I_5$, and $I_6$:

$$\begin{aligned} \bigg |\int ^T_0\int ^{r_2}_{r_1}I_4\,\textrm{d}x\textrm{d}t\bigg |&=\bigg |\int ^T_0\int ^{r_2}_{r_1}\int ^{b^+(t)}_x\rho |u|(|u|+\rho ^{\theta }) \,\textrm{d}y\textrm{d}x\textrm{d}t\bigg |\nonumber \\&=\bigg |\int ^T_0\int ^{r_2}_{r_1}\rho u^2\,\textrm{d}x\textrm{d}t\bigg | +\bigg |\int ^T_0\int ^{r_2}_{r_1}\rho ^{\gamma }\,\textrm{d}x\textrm{d}t\bigg | \leqq C(r_1,r_2), \end{aligned}$$

$$\begin{aligned}&\bigg |\int ^T_0\int ^{r_2}_{r_1}I_5\,\text {d}x\text {d}t\bigg |\nonumber \\  &=\bigg |\int ^T_0\int ^{r_2}_{r_1}\int ^{b^+(t)}_x\rho \eta ^{\#}_m\partial _xW*\rho \,\text {d}y\text {d}x\text {d}t\bigg |\\  &=\bigg |\int ^T_0\int ^{r_2}_{r_1}\int ^{b^+(t)}_x\rho \eta ^{\#}_m\Big (M-2\int ^{y}_{b^-(t)}\rho (t,z)\,\text {d}z+yM -\int ^{b^+(t)}_{b^{-}(t)}z\rho (t,z) \,\text {d}z\Big )\,\text {d}y\text {d}x\text {d}t\bigg | \\&\leqq C(r_1,r_2), \end{aligned}$$

4.23 $$\begin{aligned}&\bigg |\int ^T_0\int ^{r_2}_{r_1}I_6\,\text {d}x\text {d}t\bigg |\nonumber \\  &=\bigg |\int ^T_0\int ^{r_2}_{r_1}\int ^{b^+(t)}_x\eta ^{\#}_m\rho V\,\text {d}y\,\text {d}x\text {d}t\bigg |\nonumber \\  &=\bigg |\int ^T_0\int ^{r_2}_{r_1}\int ^{b^+(t)}_x\eta ^{\#}_m\rho \Big (\int ^{b^+(t)}_{b^-(t)}\varpi (y-z)(u(y)-u(z)) \rho (t,z)\,\text {d}z\Big )\,\text {d}y\,\text {d}x\text {d}t\bigg |\nonumber \\&\leqq C(r_1,r_2). \end{aligned}$$

Combining estimates (4.11)–(4.12) with estimates (4.22)–(4.23), we conclude (4.5) from (4.2) and (4.7). $\square$

### $H_\textrm{loc}^{-1}(\mathbb {R}_+^2)$–Compactness

In this section we use the uniform estimates obtained in §4.2 to prove the following key lemma, which states the $H_\textrm{loc}^{-1}(\mathbb {R}_+^2)-$compactness of the dissipation measures for the approximate solutions.

#### Lemma 4.3

Let $(\eta ^{\psi },q^{\psi })$ be the weak entropy pair generated by any $\psi \in C_0^2(\mathbb {R})$, defined in (2.2), and let $\alpha \in [\frac{2}{3}, \gamma ]$. Then, for the solution sequence $(\rho ^{\varepsilon },u^{\varepsilon })$ with $m^{\varepsilon }=\rho ^{\varepsilon }u^{\varepsilon }$ of CNSEs (1.9) and (2.10)–(2.13), the following sequence

$$\begin{aligned} \eta ^{\psi }(\rho ^{\varepsilon },m^{\varepsilon })_t+q^{\psi }(\rho ^{\varepsilon },m^{\varepsilon })_x \qquad \text{ is } \text{ compact } \text{ in } H_\textrm{loc}^{-1}(\mathbb {R}_+\times \mathbb {R} ). \end{aligned}$$

#### Proof

To prove this lemma, we first recall the following results for the entropy pair $(\eta ^{\psi },q^{\psi })$ generated by $\psi \in C_0^2(\mathbb {R})$; see also [17, 18] for details.

For a $C^2$–function $\psi :\mathbb {R}\rightarrow \mathbb {R}$, compactly supported on the interval [*a*, *b*], we have

$$\begin{aligned} \textrm{supp}(\eta ^{\psi }),\,\textrm{supp}(q^{\psi })\subset \left\{ {(\rho ,m)=(\rho ,\rho u)\,:\, u+\rho ^{\theta }\geqq a,\quad u-\rho ^{\theta }\leqq b}\right\} . \end{aligned}$$

Furthermore, from [17, Lemma 2.1], there exists $C_{\psi }>0$ such that, for any $\rho \geqq 0$ and $u\in \mathbb {R}$, we have the following facts:

(i) For $$\gamma \in (1,3]$$,
  4.24 $$\begin{aligned} |\eta ^{\psi }(\rho ,m)|+|q^{\psi }(\rho ,m)|\leqq C_{\psi }\rho . \end{aligned}$$
(ii) For $$\gamma \in (3,\infty )$$,
  4.25 $$\begin{aligned} |\eta ^{\psi }(\rho ,m)|\leqq C_{\psi }\rho ,\quad \ |q^{\psi }(\rho ,m)|\leqq C_{\psi }(\rho +\rho ^{1+\theta }). \end{aligned}$$
(iii) If $\eta ^{\psi }$ is considered as a function of $(\rho ,m)$, $m=\rho u$, then
  4.26 $$\begin{aligned} |\eta ^{\psi }_m(\rho ,m)|\leqq C_{\psi },\quad \, |\eta ^{\psi }_{\rho }(\rho ,m)|\leqq C_{\psi }(1+\rho ^{\theta }), \end{aligned}$$
  and, if $\eta ^{\psi }_m$ is considered as a function of $(\rho , u)$, then
  4.27 $$\begin{aligned} |\eta ^{\psi }_{mu}(\rho ,\rho u)|+|\rho ^{1-\theta }\eta ^{\psi }_{m\rho }(\rho ,\rho u)|\leqq C_{\psi }. \end{aligned}$$

Now we are going to prove Lemma 4.3.

A direct computation on (1.9)$_{1}$ $\times \eta ^{\psi }_{\rho }(\rho ^{\varepsilon },m^{\varepsilon }) +(1.9)_2\times \eta ^{\psi }_m(\rho ^{\varepsilon },m^{\varepsilon })$ gives

$$\begin{aligned} \displaystyle \eta ^{\psi }(\rho ^{\varepsilon },m^{\varepsilon })_t+q^{\psi }(\rho ^{\varepsilon },m^{\varepsilon })_x =&\,\varepsilon \big (\eta ^{\psi }_m(\rho ^{\varepsilon },m^{\varepsilon })(\rho ^{\varepsilon })^{\alpha }u_x^{\varepsilon }\big )_x -\varepsilon \eta ^{\psi }_{mu}(\rho ^{\varepsilon },m^{\varepsilon })(\rho ^{\varepsilon })^{\alpha }(u_x^{\varepsilon })^2\nonumber \\&\,-\varepsilon \eta ^{\psi }_{m\rho }(\rho ^{\varepsilon },m^{\varepsilon })(\rho ^{\varepsilon })^{\alpha }\rho _x^{\varepsilon }u_x^{\varepsilon } \nonumber \\&+\eta ^{\psi }_m\big (\lambda \rho ^{\varepsilon }u^{\varepsilon }+\rho ^{\varepsilon }V-\rho ^{\varepsilon }\partial _xW*\rho ^{\varepsilon }\big ).\nonumber \end{aligned}$$

For any compact set $K\Subset [b^-(t),b^+(t)]$, using (4.27) and the Cauchy-Schwartz inequality, we have

$$\begin{aligned} \begin{aligned} \displaystyle \int _{0}^{T}\!\!\!\int _{K}&\big |\eta ^{\psi }_{mu}(\rho ^{\varepsilon },m^{\varepsilon })(\rho ^{\varepsilon })^{\alpha } (u_x^{\varepsilon })^2 +\eta ^{\psi }_{m\rho }(\rho ^{\varepsilon },m^{\varepsilon })(\rho ^{\varepsilon })^{\alpha }\rho _x^{\varepsilon }u_x^{\varepsilon }\big |\,\textrm{d}x\textrm{d}t\\&\displaystyle \leqq C_{\psi }\int _{0}^{T}\!\!\!\int _{K}(\rho ^{\varepsilon })^{\alpha }(u_x^{\varepsilon })^2\, \textrm{d}x\textrm{d}t +C_{\psi }\int _{0}^{T}\!\!\!\int _{K}(\rho ^{\varepsilon })^{\alpha +\gamma -3}(\rho _x^{\varepsilon })^2\, \textrm{d}x\textrm{d}t \leqq C, \\ \displaystyle \int _{0}^{T\!\!\!}\int _{K}&\big |\eta ^{\psi }_m \big (\lambda \rho ^{\varepsilon }u^{\varepsilon }+\rho ^{\varepsilon } V-\rho ^{\varepsilon }\partial _xW*\rho ^{\varepsilon }\big )\big |\,\textrm{d}x\textrm{d}t\\&\displaystyle \leqq C_{\psi }\int _{0}^{T}\!\!\!\int _{K}\rho ^{\varepsilon }(u^{\varepsilon })^2+\rho ^{\varepsilon } \,\textrm{d}x\textrm{d}t +C_{\psi }\int _{0}^{T}\!\!\!\int _{K}|\rho ^{\varepsilon }\partial _xW*\rho ^{\varepsilon }| \,\textrm{d}x\textrm{d}t \leqq C(K). \end{aligned} \end{aligned}$$

This implies that

4.28 $$\begin{aligned}&-\varepsilon \eta ^{\psi }_{mu}(\rho ^{\varepsilon },m^{\varepsilon })(\rho ^{\varepsilon })^{\alpha }(u_x^{\varepsilon })^2 -\varepsilon \eta ^{\psi }_{m\rho }(\rho ^{\varepsilon },m^{\varepsilon })(\rho ^{\varepsilon })^{\alpha }\rho _x^{\varepsilon }u_x^{\varepsilon }\nonumber \\&+\eta ^{\psi }_m(\lambda \rho ^{\varepsilon }u^{\varepsilon }+\rho ^{\varepsilon }V-\rho ^{\varepsilon }\partial _xW*\rho ^{\varepsilon })\nonumber \\&\,\,\text{ is } \text{ uniformly } \text{ bounded } \text{ in } L^1([0,T]\times K), \end{aligned}$$

so that it is compact in $W_\textrm{loc}^{-1,p_1}(\mathbb {R}_+^2)$ for $1<p_1<2$.

If $$2\alpha \leqq \gamma +1,$$ then

4.29 $$\begin{aligned} \varepsilon ^{\frac{4}{3}}\int ^T_0\int _{K}(\rho ^{\varepsilon })^{2\alpha }\,\textrm{d}x\textrm{d}t\leqq C(K) \varepsilon ^{\frac{4}{3}}. \end{aligned}$$

Following [29], we see that, if $2\alpha \ge \gamma +1$ and $\alpha <\gamma$, estimate (4.17) implies that

4.30 $$\begin{aligned} \varepsilon ^{\frac{4}{3}}\int ^T_0\int _{K}(\rho ^{\varepsilon })^{2\alpha }\,\textrm{d}x\textrm{d}t&\leqq C(K) \varepsilon ^{\frac{1}{3}}\int ^T_0\int _K(\rho ^{\varepsilon })^{\alpha -\frac{\gamma -1}{2}}\,\textrm{d}x\textrm{d}t\nonumber \\&\quad +C(K )\varepsilon ^{\frac{1}{3}}\int ^T_0\int _K(\rho ^{\varepsilon })^{\gamma +1}\,\textrm{d}x\textrm{d}t. \end{aligned}$$

It follows from (3.20), (4.26), and (4.29)–(4.30) that

$$\begin{aligned} \begin{aligned} \displaystyle \int _{0}^{T}\int _{K}\Big (\varepsilon \eta ^{\psi }_m(\rho ^{\varepsilon },m^{\varepsilon }) (\rho ^{\varepsilon })^{\alpha }u_x^{\varepsilon }\Big )^{\frac{4}{3}}\, \textrm{d}x\textrm{d}t&\displaystyle \leqq \int _{0}^{T}\int _{K}\varepsilon ^{\frac{4}{3}} (\rho ^{\varepsilon })^{^{\frac{4\alpha }{3}}}|u_x^{\varepsilon }|^{\frac{4}{3}}\,\textrm{d}x\textrm{d}t\\&\displaystyle \leqq C\varepsilon ^{\frac{4}{3}}\int _{0}^{T}\int _{K} (\rho ^{\varepsilon })^{\alpha }|u_x^{\varepsilon }|^2\,\textrm{d}x\textrm{d}t\\&\quad +C\varepsilon ^{\frac{4}{3}}\int _{0}^{T}\int _{K} (\rho ^{\varepsilon })^{2\alpha }\,\textrm{d}x\textrm{d}t\\&\displaystyle \leqq C(K)\varepsilon ^{\frac{1}{3}}+C\varepsilon ^{\frac{4}{3}}\int _{0}^{T}\int _{K} (\rho ^{\varepsilon })^{\gamma +1}\,\textrm{d}x\textrm{d}t\\&\displaystyle \leqq C(K)\varepsilon ^{\frac{1}{3}}\rightarrow 0\qquad \ \text {as }\varepsilon \rightarrow 0^+. \end{aligned} \end{aligned}$$

Then (4.3) and (4.28) yield

4.31 $$\begin{aligned} \eta ^{\psi }(\rho ^{\varepsilon },m^{\varepsilon })_t+q^{\psi }(\rho ^{\varepsilon },m^{\varepsilon })_x \quad \text{ is } \text{ compact } \text{ in } W_\textrm{loc}^{-1,p_2}(\mathbb {R}_+^2) \ \ \text{ for } \text{ some } 1<p_2<2. \end{aligned}$$

Furthermore, for $\gamma \in (1,3],$ using (4.24) and Lemma 3.5, we have

$$\begin{aligned} \int ^T_0\int _K\big (|\eta ^{\psi }(\rho ^{\varepsilon },m^{\varepsilon })|+|q^{\psi }(\rho ^{\varepsilon },m^{\varepsilon })|\big )^{\gamma +1}\,\textrm{d}x\textrm{d}t&\leqq C\int ^T_0\int _K(\rho ^{\varepsilon })^{\gamma +1}\,\textrm{d}x\textrm{d}t \\&\leqq C(K). \end{aligned}$$

For $\gamma \in (3, \infty ),$ using (4.25) and Lemma 4.2, we have

$$\begin{aligned}&\int ^T_0\!\!\!\int _K\big (|\eta ^{\psi }(\rho ^{\varepsilon },m^{\varepsilon })| +|q^{\psi }(\rho ^{\varepsilon },m^{\varepsilon })|\big )^{\frac{\gamma +\theta }{\theta +1}}\,\textrm{d}x\textrm{d}t\nonumber \\&\leqq C\int ^T_0\!\!\!\int _K\big (|\rho ^{\varepsilon }|^{\frac{\gamma +\theta }{\theta +1}} +|\rho ^{\varepsilon }|^{\gamma +\theta }\big )\,\textrm{d}x\textrm{d}t\leqq C(K). \nonumber \end{aligned}$$

Thus, using the last two estimates, we obtain

$$\begin{aligned} (\eta ^{\psi }(\rho ^{\varepsilon },m^{\varepsilon }),\, q^{\psi }(\rho ^{\varepsilon },m^{\varepsilon })) \qquad \, \text{ is } \text{ uniformly } \text{ bounded } \text{ in } L_\textrm{loc}^{p_3}(\mathbb {R}_+^2) \ \ \text{ for }\ p_3>2, \end{aligned}$$

where $p_3=\gamma +1>2$ when $\gamma \in (1,3]$, and $p_3=\frac{\gamma +\theta }{1+\theta }>2$ when $\gamma \in (3,\infty )$. This implies that

4.32 $$\begin{aligned} \eta ^{\psi }(\rho ^{\varepsilon },m^{\varepsilon })_t+q^{\psi }(\rho ^{\varepsilon },m^{\varepsilon })_x \quad \, \text{ is } \text{ uniformly } \text{ bounded } \text{ in } W_\textrm{loc}^{-1, p_3}(\mathbb {R}_+^2) \text{ for } p_3>2. \end{aligned}$$

Then, using (4.31)–(4.32) and the interpolation compactness theorem (*cf*. [25, 26]), we conclude Lemma 4.3. $\square$

### Proof of Theorem 2.3 for the polytropic equation of state

Recall the following compactness theorem established by Chen-Perepelitsa in [17]:

#### Theorem 4.4

(Chen-Perepelitsa [17]) Let $(\eta ^{\psi },q^{\psi })$ be a weak entropy pair generated by $\psi \in C_0^2(\mathbb {R})$. Assume that the sequence $(\rho ^{\varepsilon },u^{\varepsilon })(t,x)$ defined on $\mathbb {R}_+\times \mathbb {R}$ with $m^{\varepsilon }=\rho ^{\varepsilon }u^{\varepsilon }$ satisfies the following conditions:

(i) For any $-\infty<r_1<r_2<\infty$ and $T>0$,
  $$\begin{aligned} \int _{0}^{T}\int _{r_1}^{r_2}(\rho ^\varepsilon )^{\gamma +1}\,\textrm{d}x\textrm{d}t\leqq C(r_1,r_2), \end{aligned}$$
  where $C>0$ is independent of $\varepsilon$.
(ii) For any set $K\Subset \mathbb {R}$,
  $$\begin{aligned} \int _{0}^{T}\int _{K}\big ((\rho ^\varepsilon )^{\gamma +\theta }+\rho ^\varepsilon |u^\varepsilon |^3\big )\,\textrm{d}x\textrm{d}t\leqq C(K), \end{aligned}$$
  where $C(K)>0$ is independent of $\varepsilon$.
(iii) The sequence of entropy dissipation measures
  $$\begin{aligned} \eta ^{\psi }(\rho ^{\varepsilon },m^{\varepsilon }) _t+q^{\psi }(\rho ^{\varepsilon },m^{\varepsilon })_x \qquad \, \text{ is } \text{ compact } \text{ in } H_\textrm{loc}^{-1}(\mathbb {R}_+^2). \end{aligned}$$

Then there exist both a subsequence (still denoted) $(\rho ^{\varepsilon },m^{\varepsilon })(t,x)$ and a vector-valued function $(\rho , m)(t,x)$ such that

$$\begin{aligned} (\rho ^{\varepsilon },m^{\varepsilon })\rightarrow (\rho , m)\qquad \ a.e. \text { as }\varepsilon \rightarrow 0^+. \end{aligned}$$

The uniform estimates and compactness properties obtained in §3–§4, along with Theorem 4.4, imply that, for the sequence of solutions $(\rho ^\varepsilon , m^\varepsilon )$ satisfying (1.9) and (2.10)–(2.13), there exist both a subsequence (still denoted) $(\rho ^{\varepsilon },m^{\varepsilon })(t,x)$ and a vector-valued function $(\rho , m)(t,x)$ such that

$$\begin{aligned} (\rho ^{\varepsilon },m^{\varepsilon })\rightarrow (\rho , m)\qquad \ a.e.\,\, (t,x)\in \mathbb {R}_+\times \mathbb {R}\quad \text {as }\varepsilon \rightarrow 0^+. \end{aligned}$$

Notice that

$$ |m|^{\frac{3(\gamma +1)}{\gamma +3}}\leqq C\big (\rho |u|^3+\rho ^{\gamma +1}\big ). $$

Then, using Lemmas 4.2 and 3.5, we obtain

$$\begin{aligned} (\rho ^{\varepsilon },m^{\varepsilon })\rightarrow (\rho ,m) \qquad \text { in } L^{q_1}_\textrm{loc}(\mathbb {R}_+\times \mathbb {R})\times L^{q_2}_\textrm{loc}(\mathbb {R}_+\times \mathbb {R}), \end{aligned}$$

for $$q_1\in [1,\gamma +1)$$ and $$q_2\in [1,\frac{3(\gamma +1)}{\gamma +3}).$$

Using again Lemmas 4.2 and 3.5, we have

4.33 $$\begin{aligned} \eta ^{*}(\rho ^{\varepsilon },m^{\varepsilon })\rightarrow \eta ^{*}(\rho ,m)\qquad \text { in }L^1_\textrm{loc}(\mathbb {R}_+\times \mathbb {R})\quad \text { as }\varepsilon \rightarrow 0^+. \end{aligned}$$

Since $\eta ^{*}$ is a positive convex function, we use (3.2), (3.5), (4.33), and Fatou’s lemma to see that, for all $t_2\geqq t_1\geqq 0,$

$$\begin{aligned}&\int ^{t_2}_{t_1}\int _{\mathbb {R}}\big (\eta ^{*}(\rho ,m)+\frac{1}{2}\rho (W+\frac{1}{2})*\rho \big )(t,x)\,\textrm{d}x \textrm{d}t\nonumber \\&\leqq (t_2-t_1)\int _{\mathbb {R}}\big (\eta ^{*}(\rho _0,m_0)+\frac{1}{2}\rho _0 (W+\frac{1}{2})*\rho _0\big )\,(x)\,\textrm{d}x. \end{aligned}$$

Then, by the Lebesgue point theorem, we obtain

$$\begin{aligned} \int _{\mathbb {R}}\big (\eta ^{*}(\rho ,m)+\frac{1}{2}\rho W*\rho \big )(t,x) \,\textrm{d}x&\leqq \int _{\mathbb {R}}\big (\eta ^{*}(\rho _0,m_0)+\frac{1}{2}\rho _0 W*\rho _0\big )(x)\,\textrm{d}x \\&:=\mathcal {E}_0. \end{aligned}$$

We now prove that $(\rho ,m)$ is an entropy solution of the Cauchy problem (1.1) and (1.5)–(1.6) for the polytropic case.

Let $\Psi (t,x)\in C^1(\mathbb {R}_+\times \mathbb {R})$ be a function with compact support and supp$\,\Psi (t,\cdot )\Subset (b^-(t),b^+(t))$ for any $t\in [0,T]$. Then

$$ \int _{\mathbb {R}^2_+}\big (\rho ^{\varepsilon }\Psi _t+\rho ^{\varepsilon }u^{\varepsilon }\Psi _x\big )\,\textrm{d}x \textrm{d}t+\int _{\mathbb {R}}\rho ^{\varepsilon }_0(x)\Psi (0,x)\,\textrm{d}x=0. $$

By the Lebesgue dominated convergence theorem, through taking limit $\varepsilon \rightarrow 0^+,$ up to a subsequence, we have

$$ \int _{\mathbb {R}^2_+}\big (\rho \Psi _t+\rho u\Psi _x\big )\,\textrm{d}x\textrm{d}t+\int _{\mathbb {R}}\rho _0(x)\Psi (0,x)\,\textrm{d}x=0. $$

Next, we consider the momentum equation. First, we have

4.34 $$\begin{aligned}&\int _{\mathbb {R}^2_+}\Big (\rho ^{\varepsilon }u^{\varepsilon }\Psi _t+\big (\rho ^{\varepsilon }(u^{\varepsilon })^2+P(\rho ^{\varepsilon })\big )\Psi _x +\rho ^{\varepsilon }\big (\lambda u^{\varepsilon }+V +\partial _xW*\rho ^{\varepsilon }\big )\Psi \Big )\,\textrm{d}x\textrm{d}t\nonumber \\&\quad +\int _{\mathbb {R}}(\rho ^{\varepsilon }_0 u^{\varepsilon }_0)(x)\Psi (0,x)\,\textrm{d}x=\varepsilon \int _{\mathbb {R}^2_+}(\rho ^{\varepsilon })^{\alpha }u^{\varepsilon }_x\Psi _x\,\textrm{d}x\textrm{d}t. \end{aligned}$$

Since

$$\begin{aligned} \varepsilon \bigg |\int _{\mathbb {R}^2_+}(\rho ^{\varepsilon })^{\alpha }u^{\varepsilon }_x\Psi _x\,\textrm{d}x\textrm{d}t\bigg |&\leqq C\sqrt{\varepsilon }\Big (\int ^T_0\int _K\varepsilon (\rho ^{\varepsilon })^{\alpha }(u^{\varepsilon }_x)^2\,\textrm{d}x\textrm{d}t\Big )^{\frac{1}{2}} \Big (\int ^T_0\int _K(\rho ^{\varepsilon })^{\alpha }\,\textrm{d}x\textrm{d}t\Big )^{\frac{1}{2}}\nonumber \\&\leqq C(K) \sqrt{\varepsilon }\rightarrow 0 \qquad \text { as }\varepsilon \rightarrow 0^+, \end{aligned}$$

then it follows from (4.34) that

$$\begin{aligned}&\int _{\mathbb {R}^2_+}\Big (m\Psi _t+\big (\frac{m^2}{\rho }+P(\rho )\big )\Psi _x\Big )\,\textrm{d}x\textrm{d}t +\int _{\mathbb {R}}m_0(x)\Psi (0,x)\,\textrm{d}x\nonumber \\&\qquad =\int _{\mathbb {R}^2_+}(-\lambda m-\rho V +\rho \partial _xW*\rho )\Psi \,\textrm{d}x\textrm{d}t.\nonumber \end{aligned}$$

The verification of the entropy inequality is direct. Therefore, the proof of Theorem 2.3, and hence Theorem 2.2 for the polytropic case, is completed. $\square$

## Proof of Theorem 2.3 for the General Pressure Law

This section is dedicated to the essential improvements of the arguments in §3–§4 necessary to prove Theorem 2.3 for the general pressure case.

### Properties of the general pressure law and the related internal energy

In this section, we present some useful estimates involving the general pressure $P(\rho )$ with (1.2)–(1.4) and the corresponding internal energy $e(\rho ).$

Denote

5.1 $$\begin{aligned} k(\rho ):=\int _{0}^{\rho }\frac{\sqrt{P'(y)}}{y}\,\textrm{d} y. \end{aligned}$$

By direct calculation, we recall the following asymptotic behaviors of $P(\rho )$, $e(\rho )$, and $k(\rho )$.

#### Lemma 5.1

([14], Lemma 3.1) The constant $\rho _{*}$ in (1.3) can be chosen small enough, and the constant $\rho ^{*}$ in (1.4) large enough, so that the following estimates hold:

(i) When $$\rho \in (0,\rho _{*}]$$,
  5.2 $$\begin{aligned} \left\{ \begin{aligned}&\underline{\kappa }_{1}\rho ^{\gamma _1}\leqq P(\rho )\leqq \bar{\kappa }_{1}\rho ^{\gamma _1},\\&\underline{\kappa }_{1}\gamma _1\rho ^{\gamma _1-1}\leqq P'(\rho )\leqq \bar{\kappa }_{1}\gamma _1\rho ^{\gamma _1-1},\\&\underline{\kappa }_{1}\gamma _1(\gamma _1-1)\rho ^{\gamma _1-2}\leqq P''(\rho )\leqq \bar{\kappa }_{1}\gamma _1(\gamma _1-1)\rho ^{\gamma _1-2}, \end{aligned} \right. \end{aligned}$$
  and, when $$\rho \in [\rho ^{*},\infty )$$,
  5.3 $$\begin{aligned} \left\{ \begin{aligned}&\underline{\kappa }_{2}\rho ^{\gamma _2}\leqq P(\rho )\leqq \bar{\kappa }_{2}\rho ^{\gamma _2},\\&\underline{\kappa }_{2}\gamma _2\rho ^{\gamma _2-1}\leqq P'(\rho )\leqq \bar{\kappa }_{2}\gamma _2\rho ^{\gamma _2-1},\\&\underline{\kappa }_{2}\gamma _2(\gamma _2-1)\rho ^{\gamma _2-2}\leqq P''(\rho )\leqq \bar{\kappa }_{2}\gamma _2(\gamma _2-1)\rho ^{\gamma _2-2}, \end{aligned} \right. \end{aligned}$$
  where we have denoted $\underline{\kappa }_{i}:=(1-\mathfrak {a}_0)\kappa _{i}$ and $\bar{\kappa }_{i}:=(1+\mathfrak {a}_0) \kappa _{i}$ with $\mathfrak {a}_0=\frac{3-\gamma _1}{2(\gamma _1+1)}$ and $i=1,2$.
(ii) For $e(\rho )$ and $k(\rho )$, there exists $C>0$ depending on $(\gamma _1, \gamma _2, \kappa _1,\kappa _2, \rho _{*}, \rho ^{*})$ such that
  5.4 $$\begin{aligned}&C^{-1}\rho ^{\gamma _1-1}\leqq e(\rho )\leqq C\rho ^{\gamma _1-1},\quad C^{-1}\rho ^{\gamma _1-2}\leqq e'(\rho )\leqq C\rho ^{\gamma _1-2} \quad \,\, \text { for }\rho \in (0,\rho _{*}],\nonumber \\&C^{-1}\rho ^{\gamma _2-1}\leqq e(\rho )\leqq C\rho ^{\gamma _2-1},\quad C^{-1}\rho ^{\gamma _2-2}\leqq e'(\rho )\leqq C\rho ^{\gamma _2-2} \quad \,\, \text { for }\rho \in [\rho ^{*},\infty ), \end{aligned}$$
  and, for $$i=0,1$$,
  $$\begin{aligned}&\qquad C^{-1}\rho ^{\theta _{1}-i}\leqq k^{(i)}(\rho )\leqq C\rho ^{\theta _{1}-i},\,\, C^{-1}\rho ^{\theta _{1}-2}\leqq |k''(\rho )|\leqq C\rho ^{\theta _1-2}\,\,\,\, \text {for } \rho \in (0,\rho _{*}],\\&\qquad C^{-1}\rho ^{\theta _{2}-i}\leqq k^{(i)}(\rho )\leqq C\rho ^{\theta _{2}-i}, \,\, C^{-1}\rho ^{\theta _{2}-2}\leqq |k''(\rho )|\leqq C\rho ^{\theta _2-2} \,\,\,\,\text {for } \rho \in [\rho ^{*},\infty ), \end{aligned}$$
  where $$\theta _{1}=\frac{\gamma _1-1}{2}$$ and $$\theta _2=\frac{\gamma _2-1}{2}$$.

### Uniform estimates of the approximate solutions

We start with the basic energy estimate.

#### Lemma 5.2

(Basic energy estimate) For the smooth solution $(\rho , u)(t,x)$ of problem (1.9) and (2.10)–(2.13), the following estimate holds:

5.5 $$\begin{aligned}&\int ^{b^+(t)}_{b^-(t)}\Big (\frac{1}{2}\rho u^2+\rho e(\rho )+\frac{1}{2}\rho W*\rho \Big )(t,x)\,\textrm{d}x +\int _0^t\int _{b^-(s)}^{b^+(s)} (\varepsilon \rho ^{\alpha }|u_x|^2-\lambda \rho u^2)(s,x)\,\textrm{d}x\textrm{d}s\nonumber \\&+\frac{1}{2}\int ^t_0\int ^{b^+(s)}_{b^-(s)}\int ^{b^+(s)}_{b^-(s)}\varpi (x-y)\rho (x)\rho (y)|u(y)-u(x)|^2\,\textrm{d}y\textrm{d}x\textrm{d}s = \mathcal {E}^{\varepsilon }_0\le C_0. \end{aligned}$$

The proof is almost the same as for Lemma 3.1, with (3.6) replaced by

$$\begin{aligned} -P(\rho )u_{\xi }=\frac{P(\rho )}{\rho ^2}\rho _{\tau }=e'(\rho )\rho _{\tau }=e(\rho )_{\tau }. \end{aligned}$$

Using Lemma 5.2, we obtain

#### Corollary 5.3

It follows from (5.4) and Lemma 5.2 that

$$\begin{aligned} \int _{b^-(t)}^{b^+(t)}\rho ^{\gamma _2}(t,x)\,\textrm{d}x \leqq C \int _{b^-(t)}^{b^+(t)}\big (\rho +\rho e(\rho )\big )(t,x)\,\textrm{d}x\leqq C \qquad \text { for }t\geqq 0. \end{aligned}$$

#### Lemma 5.4

(Higher moment estimate) The following estimate holds:

$$\begin{aligned} \int ^{b^+(t)}_{b^-(t)}x^2\rho (t,x) \,\textrm{d}x\leqq C. \end{aligned}$$

#### Proof

Following the same argument as in Lemma 3.2, it suffices to show that

$$\begin{aligned}&\bigg |\int ^T_0\int ^{b^+(s)}_{b^-(s)}P(\rho )(s,x)\,\textrm{d}x\textrm{d}s\bigg |\\&\leqq \bigg |\int ^T_0\int ^{b^+(s)}_{b^-(s)}\big (P(\rho )-\rho e(\rho )\big )(s,x)\,\textrm{d}x\textrm{d}s\bigg | +\bigg |\int ^T_0\int ^{b^+(s)}_{b^-(s)}\big (\rho e(\rho )\big )(s,x)\,\textrm{d}x\textrm{d}s\bigg |\\&\leqq \bigg |\int ^T_0\int ^{b^+(s)}_{b^-(s)}\big (\rho ^2 e'(\rho )-\rho e(\rho )\big )(s,x)\,\textrm{d}x\textrm{d}s\bigg |+C \leqq C. \end{aligned}$$

$$\square $$

For later use, we analyze the behavior of density $\rho$ on the free boundary. It follows from (3.3)$_{1}$ and (3.4) that

$$\begin{aligned} \rho _{\tau }(\tau ,M)=-\frac{1}{\varepsilon }\big (\frac{\rho P}{\mu (\rho )}\big )(\tau ,M)\leqq 0. \end{aligned}$$

This yields that $\rho (\tau , M)\leqq \rho _{0}(M)$.

In the Eulerian coordinates, it is equivalent to

5.6 $$\begin{aligned} \rho (t,b^+(t))\leqq \rho _0(b). \end{aligned}$$

Moreover, noting that $\rho ^{\gamma _1}_0(\pm b)b\leqq C_0$ and $b\geqq (\rho _{*})^{-\gamma _1}$, we see that $\rho (t,b^+(t))\leqq \rho _0(b)\leqq \rho _{*}$ for all $t\geqq 0$. From (1.9)$_{1}$ and (5.2), there exists a positive constant $\tilde{C}$ depending only on $(\gamma _1, \kappa _1)$ such that

$$\begin{aligned} \rho _{\tau }(\tau ,M)=-\frac{1}{\varepsilon }\, \big (\frac{\rho P}{\mu (\rho )}\big )(\tau ,M) \geqq -\frac{\tilde{C}}{\varepsilon }\big (\rho (\tau ,M)\big )^{\gamma _1+1- \alpha }, \end{aligned}$$

which implies

$$\begin{aligned} \rho (\tau ,M)\geqq \rho _{0}(M) \Big (1+\frac{\tilde{C}(\gamma _1-\alpha )}{\varepsilon }\big (\rho _{0}(M)\big )^{\gamma _1-\alpha }\tau \Big )^{-\frac{1}{\gamma _1-\alpha }}. \end{aligned}$$

Therefore, in the Eulerian coordinates,

5.7 $$\begin{aligned} \rho (t,b^+(t))\geqq \rho _{0}(b)\Big (1+\frac{\tilde{C}(\gamma _1-\alpha )}{\varepsilon } (\rho _{0}(b))^{\gamma _1-\alpha }t\Big )^{-\frac{1}{\gamma _1-\alpha }} \qquad \text{ for } t\geqq 0. \end{aligned}$$

Notice that, in the Lagrangian coordinates, $\rho _0(M)=\rho _0(0)$ since $\rho _0(b)=\rho _0(-b)$ in the Eulerian coordinates. Therefore, by the uniqueness of solutions of the ordinary differential equation: $\rho _{\tau }(\tau ,\cdot )=-\frac{1}{\varepsilon }\big (\frac{\rho P}{\mu (\rho )}\big )(\tau ,\cdot )$ with the same initial data, we conclude that

5.8 $$\begin{aligned} \rho (t,b^+(t))=\rho (t,b^-(t)). \end{aligned}$$

This implies that $P(\rho (t,b^+(t)))=P(\rho (t,b^-(t)))$. This indicates, in particular, that the boundary term in (5.9) below is nonnegative.

#### Lemma 5.5

Let $\rho ^{\gamma _1}_0(\pm b)b\leqq C_0$. Then, for any given $T>0$ and for any $t\in [0,T]$,

5.9 $$\begin{aligned}&\varepsilon ^2\int ^{b^+(t)}_{b^-(t)}\big (\rho ^{2\alpha -3}\rho ^2_x\big )(t,x)\,\textrm{d}x +\varepsilon \int ^t_0\int ^{b^+(t)}_{b^-(t)}\big (P'(\rho )\rho ^{\alpha -2}\rho ^2_x\big )(s,x)\,\textrm{d}x\textrm{d}s\nonumber \\&+\frac{1}{\varepsilon }\int ^{t}_0\Big (\big (\frac{P'(\rho )P(\rho )\rho }{\mu (\rho )}\big )(s,b^+(s))b^+(s) -\big (\frac{P'(\rho )P(\rho )\rho }{\mu (\rho )}\big )(s,b^-(s))b^-(s)\Big )\,\textrm{d}s\nonumber \\&+\big (P(\rho (t,b^+(t)))b^+(t)-P(\rho (t,b^-(t)))b^-(t)\big )\leqq C. \end{aligned}$$

#### Proof

Similarly to (3.16), we obtain

5.10 $$\begin{aligned}&\Big (\frac{(u+\frac{\varepsilon }{\alpha }(\rho ^{\alpha })_{\xi })^2}{2}+e(\rho )+\frac{1}{2}\rho W*\rho \Big )_{\tau } +\big (P(\rho )u\big )_{\xi }+\varepsilon P'(\rho )\rho ^{\alpha -1}(\rho _{\xi })^2\nonumber \\&+\big (u+\frac{\varepsilon }{\alpha }(\rho ^{\alpha })_{\xi }\big )\big (-\lambda u-V+\rho (W*\rho )_{\xi }\big )=0. \end{aligned}$$

Using (3.3)$_{1}$ and (3.4), we have

$$\begin{aligned} \rho _{\tau }(\tau ,M)=-\frac{1}{\varepsilon }\Big (\frac{\rho P}{\mu (\rho )}\Big )(\tau ,M), \qquad \rho _{\tau }(\tau ,0)=-\frac{1}{\varepsilon }\Big (\frac{\rho P}{\mu (\rho )}\Big )(\tau ,0), \end{aligned}$$

so that

$$\begin{aligned}&\big (P(\rho )u\big )(\tau ,M)-\big (P(\rho )u\big )(\tau ,0)\nonumber \\  &=P(\rho )(\tau ,M)\frac{\text {d}}{\text {d}\tau }b^+(\tau )-P(\rho )(\tau ,0)\frac{\text {d}}{\text {d}\tau }b^-(\tau )\nonumber \\  &=\big (P(\rho )(\tau ,M)b^+(\tau )\big )_{\tau } -\big (P(\rho )(\tau ,0)b^-(\tau )\big )_{\tau }\nonumber \\  &\quad \, +\Big (P'(\rho )(\tau ,0)\rho _{\tau }(\tau ,0)b^{-}(\tau )-P'(\rho )(\tau ,M)\rho _{\tau }(\tau ,M)b^{+}(\tau )\Big )\nonumber \\  &=\big (P(\rho )(\tau ,M)b^+(\tau )\big )_{\tau }-\big (P(\rho )(\tau ,0)b^-(\tau )\big )_{\tau }\nonumber \\  &\quad \, +\frac{1}{\varepsilon }\Big (\frac{P'(\rho )P(\rho )\rho }{\mu (\rho )}\Big )(\tau ,M)b^+(\tau ) -\frac{1}{\varepsilon }\Big (\frac{P'(\rho )P(\rho )\rho }{\mu (\rho )}\Big )(\tau ,0)b^-(\tau ).\nonumber \end{aligned}$$

Integrating (5.10) over [0, *M*], we arrive at

5.11 $$\begin{aligned}&\frac{\textrm{d}}{\textrm{d}\tau }\int ^{M}_0\Big (\frac{1}{2}\big (u+\frac{\varepsilon }{\alpha }(\rho ^{\alpha })_{\xi }\big )^2+e(\rho )\Big )\,\textrm{d}\xi +\varepsilon \int ^M_0P'(\rho )\rho ^{\alpha -1}(\rho _{\xi })^2\,\textrm{d}\xi \nonumber \\&\,\,\,+\big (P(\rho )(\tau ,M)b^+(\tau )-P(\rho )(\tau ,0)b^-(\tau )\big )_{\tau }\nonumber \\&\,\,\,+\frac{1}{\varepsilon }\Big (\frac{P'(\rho )P(\rho )\rho }{\mu (\rho )}\Big )(\tau ,M)b^+(\tau ) -\frac{1}{\varepsilon }\Big (\frac{P'(\rho )P(\rho )\rho }{\mu (\rho )}\Big )(\tau ,0)b^-(\tau )\nonumber \\&=\int ^M_0\big (u+\frac{\varepsilon }{\alpha }(\rho ^{\alpha })_{\xi }\big )\big (-\lambda u-V+\rho (W*\rho )_{\xi }\big )\,\textrm{d}\xi . \end{aligned}$$

Integrating (5.11) over $[0,\tau ],$ we have

5.12 $$\begin{aligned}&\int ^M_0\Big (\frac{1}{2}\big (u+\frac{\varepsilon }{\alpha }(\rho ^{\alpha })_{\xi }\big )^2+e(\rho )\Big )\,\textrm{d}\xi +\varepsilon \int ^{\tau }_0\int ^M_0P'(\rho )\rho ^{\alpha -1}(\rho _{\xi })^2\,\textrm{d}\xi \textrm{d}s\nonumber \\&\quad +\Big (P(\rho )(\tau ,M)b^+(\tau )-P(\rho )(\tau ,0)b^-(\tau )\Big )\nonumber \\&\quad +\frac{1}{\varepsilon }\int ^{\tau }_0\Big (\Big (\frac{P'(\rho )P(\rho )\rho }{\mu (\rho )}\Big )(\tau ,M)b^+(\tau ) -\Big (\frac{P'(\rho )P(\rho )\rho }{\mu (\rho )}\Big )(\tau ,0)b^-(\tau )\Big )\,\textrm{d}s\nonumber \\&\leqq C\int ^M_0\Big (\frac{1}{2}\big (u_0+\frac{\varepsilon }{\alpha }(\rho ^{\alpha }_0)_{\xi }\big )^2 +e(\rho _0)\Big )\,\textrm{d}\xi +C\big (P(\rho _0(M))+P(\rho _0(0))\big )\, b+C. \end{aligned}$$

Using $b\geqq \rho ^{-\gamma _1}_{*},$ we see that $P(\rho _0(\pm b))\, b\leqq C_0$ so that the second term on the RHS of (5.12) is uniformly bounded. This completes the proof. $\square$

Motivated by [13], to take the limit: $b\rightarrow \infty$, we need to make sure that domain $\Omega _T=[b^-(t),b^+(t)]$ can expand to the whole physical space $\mathbb {R}$.

#### Lemma 5.6

Let $0\leqq \alpha <\gamma _1,$ $p>\frac{\gamma _1}{\gamma _1-\alpha },$ and $b:=\varepsilon ^{-p}$. Then, given any $T>0$, there exists $\varepsilon _0=\varepsilon _0(\alpha ,\gamma _1,\gamma _2,T)>0$ such that, for any $\varepsilon \in (0,\varepsilon _0]$,

$$ \pm b^\pm (t)\geqq \frac{1}{2} b \qquad \,\, \text{ for } t\in [0,T]. $$

#### Proof

We divide the proof into three steps:

*Step 1.* Using (5.1) and (5.9), we have

5.13 $$\begin{aligned} \varepsilon \rho ^{\beta }(t,x)&=\varepsilon \big (\rho ^{\beta }(t,x)-\rho ^{\beta }(t,b^-(t))+\rho ^{\beta }(t,b^-(t))\big )\nonumber \\&\leqq \varepsilon \beta \int ^{b^+(t)}_{b^-(t)}\rho ^{\beta -1}|\rho _x|\,\textrm{d}x+\varepsilon \rho ^{\beta }_0(b)\nonumber \\&\leqq \beta \Big (\varepsilon ^2\int ^{b^+(t)}_{b^-(t)}\rho ^{2\alpha -3}\rho ^2_x\,\textrm{d}x\Big )^{\frac{1}{2}}\Big (\int ^{b^+(t)}_{b^-(t)}\rho ^{2(\beta - \alpha )+1}\,\textrm{d}x\Big )^{\frac{1}{2}}+\varepsilon \rho ^{\beta }_0(b)\nonumber \\&\leqq C(\mathcal {E}^{\varepsilon }_1)\Big (\int ^{b^+(t)}_{b^-(t)}\rho ^{2(\beta - \alpha )+1}\,\textrm{d}x\Big )^{\frac{1}{2}}+C\le C, \end{aligned}$$

where we have used (5.6) and $\beta =\alpha +\frac{\gamma _2-1}{2}.$

*Step 2.* It follows from the boundary condition (2.11) that

5.14 $$\begin{aligned} b^+(t)-b^-(t)=2b+\int ^{t}_0\big (u(s,b^+(s))-u^-(s,b^-(s))\big )\,\textrm{d}s. \end{aligned}$$

Using (5.8), we obtain

5.15 $$\begin{aligned}&\big |u(t,b^+(t))-u(t,b^-(t))\big |\nonumber \\&=\frac{1}{\rho ^l(t,b^+(t))}\big |(\rho ^lu)(t,b^+(t))-(\rho ^lu)(t,b^-(t))\big |\nonumber \\&=\frac{1}{\rho ^l(t,b^+(t))} \bigg |\int ^{b^+(t)}_{b^-(t)}(\rho ^l u)_x\,\textrm{d}x\bigg |\nonumber \\&=\frac{1}{\rho ^l(t,b^+(t))}\bigg |\int ^{b^+(t)}_{b^-(t)}\big (l \rho ^{l-1}\rho _xu+\rho ^l u_x\big )\,\textrm{d}x\bigg |\nonumber \\&\leqq \frac{1}{\rho ^l(t,b^+(t))}\bigg \{l\Big (\int ^{b^+(t)}_{b^-(t)}\varepsilon P'(\rho )\rho ^{\alpha -2}\rho ^2_x\,\textrm{d}x\Big )^{\frac{1}{2}}\Big (\int ^{b^+(t)}_{b^-(t)}\varepsilon ^{-1}\frac{\rho ^{2l-\alpha }}{P'(\rho )}u^2\,\textrm{d}x\Big )^{\frac{1}{2}}\nonumber \\&\qquad \qquad \qquad \qquad \,\,\,\, +\Big (\int ^{b^+(t)}_{b^-(t)}\varepsilon \rho ^{\alpha }u^2_x\,\textrm{d}x\Big )^{\frac{1}{2}} \Big (\int ^{b^+(t)}_{b^-(t)}\varepsilon ^{-1}\rho ^{2l-\alpha }\,\textrm{d}x\Big )^{\frac{1}{2}}\bigg \}. \end{aligned}$$

Taking $$2l-(\alpha +\gamma _2)+1=1,$$ $$ {i.e.,}$$ $$l=\frac{\alpha +\gamma _2}{2},$$ so that $$2l-\alpha =\gamma _2.$$ We also have

5.16 $$\begin{aligned} \bigg |\int ^{b^+(t)}_{b^-(t)}\varepsilon ^{-1}\frac{\rho ^{2l-\alpha }}{P'(\rho )}u^2\,\textrm{d}x\bigg |&\leqq \bigg |\int ^{b^+(t)}_{b^-(t)}\varepsilon ^{-1}\frac{\rho ^{2l-\alpha }}{P'(\rho )}u^2\textbf{I}_{\{\rho \leqq \rho _{*}\}}\,\textrm{d}x\bigg |\nonumber \\&\quad +\bigg |\int ^{b^+(t)}_{b^-(t)}\varepsilon ^{-1}\frac{\rho ^{2l-\alpha }}{P'(\rho )}u^2\textbf{I}_{\{\rho _{*}\leqq \rho \leqq \rho ^{*}\}}\,\textrm{d}x\bigg |\nonumber \\&\quad \,\,+\bigg |\int ^{b^+(t)}_{b^-(t)}\varepsilon ^{-1}\frac{\rho ^{2l-\alpha }}{P'(\rho )}u^2\textbf{I}_{\{\rho \geqq \rho ^{*}\}}\,\textrm{d}x\bigg |\nonumber \\&\leqq C(\rho ^{*},\rho _{*}). \end{aligned}$$

It follows from (5.15)–(5.16), (3.2), and (5.5) that

$$\begin{aligned}&\int ^t_0\big |u(s,b^+(s))-u(s,b^-(s))\big |\,\text {d}s\nonumber \\  &\leqq C\varepsilon ^{-\frac{1}{2}}\Big (\int ^t_0(\rho (s,b^+(s)))^{-\alpha -\gamma _2}\,\text {d}s\Big )^{\frac{1}{2}}\nonumber \\  &\quad \,\times \bigg \{\Big (\int ^t_0\int ^{b^+(s)}_{b^-(s)}\varepsilon P'(\rho )\rho ^{\alpha -2}\rho ^2_x\,\text {d}x\text {d}s\Big )^{\frac{1}{2}} +\Big (\int ^t_0\int ^{b^+(s)}_{b^-(s)}\varepsilon \rho ^{\alpha }u^2_x\,\text {d}x\text {d}s\Big )^{\frac{1}{2}}\bigg \}\nonumber \\  &\leqq C\varepsilon ^{-\frac{1}{2}}\Big (\int ^t_0(\rho (s,b^+(s)))^{-\alpha -\gamma _2}\,\text {d}s\Big )^{\frac{1}{2}}. \end{aligned}$$

Using (5.1) and (5.9), we obtain

5.17 $$\begin{aligned}&\int ^t_0\big |u(s,b^+(s)-u(s,b^-(s))\big |\,\textrm{d}s\nonumber \\&\leqq C\varepsilon ^{-\frac{1}{2}}\Big (\int ^t_0(\rho (s,b^+(s)))^{-\alpha -\gamma _2}\,\textrm{d}s\Big )^{\frac{1}{2}}\nonumber \\&\quad \,\times \bigg \{\Big (\int ^t_0\int ^{b^+(s)}_{b^-(s)}\varepsilon P'(\rho )\rho ^{\alpha -2}\rho ^2_x\,\textrm{d}x\textrm{d}s\Big )^{\frac{1}{2}} +\Big (\int ^t_0\int ^{b^+(s)}_{b^-(s)}\varepsilon \rho ^{\alpha }u^2_x\,\textrm{d}x\textrm{d}s\Big )^{\frac{1}{2}}\bigg \}\nonumber \\&\leqq C\varepsilon ^{-\frac{1}{2}}\Big (\int ^t_0(\rho (s,b^+(s)))^{-\alpha -\gamma _2}\,\textrm{d}s\Big )^{\frac{1}{2}}. \end{aligned}$$

Since $\rho _0(b)\leqq Cb^{-\frac{1}{\gamma _1}},$ we take $b\gtrsim (\frac{1}{\varepsilon })^{\frac{\gamma _1}{\gamma _1-\alpha }}$ to obtain

5.18 $$\begin{aligned} \frac{\tilde{C}(\gamma _1-\alpha )}{\varepsilon }(\rho _0(b))^{\gamma _1-\alpha }\leqq \frac{C\tilde{C}(\gamma _1-\alpha )}{\varepsilon }b^{-\frac{\gamma _1-\alpha }{\gamma _1}}\leqq C. \end{aligned}$$

It follows from (5.7) and (5.18) that, for $0\leqq s\leqq T,$

$$\begin{aligned} \rho (s,b^+(s))\geqq \rho _{0}(b)\Big (1+\frac{\tilde{C}(\gamma _1-\alpha )}{\varepsilon } (\rho _{0}(b))^{\gamma _1-\alpha }s\Big )^{-\frac{1}{\gamma _1-\alpha }}\geqq \frac{1}{C}(1+T)^{-\frac{1}{\gamma _1-\alpha }}\rho _0(b). \end{aligned}$$

Then we have

5.19 $$\begin{aligned} \int ^t_0(\rho (s,b^+(s)))^{-\alpha -\gamma _2}\,\textrm{d}s\leqq C(1+T)^{\frac{\alpha +\gamma _2}{\gamma _1-\alpha }}(\rho _0(b))^{-\alpha -\gamma _2}. \end{aligned}$$

Take $b=\varepsilon ^{-p}$ with $p>\frac{\gamma _1}{\gamma _1-\alpha }$. For any given $T\geqq 1$, choose

$$\begin{aligned} \varepsilon _1:=\big (C_1(1+T)^{\frac{\alpha +\gamma _2}{2(\gamma _1-\alpha )}}\big )^{-\frac{2\gamma _1}{p(\gamma _1-\alpha )-\gamma _1}}, \end{aligned}$$

where $C_1\geqq 1$ is a large constant depending only on $C_0$. Then, for any $\varepsilon \in (0,\varepsilon _1],$ it follows from (5.17) and (5.19) that

$$\begin{aligned} \int ^t_0\big |u(s,b^+(s))-u(s,b^-(s))\big |\,\textrm{d}s&\leqq C_1\varepsilon ^{-\frac{1}{2}}(1+T)^{\frac{\alpha +\gamma _2}{2(\gamma _1-\alpha )}}(\rho _0(b))^{-\frac{\alpha +\gamma _2}{2}}\\&=C_1(1+T)^{\frac{\alpha +\gamma _2}{2(\gamma _1-\alpha )}}\varepsilon ^{-\frac{1}{2}-\frac{p(\alpha +\gamma _2)}{2\gamma _1}}\\&\leqq b\, C_1(1+T)^{\frac{\alpha +\gamma _2}{2(\gamma _1-\alpha )}}\varepsilon ^{p-\frac{1}{2}-\frac{p(\alpha +\gamma _2)}{2\gamma _1}}\\&\leqq b\,C_1(1+T)^{\frac{\alpha +\gamma _2}{2(\gamma _1-\alpha )}}\varepsilon ^{\frac{p(\gamma _1-\alpha )-\gamma _1}{2\gamma _1}}\leqq b. \end{aligned}$$

Using (5.14), we conclude that

5.20 $$\begin{aligned} b\leqq b^+(t)-b^-(t)\leqq 3b. \end{aligned}$$

*Step 3.* There exists $x_0(t)\in (b^-(t),b^+(t))$ such that

$$ (\rho u^2)(t,x_0(t))\,\big (b^+(t)-b^-(t)\big ) =\int ^{b^+(t)}_{b^-(t)}(\rho u^2)(t,x)\,\textrm{d}x\leqq C. $$

Using (5.20), we have

5.21 $$\begin{aligned} (\rho u^2)(t,x_0(t))\leqq C(\mathcal {E}^{\varepsilon }_0)\,\big (b^+(t)-b^-(t)\big )^{-1}\leqq C\,b^{-1}. \end{aligned}$$

It follows from (2.11) that

5.22 $$\begin{aligned} b^+(t)=b+\int ^t_0u(s,b^+(s))\,\textrm{d}s=b+\int ^t_0\frac{1}{\rho ^l(s,b^+(s))}(\rho ^lu)(s,b^+(s))\,\textrm{d}s, \end{aligned}$$

where $l=\frac{\alpha +\gamma _2}{2}.$ Similar to those as in (5.15), we use (5.13) and (5.21) to obtain

5.23 $$\begin{aligned} (\rho ^lu)(s,b^+(s))&=(\rho ^lu)(s,b^+(s))-(\rho ^lu)(s,x_0(s))+(\rho ^l u)(s,x_0(s))\nonumber \\&=\int ^{b^+(s)}_{b^-(s)}\big (l\rho ^{l-1}\rho _xu+\rho ^lu_x\big )\,\textrm{d}x+(\rho ^lu)(s,x_0(s))\nonumber \\&\leqq \Big (\int ^{b^+(s)}_{b^-(s)}P'(\rho )\rho ^{\alpha -2}\rho ^2_x\,\textrm{d}x\Big )^{\frac{1}{2}}\Big (\int ^{b^+(s)}_{b^-(s)}\frac{\rho ^{2l-\alpha }}{P'(\rho )}u^2\,\textrm{d}x\Big )^{\frac{1}{2}}\nonumber \\&\quad \,+\Big (\int ^{b^+(s)}_{b^-(s)}\rho ^{\alpha }u^2_x\,\textrm{d}x\Big )^{\frac{1}{2}}\Big (\int ^{b^+(s)}_{b^-(s)}\rho ^{2l-\alpha }\,\textrm{d}x\Big )^{\frac{1}{2}}\nonumber \\&\quad +C b^{-\frac{1}{2}}\,(\rho (s,x_0(s)))^{\frac{\alpha +\gamma _2-1}{2}}\nonumber \\&\leqq C \varepsilon ^{-\frac{1}{2}}\bigg \{\Big (\varepsilon \int ^{b^+(s)}_{b^-(s)}P'(\rho )\rho ^{\alpha -2}\rho ^2_x\,\textrm{d}x\Big )^{\frac{1}{2}}+\Big (\varepsilon \int ^{b^+(s)}_{b^-(s)}\rho ^{\alpha }u^2_x\,\textrm{d}x\Big )^{\frac{1}{2}}\bigg \}\nonumber \\&\quad +C \varepsilon ^{-1}b^{-\frac{1}{2}}. \end{aligned}$$

Using (5.5), (5.9), (5.19), and (5.23), we have

5.24 $$\begin{aligned}&\bigg |\int ^t_0\frac{1}{\rho ^l(s,b^+(s))}(\rho ^lu)(s,b^+(s))\,\textrm{d}s\bigg |\nonumber \\&\leqq C\varepsilon ^{-\frac{1}{2}}\Big (\int ^t_0(\rho (s,b^+(s)))^{-(\alpha +\gamma _2)}\,\textrm{d}s\Big )^{\frac{1}{2}}\nonumber \\&\quad \,\,\,\times \bigg \{\Big (\int ^t_0\int ^{b^+(s)}_{b^-(s)}P'(\rho )\rho ^{\alpha -2}\rho ^2_x\,\textrm{d}x \textrm{d}s\Big )^{\frac{1}{2}} +\Big (\int ^t_0\int ^{b^+(s)}_{b^-(s)}\varepsilon \rho ^{\alpha }u^2_x\,\textrm{d}x\textrm{d}s\Big )^{\frac{1}{2}}\bigg \}\nonumber \\&\quad +C\varepsilon ^{-1}b^{-\frac{1}{2}}\int ^t_0(\rho (s,b^+(s)))^{-\frac{\alpha +\gamma _2}{2}}\,\textrm{d}s\nonumber \\&\leqq C \bigg \{\varepsilon ^{-\frac{1}{2}}\Big (\int ^t_0(\rho (s,b^+(s)))^{-(\alpha +\gamma _2)}\,\textrm{d}s\Big )^{\frac{1}{2}} +\varepsilon ^{-1}b^{-\frac{1}{2}}\int ^t_0(\rho (s,b^+(s)))^{-\frac{\gamma _2+\alpha }{2}}\,\textrm{d}s\bigg \}\nonumber \\&\leqq b\,C \Big (\varepsilon ^{-\frac{1}{2}}b^{-1}(1+T)^{\frac{\alpha +\gamma _2}{2(\gamma _1-\alpha )}} +\varepsilon ^{-1}b^{-\frac{3}{2}}(1+T)^{\frac{3\gamma _2-\alpha }{2(\gamma _1-\alpha )}}\Big )\, b^{\frac{\alpha +\gamma _2}{2\gamma _1}}\nonumber \\&\leqq b\, C_2(1+T)^{\frac{3\gamma _2-\alpha }{2(\gamma _1-\alpha )}}\varepsilon ^{\frac{3}{2}p-1-p\frac{\alpha +\gamma _2}{2\gamma _1}}\nonumber \\&\leqq b\, C_2(1+T)^{\frac{3\gamma _2-\alpha }{2(\gamma _1-\alpha )}}\varepsilon ^{\frac{p(\gamma _1-\alpha )-\gamma _1}{2\gamma _1}} \leqq \frac{1}{2}b \end{aligned}$$

for any $\varepsilon \in (0,\varepsilon _2]$ with $\varepsilon _2:=\big (2C_2(1+T)^{\frac{3\gamma _2-\alpha }{2(\gamma _1-\alpha )}}\big )^{-\frac{2\gamma _1}{p(\gamma _1-\alpha )-\gamma _1}}.$

Take $\tilde{C_0}:=2\max \{C_1,C_2\}$ and $\varepsilon _0:=\big (\tilde{C}_0(1+T)^{\frac{3\gamma _2-\alpha }{2(\gamma _1-\alpha )}}\big )^{-\frac{2\gamma _1}{p(\gamma _1-\alpha )-\gamma _1}}.$ Then, for any $\varepsilon \in (0,\varepsilon _0]$, we obtain from (5.22) and (5.24) that $b^+(t)\geqq \frac{1}{2}b.$

Similarly, it can be proved that $b^-(t)\leqq -\frac{1}{2}b.$ This completes the proof. $\square$

#### Lemma 5.7

(Higher integrability of the density) Let $(\rho ,u)$ be the smooth solution of (1.9) and (2.10)–(2.13). Then, under the assumption of Lemma 5.6,

5.25 $$\begin{aligned} \int _0^T\int _K (\rho P(\rho ))(t,x)\,\textrm{d}x \textrm{d}t\leqq C(K) \qquad \text{ for } \text{ any } K\Subset [b^-(t),b^+(t)]\,\, \text{ for } \text{ any } t \in [0,T]. \end{aligned}$$

#### Proof

We divide the proof into two steps.

*Step 1.* For given $K\Subset [b^-(t),b^+(t)]$ for any $t\in [0,T]$, there exist $r_1$ and $r_2$ such that $K\Subset (r_1,r_2)\Subset [b^-(t),b^+(t)]$. Let *w*(*x*) be a smooth, compactly supported function with $\text {supp}\,w\subseteq (r_1,r_2)$ and $w(x)=1$ for $x\in K$. Multiplying (1.9)$_{2}$ by *w*(*x*), we have

5.26 $$\begin{aligned}&(\rho u w)_t+\big ((\rho u^2+P(\rho ))w\big )_x\nonumber \\&=(\rho u^2+P(\rho ))w_x+\varepsilon (\rho ^{\alpha }w u_x)_x-\varepsilon \rho ^{\alpha }u_xw_x+\lambda \rho uw+\rho Vw-\rho w\,\partial _xW*\rho . \end{aligned}$$

Integrating (5.26) over $$[r_1,x)$$ to obtain

5.27 $$\begin{aligned} (\rho u^2+P(\rho ))w&=\varepsilon \rho ^{\alpha }wu_x+\int ^{x}_{r_1}\Big ((\rho u^2+P(\rho ))w_y-\varepsilon \rho ^{\alpha }u_yw_y\Big )\,\textrm{d}y-\frac{\textrm{d}}{\textrm{d}t}\int ^x_{r_1}\rho uw\,\textrm{d}y\nonumber \\&\quad +\int ^x_{r_1}\lambda \rho uw\,\textrm{d}y-\int ^x_{r_1}\rho w\,\partial _x W*\rho \,\textrm{d}y+\int ^x_{r_1}\rho Vw\,\textrm{d}y . \end{aligned}$$

Multiplying (5.27) by $\rho w$ and performing a direct calculation, we have

5.28 $$\begin{aligned} \rho P(\rho ) w^2&=\varepsilon \rho ^{\alpha +1}w^2u_x-\Big (\rho w\int ^x_{r_1}\rho uw\,\textrm{d}y\Big )_t-\Big (\rho uw\int ^x_{r_1}\rho uw\,\textrm{d}y\Big )_x\nonumber \\&\quad +\rho uw_x\int ^x_{r_1}\rho uw\,\textrm{d}y+\rho w\int ^x_{r_1}\Big ((\rho u^2+P(\rho ))w_y-\varepsilon \rho ^{\alpha }u_yw_y\Big )\,\textrm{d}y\nonumber \\&\quad +\lambda \rho w\int ^x_{r_1}\rho u w\,\textrm{d}y-\rho w\int ^x_{r_1}\rho w\,\partial _xW*\rho \,\textrm{d}y+\rho w\int ^x_{r_1}\rho Vw\,\textrm{d}y\nonumber \\&=\sum ^{8}_{i=1}K_{i}. \end{aligned}$$

*Step 2.* To estimate $K_i, i=1,\ldots , 8$, in (5.28), we first notice that

5.29 $$\begin{aligned} \int _{b^{-}(t)}^{b^+(t)}\rho |u|\, \textrm{d}x\leqq \int _{b^-(t)}^{b^+(t)} (\rho +\rho u^2)\,\textrm{d}x\leqq C. \end{aligned}$$

Then it follows from (5.29) that

5.30 $$\begin{aligned} \bigg | \int _0^T\int _{r_1}^{r_2} K_2\, \textrm{d}x\textrm{d}t \bigg |&=\bigg | \int _0^T\int _{r_1}^{r_2} \Big (\rho w\int ^x_{r_1}\rho uw\,\textrm{d}y\Big )_t\, \textrm{d}x\textrm{d}t \bigg |\nonumber \\&\leqq \bigg |\int ^{r_2}_{r_1}\Big (\rho w\int ^x_{r_1}\rho u w\textrm{d}y\Big )(T,x)\,\textrm{d}x\bigg |\nonumber \\&\quad +\bigg |\int ^{r_2}_{r_1}\Big (\rho w\int ^x_{r_1}\rho u w\textrm{d}y\Big )(0,x)\,\textrm{d}x\bigg |\nonumber \\&\leqq C. \end{aligned}$$

For $K_1$, we have

$$\begin{aligned} \bigg |\int _0^T\int _{r_1}^{r_2} K_1\,\textrm{d}x\textrm{d}t \bigg |&=\bigg |\int ^T_0\int ^{r_2}_{r_1}\rho ^{\alpha +1}\omega ^2u_x\,\textrm{d}x\textrm{d}t\bigg |\nonumber \\&\leqq \varepsilon \int ^T_0\int ^{r_2}_{r_1}\rho ^{\alpha }|u_x|^2\omega ^2\,\textrm{d}x\textrm{d}t+\varepsilon \int ^T_0\int ^{r_2}_{r_1}\rho ^{\alpha +2}w^2\,\textrm{d}x\textrm{d}t\nonumber \\&\leqq C +\varepsilon \int ^T_0\int ^{r_2}_{r_1}\rho ^{\alpha +2}w^2\,\textrm{d}x\textrm{d}t. \end{aligned}$$

We now estimate $\int _{0}^{T}\int _{r_1}^{r_2}\varepsilon \rho ^{\alpha +2}\omega ^2\,\textrm{d}x\textrm{d}t$. For any fixed $t\in [0,T]$, denoting

$$ A(t):=\big \{x\in [r_1,r_2]\,:\, \rho (t,x)\geqq \rho ^{*}\big \}, $$

then it follows from (3.2) that $|A(t)|\leqq C(r_1,r_2,\rho ^{*})M$. For any $x\in A(t)$, let $x_{0}$ be the closest point to *r* so that $\rho (t,x_{0})=\rho ^{*}$ with $|x-x_{0}|\leqq |A(t)|\leqq C(r_1,r_2,\rho ^{*})M$. Then, for any smooth function $f(\rho )$,

$$\begin{aligned} \sup _{x\in A(t)}f(\rho (t,x))\omega ^{2}(x)&\leqq f(\rho (t,x_0))\omega ^2(x_0) +\bigg |\int _{x_0}^{x}\partial _y\big (f(\rho (t,y))\omega ^{2}(y)\big )\,\textrm{d}y\bigg |\nonumber \\&\leqq C(\Vert \omega \Vert _{C^1})|f(\rho ^{*})| +\int _{A(t)}\big |\partial _y\big (f(\rho (t,y))\omega ^{2}(y)\big )\big |\,\textrm{d}y.\nonumber \end{aligned}$$

Recalling (5.3) and (5.4), we notice that $P(\rho )\cong \rho ^{\gamma _2}$ and $e(\rho )\cong \rho ^{\gamma _2-1}$ for any $x\in A(t)$. Then

$$\begin{aligned}&\varepsilon \int _{0}^{T}\int _{r_1}^{r_2}\rho ^{\alpha +2}\omega ^2\,\textrm{d}x\textrm{d}t\nonumber \\&= \varepsilon \int _{0}^{T}\int _{r_1}^{r_2}\rho ^{\alpha +2}  \textbf{I}_{\{\rho \leqq \rho ^{*}\}}\omega ^2\,\textrm{d}x\textrm{d}t + \varepsilon \int _{0}^{T}\int _{r_1}^{r_2}\rho ^{\alpha +2} \textbf{I}_{\{\rho \geqq \rho ^{*}\}}\omega ^2\,\textrm{d}x\textrm{d}t\nonumber \\&\leqq C(\rho ^{*}) + C \, \varepsilon \int _{0}^{T}\Big (\int _{r_1}^{r_2}\rho e(\rho )\textrm{d}x\Big ) \sup _{x\in A(t)} \Big (\frac{\rho ^{\alpha +1}}{e(\rho )}\omega ^2\Big )\textrm{d}t\nonumber \\&\leqq C(\rho ^{*})+ C\, \varepsilon \int _{0}^{T}\int _{A(t)}\Big \vert \Big (\frac{\rho ^{\alpha +1}}{e(\rho )}\omega ^2\Big )_{x}\Big \vert \,\textrm{d}x\textrm{d}t\nonumber \\&\leqq C\,\varepsilon \int _{0}^{T}\int _{A(t)}\Big (\big (\frac{(\alpha +1)\rho ^{\alpha }}{e(\rho )}-\frac{\rho ^{\alpha -1}P(\rho )}{e(\rho )^2}\big ) |\rho _{x}|\omega ^2+\frac{\rho ^{\alpha +1}}{e(\rho )}\omega |\omega _{x}|\Big )\,\textrm{d}x\textrm{d}t +C(\rho ^{*}). \end{aligned}$$

A direct calculation shows that

$$\begin{aligned}&\int _{0}^{T}\int _{A(t)}\varepsilon \Big (\frac{(\alpha +1)\rho ^{\alpha }}{e(\rho )}-\frac{\rho ^{\alpha -1}P(\rho )}{e(\rho )^2}\Big ) |\rho _{x}|\,\omega ^2\,\textrm{d}x\textrm{d}t\nonumber \\&\leqq \int _{0}^{T}\int _{A(t)}\varepsilon \frac{P'(\rho )}{\rho ^{2-\alpha }}|\rho _{x}|^2\omega ^2\,\textrm{d}x\textrm{d}t +\int _{0}^{T}\int _{A(t)}\varepsilon \Big (\frac{(\alpha +1)\rho ^{\alpha }}{e(\rho )}-\frac{\rho ^{\alpha -1}P(\rho )}{e(\rho )^2}\Big )^2\frac{\rho ^{2-\alpha }}{P'(\rho )}\omega ^2\,\textrm{d}x\textrm{d}t \nonumber \\&\leqq C+\int _{0}^{T}\int _{A(t)}\varepsilon \rho ^{5+\alpha -3\gamma _2}\omega ^2\,\textrm{d}x\textrm{d}t\nonumber \\&\leqq {\left\{ \begin{array}{ll} C+\varepsilon \int ^T_0\int ^{r_2}_{r_1}(\rho ^{\beta }+1)\,\textrm{d}x\textrm{d}t \quad & \text {if } 5+\alpha -3\gamma _2\leqq \beta ,\\ C +\frac{\varepsilon }{2}\int ^T_0\int ^{r_2}_{r_1}\rho ^{\alpha +2}\omega ^2\,\textrm{d}x\textrm{d}t\,\,\,& \text {if }5+\alpha -3\gamma _2\geqq \beta . \end{array}\right. } \end{aligned}$$

We also have

$$\begin{aligned}&\int ^T_0\int _{A(t)}\varepsilon \frac{\rho ^{\alpha +1}}{e(\rho )}\omega |\omega _x|\,\textrm{d}x\textrm{d}t\nonumber \\&\leqq \int ^T_0\int ^{r_2}_{r_1}\varepsilon \rho ^{\alpha -\gamma _1+2}\omega \,\textrm{d}x\textrm{d}t\nonumber \\&\leqq \frac{\varepsilon }{2}\int ^T_0\int ^{r_2}_{r_1}\rho ^{\alpha +2}\omega ^2\,\textrm{d}x\textrm{d}t+C(r_1,r_2,\rho ^{*}). \end{aligned}$$

Notice that

$$\begin{aligned}&\bigg | \int _0^T\int _{r_1}^{r_2}K_3\, \textrm{d}x\textrm{d}t \bigg | =\bigg | \int _0^T\int _{r_1}^{r_2} \Big (\rho uw\int ^x_{r_1}\rho uw\textrm{d}y\Big )_x\, \textrm{d}x\textrm{d}t \bigg |=0,\\&\bigg | \int _0^T\int _{r_1}^{r_2} K_4\, \textrm{d}x\textrm{d}t \bigg | =\bigg | \int _0^T\int _{r_1}^{r_2} \rho uw_x\Big (\int ^x_{r_1}\rho uw\textrm{d}y\Big )\, \textrm{d}x\textrm{d}t \bigg |\leqq C. \end{aligned}$$

Using the fact that $\alpha \leqq \gamma _2,$ we have

$$\begin{aligned} \bigg | \int _0^T\int _{r_1}^{r_2} K_5\, \textrm{d}x\textrm{d}t \bigg |&=\bigg |\int _0^T\int _{r_1}^{r_2} \Big (\rho uw\int ^x_{r_1}\big ((\rho u^2+P(\rho ))w_y-\varepsilon \rho ^{\alpha }w_yu_y\big )\textrm{d}y\Big )\, \textrm{d}x\textrm{d}t \bigg |\nonumber \\&\leqq C +\bigg |\varepsilon \int ^T_0\int ^{r_2}_{r_1}\rho w\Big (\int ^x_{r_1}\rho ^{\alpha }w_yu_y\,\textrm{d}y\Big )\textrm{d}x\textrm{d}t\bigg |\nonumber \\&\leqq C(r_1,r_2), \end{aligned}$$

where we have used the fact that

$$\begin{aligned}&\bigg |\varepsilon \int _0^T\int _{r_1}^{r_2} \rho w\Big (\int ^x_{r_1}\rho ^{\alpha }w_yu_y\, \textrm{d}y\Big )\textrm{d}x\textrm{d}t \bigg |\nonumber \\&\leqq \varepsilon \int ^T_0\int ^{r_2}_{r_1}|\rho ^{\alpha }w_xu_x|\,\textrm{d}x\textrm{d}t\nonumber \\&\leqq \Big (\varepsilon \int ^T_0\int ^{r_2}_{r_1}\rho ^{\alpha }|u_x|^2\,\textrm{d}x \textrm{d}t\Big )^{\frac{1}{2}}\Big (\varepsilon \int ^T_0\int ^{r_2}_{r_1}\rho ^{\alpha }|w_x|^2\,\textrm{d}x\textrm{d}t\Big )^{\frac{1}{2}}\nonumber \\&\leqq C \Big (\varepsilon \int ^T_0\int ^{r_2}_{r_1}\rho ^{\alpha }\,\textrm{d}x\textrm{d}t\Big )^{\frac{1}{2}}\nonumber \\&\leqq C \Big (\varepsilon \int ^T_0\int ^{r_2}_{r_1}(\rho ^{\gamma _2}+1)\,\textrm{d}x\textrm{d}t\Big )^{\frac{1}{2}}\nonumber \\&\leqq C(r_1,r_2). \end{aligned}$$

We also have

$$\begin{aligned} \bigg |\int _0^T\int _{r_1}^{r_2} K_6\,\textrm{d}x\textrm{d}t \bigg |&\leqq C, \end{aligned}$$

$$\begin{aligned}&\bigg |\int _0^T\int _{r_1}^{r_2} K_7\,\text {d}x\text {d}t \bigg |\nonumber \\  &=\bigg |\int _0^T\int _{r_1}^{r_2} \rho (t,x) w(x)\Big (\int ^x_{r_1}\rho (t,y) (\partial _xW*\rho )(t,y)\,w(y) \,\text {d}y\Big )\text {d}x\text {d}t \bigg |\nonumber \\  &= \bigg |\int _0^T\int _{r_1}^{r_2} \rho (t,x) w(x) \Big (\int ^x_{r_1}\rho (t,y) \big (\int ^{b^+(t)}_{b^-(t)}(1-2H(x-z)+x-z)\rho (z)\,\text {d}z\big )w(y)\,\text {d}y\Big )\text {d}x\text {d}t \bigg |\nonumber \\  &\leqq C, \nonumber \end{aligned}$$

and

5.31 $$\begin{aligned}&\bigg |\int _0^T\int _{r_1}^{r_2} K_8\,\textrm{d}x\textrm{d}t \bigg |\nonumber \\&=\bigg |\int ^T_0\int ^{r_2}_{r_1}\rho (t,x)w(x) \int ^x_{r_1}\rho (t,y)\Big (\int _{b^{-}(t)}^{b^+(t)}\varpi (y-z)(u(z)-u(y))\rho (t,z)\,\textrm{d}z\Big )w(y)\,\textrm{d}y\textrm{d}x\textrm{d}t\bigg |\nonumber \\&\leqq C. \end{aligned}$$

Integrating (5.28) over $[0,T]\times [r_1, r_2]$ and utilizing (5.30)–(5.31), we can obtain (5.25). This completes the proof. $\square$

#### Corollary 5.8

Under the assumptions of Lemma 5.6, it follows from Lemma 5.7 and (5.3) that

$$\begin{aligned} \int _{b^-(t)}^{b^+(t)}\rho ^{\gamma _2+1}(t,x)\,\textrm{d}x \leqq C \int _{b^-(t)}^{b^+(t)}(\rho +\rho P(\rho ))(t,x)\,\textrm{d}x\leqq C(r_1,r_2) \qquad \text { for }t\geqq 0. \end{aligned}$$

### A special entropy pair

Compared with the polytropic gas case in [13], there is no explicit formula of the entropy kernel for the general pressure law (1.2)–(1.4) so that we have to analyze the entropy equation (2.3) carefully to obtain several desired estimates. In order to obtain the higher integrability of the velocity, we use a special entropy pair constructed in [14] such that $\rho |u|^3$ can be controlled by the entropy flux. Indeed, such a special entropy $\hat{\eta }(\rho ,u)$ is constructed as

$$\begin{aligned} \hat{\eta }(\rho ,u)={\left\{ \begin{array}{ll} \frac{1}{2}\rho u^2+\rho e(\rho )\quad & \text {for }u\ge k(\rho ),\\ -\frac{1}{2}\rho u^2-\rho e(\rho )\,\,\, & \text {for }u\le -k(\rho ), \end{array}\right. } \end{aligned}$$

for $k(\rho )=\int _{0}^{\rho }\frac{\sqrt{P'(y)}}{y}\,\textrm{d}y$ and, in the intermediate region $-k(\rho )\le u\le k(\rho )$, $\hat{\eta }(\rho ,u)$ is the unique solution of the Goursat problem of the entropy equation (2.3):

5.32 $$\begin{aligned} \left\{ \begin{aligned} \displaystyle&\eta _{\rho \rho }-k'(\rho )^2\eta _{uu}=0 \qquad \text{ for } -k(\rho )\le u\le k(\rho ),\\ \displaystyle&\eta (\rho ,u)\vert _{u=\pm k(\rho )}=\pm \big (\frac{1}{2}\rho u^2+\rho e(\rho )\big )\mathrm{.} \end{aligned} \right. \end{aligned}$$

Let us recall the following lemma, which will be used in the proof of Lemma 5.10:

#### Lemma 5.9

([14], Lemma 4.1) The Goursat problem (5.32) admits a unique solution $\hat{\eta }\in C^2(\mathbb {R}_{+} \times \mathbb {R})$ such that

(i) $|\hat{\eta }(\rho ,u)|\leqq C(\rho |u|^2+\rho ^{\gamma (\rho )})$ for $(\rho ,u)\in \mathbb {R}_{+}\times \mathbb {R}$, where $\gamma (\rho )=\gamma _1$ if $\rho \in [0,\rho _{*}]$ and $\gamma (\rho )=\gamma _2$ if $\rho \in (\rho _{*},\infty )$.
(ii) If $\hat{\eta }$ is regarded as a function of $(\rho , u)$,
  $$\begin{aligned} \qquad |\hat{\eta }_{\rho }(\rho ,u)|\leqq C(|u|^2+\rho ^{2\theta (\rho )}),\quad |\hat{\eta }_{u}(\rho ,u)|\leqq C(\rho |u|+\rho ^{\theta (\rho )+1}) \end{aligned}$$
  for $(\rho ,u)\in \mathbb {R}_{+}\times \mathbb {R}$ and, if $\hat{\eta }$ is regarded as a function of $(\rho , m)$,
  $$\begin{aligned} \qquad |\hat{\eta }_{\rho }(\rho ,m)|\leqq C(|u|^2+\rho ^{2\theta (\rho )}),\quad |\hat{\eta }_{m}(\rho ,m)|\leqq C(|u|+\rho ^{\theta (\rho )}) \end{aligned}$$
  for $$(\rho ,m)\in \mathbb {R}_{+}\times \mathbb {R}$$, where $$\theta (\rho ):=\frac{\gamma (\rho )-1}{2}$$.
(iii) If $\hat{\eta }_{m}$ is regarded as a function of $(\rho ,u)$,
  $$\begin{aligned} \qquad |\hat{\eta }_{m\rho }(\rho ,u)|\leqq C\rho ^{\theta (\rho )-1},\quad |\hat{\eta }_{mu}(\rho ,u)|\leqq C, \end{aligned}$$
  and, if $\hat{\eta }_{m}$ is regarded as a function of $(\rho ,m)$,
  $$ |\hat{\eta }_{m\rho }(\rho ,m)|\leqq C\rho ^{\theta (\rho )-1},\quad |\hat{\eta }_{mm}(\rho ,m)|\leqq C\rho ^{-1}. $$
(iv) If $\hat{q}$ is the corresponding entropy flux determined by (2.1), then $\hat{q}\in C^2(\mathbb {R}_{+}\times \mathbb {R})$ and
  $$\begin{aligned}&\hat{q}(\rho ,u)=\frac{1}{2}\rho |u|^3\pm \rho u(e(\rho )+\rho e'(\rho ))\qquad  &   \text{ for } \pm u\geqq k(\rho ),\\  &|\hat{q}(\rho ,u)|\leqq C\rho ^{\gamma (\rho )+\theta (\rho )}\qquad \qquad \qquad  &   \text{ for } |u|<k(\rho ),\\  &\hat{q}(\rho ,u)\geqq \frac{1}{2}\rho |u|^3\qquad \qquad \qquad \qquad \qquad  &   \text{ for } |u|\geqq k(\rho ),\\  &|\hat{q}-u\hat{\eta }|\leqq C(\rho ^{\gamma (\rho )}|u|+\rho ^{\gamma (\rho )+\theta (\rho )})\qquad  &   \text{ for } (\rho ,u)\in \mathbb {R}_{+}\times \mathbb {R}. \end{aligned}$$

We are now ready to prove the better integrability of the velocity.

#### Lemma 5.10

(Higher integrability of the velocity) Let $(\rho ,u)$ be the smooth solution of (1.9) and (2.10)–(2.13). Then, under the assumption of Lemma 5.6,

$$\begin{aligned} \int _0^T\int _{r_1}^{r_2} \rho |u|^3(t,x) \,\textrm{d}x \textrm{d}t\leqq C(r_1,r_2) \end{aligned}$$

for any $$(r_1,r_2)\Subset [b^-(t), b^+(t)]$$.

#### Proof

Multiplying (1.9)$_{1}$ by $\hat{\eta }_\rho$ and (1.9)$_{2}$ by $\hat{\eta }_m$, we have

5.33 $$\begin{aligned}&\hat{\eta }_t+\hat{q}_x = \hat{\eta }_m\, \Big (\varepsilon (\rho ^{\alpha } u_x)_x+\lambda \rho u+\rho V-\rho \partial _xW*\rho \Big ). \end{aligned}$$

A direct calculation shows that

5.34 $$\begin{aligned} \frac{\textrm{d}}{\textrm{d}t}\int _x^{b^+(t)}\hat{\eta }\,\textrm{d}y&=\hat{\eta }(t,b^+(t)) \frac{\textrm{d}}{\textrm{d}t}b^+(t)+\int _x^{b^+(t)}\hat{\eta }_t(t,y)\,\textrm{d}y\nonumber \\&= (u\hat{\eta })(t, b^+(t))+\int _x^{b^+(t)} \hat{\eta }_t(t,y)\,\textrm{d}y. \end{aligned}$$

Integrating (5.33) over $[x,b^+(t))$, we have

5.35 $$\begin{aligned} \hat{q}(t,x)&=\Big ( \int _x^{b^+(t)}\hat{\eta }(t,y)\,\textrm{d}y\Big )_t+\big (\hat{q}-u\hat{\eta }\big )(t,b^+(t))\nonumber \\&\quad -\varepsilon \int _x^{b^+(t)} \hat{\eta }_m(\rho ^{\alpha } u_y)_y\,\textrm{d}y- \lambda \int _x^{b^+(t)} \hat{\eta }_m\rho u\,\textrm{d}y+\int _x^{b^+(t)}\hat{ \eta }_m \rho \partial W*\rho \,\textrm{d}y\nonumber \\&\quad -\int ^{b^+(t)}_x\hat{\eta }_m\rho V\,\textrm{d}y :=\sum ^6_{i=1}I_i. \end{aligned}$$

We now estimate each term $I_i, i=1,\ldots , 6$, in (5.35). First, for $I_2$ involving the trace estimates in (5.35), it follows from [13, Lemma 3.6] and Lemma 5.9 that

5.36 $$\begin{aligned} \int _0^T\int ^{r_2}_{r_1} \big |(\hat{q}-u\hat{\eta })(t,b^+(t))\big |\, \textrm{d}x\textrm{d}t&\leqq C(r_1,r_2)\int _0^T\big (\rho ^{\gamma _1+\theta _1}(t,b^+(t)) \nonumber \\&\qquad \qquad \quad \qquad \quad \quad \,\,\, +(\rho ^{\gamma _1}|u|)(t, b^+(t))\big )\,\textrm{d}t. \end{aligned}$$

It follows from (5.6) that

5.37 $$\begin{aligned}&\int _0^T\big (\rho (t,b^+(t))^{\gamma _1+\theta _1}\,\textrm{d}t=\int ^T_0(\rho _0(b))^{\gamma _1+\theta _1}\,\textrm{d}t\leqq \rho ^{\gamma _1+\theta _1}_{*}T\leqq C. \end{aligned}$$

It is noted from (5.5), (5.6), and (5.9) that

5.38 $$\begin{aligned}&\int _0^T\int ^{r_2}_{r_1} \big (\rho ^{\gamma _1}|u|\big )(t,b^+(t))\, \text {d}x\text {d}t\nonumber \\  &=\int ^T_0\rho ^{\frac{\gamma _1-\alpha }{2}}(t,b^+(t))|(\rho ^{\frac{\gamma _1+\alpha }{2}}u)(t,b^+(t))|\,\text {d}t\nonumber \\  &\leqq (\rho _0(b))^{\frac{\gamma _1-\alpha }{2}}\int ^T_0\bigg \{\Big (\int ^{b^+(t)}_{b^-(t)}P'(\rho )\rho ^{\alpha -2}\rho ^2_x\,\text {d}x\Big )^{\frac{1}{2}}+\Big (\int ^{b^+(t)}_{b^-(t)}\rho ^{\alpha }u^2_x\,\text {d}x\Big )^{\frac{1}{2}}+\varepsilon ^{-1}b^{-\frac{1}{2}}\bigg \}\,\text {d}t\nonumber \\  &\leqq C (\rho _0(b))^{\frac{\gamma _1-\alpha }{2}} \bigg \{\varepsilon ^{-\frac{1}{2}}b^{-\frac{1}{2}}T+\Big (\varepsilon \int ^T_0\int ^{b^+(t)}_{b^-(t)}P'(\rho )\rho ^{\alpha -2}\rho ^2_x\,\text {d}x\text {d}t\Big )^{\frac{1}{2}}\Big (\frac{T}{\varepsilon }\Big )^{\frac{1}{2}}\nonumber \\  &\qquad \qquad \qquad \qquad \qquad \,+\Big (\varepsilon \int ^T_0\int ^{b^+(t)}_{b^-(t)}\rho ^{\alpha }u^2_x\,\text {d}x\text {d}t\Big )^{\frac{1}{2}}\Big (\frac{T}{\varepsilon }\Big )^{\frac{1}{2}}\bigg \}\nonumber \\  &\leqq C (\rho _0(b))^{\frac{\gamma _1-\alpha }{2}} \big (\varepsilon ^{-1}b^{-\frac{1}{2}}+\varepsilon ^{-\frac{1}{2}}\big ) \leqq C b^{-\frac{\gamma _1-\alpha }{2\gamma _1}} \big (\varepsilon ^{-1}b^{-\frac{1}{2}}+\varepsilon ^{-\frac{1}{2}}\big )\nonumber \\  &\leqq C \varepsilon ^{\frac{\gamma _1-\alpha }{2\gamma _1}p-\frac{1}{2}} \leqq C,\end{aligned}$$

where $p>\frac{\gamma _1}{\gamma _1-\alpha }.$ Substituting (5.37)–(5.38) into (5.36), we obtain

5.39 $$\begin{aligned}&\Big |\int ^T_0\int ^{r_2}_{r_1}I_2\,\textrm{d}x\textrm{d}t\Big |=\Big |\int ^T_0\int ^{r_2}_{r_1} (\hat{q}-u\hat{\eta })(t,b^+(t))\, \textrm{d}x\textrm{d}t\Big |\leqq C(r_1,r_2). \end{aligned}$$

For $I_1$, using Lemma 5.9, we have

5.40 $$\begin{aligned} \Big |\int ^T_0\int ^{r_2}_{r_1}I_1\,\textrm{d}x\textrm{d}t\Big |&=\Big |\int _0^T\int _{r_1}^{r_2}\Big (\int _x^{b^+(t)}\hat{\eta }(\rho ,\rho u)\, \textrm{d}y\Big )_t\,\textrm{d}x\textrm{d}t\Big | \nonumber \\&\leqq \Big |\int _{r_1}^{r_2}\int _{b^-(t)}^{b^+(t)}\hat{\eta }(\rho ,\rho u)(T,y)\, \textrm{d}y\textrm{d}x\Big | \nonumber \\&\quad \ +\Big |\int _{r_1}^{r_2}\int _{b^-(t)}^{b^+(t)}\hat{\eta }(\rho _0,\rho _0 u_0) \,\textrm{d}y\textrm{d}x\Big | \leqq C(r_1,r_2). \end{aligned}$$

For $I_3,$ we integrate by parts to obtain

5.41 $$\begin{aligned} -\varepsilon \int _x^{b^+(t)}\hat{\eta }_m(\rho ^{\alpha } u_y)_y\,\textrm{d}y&=-\varepsilon \Big (\hat{\eta }_m(t,b^+(t))\, (\rho ^{\alpha } u_x)(t,b^+(t))-\hat{\eta }_m(t,x)(\rho ^{\alpha } u_x)(t,x) \Big ) \nonumber \\&\quad \,\,+\varepsilon \int _x^{b^+(t)}\rho ^{\alpha } u_y(\hat{\eta }_{mu}u_y+\hat{\eta }_{m\rho }\rho _y)\,\textrm{d}y\nonumber \\&:= J_1+J_2. \end{aligned}$$

We first estimate $J_2$:

$$\begin{aligned} |J_2|=&\Big |\varepsilon \int ^{b^+(t)}_x\Big (\hat{\eta }_{m\rho }\rho ^{\alpha }u_y\rho _y+\hat{\eta }_{mu}\rho ^{\alpha }u^2_{y}\Big )\,\textrm{d}y\Big |\nonumber \\&\leqq C\varepsilon \Big |\int ^{b^+(t)}_x\rho ^{\theta (\rho )+\alpha -1}u_y\rho _y\,\textrm{d}y\Big |+\varepsilon \int ^{b^+(t)}_x\rho ^{\alpha }u^2_{y}\,\textrm{d}y\nonumber \\&\leqq \varepsilon \int ^{b^+(t)}_xP'(\rho )\rho ^{\alpha -2}\rho ^2_y\,\textrm{d}y+\varepsilon \int ^{b^+(t)}_x\frac{\rho ^{\alpha +2\theta (\rho )}}{P'(\rho )}u^2_y\,\textrm{d}y+\varepsilon \int ^{b^+(t)}_x\rho ^{\alpha }u^2_{y}\,\textrm{d}y\nonumber \\&\leqq \varepsilon \int ^{b^+(t)}_xP'(\rho )\rho ^{\alpha -2}\rho ^2_y\,\textrm{d}y+\varepsilon \int ^{b^+(t)}_x\rho ^{\alpha }u^2_{y}\,\textrm{d}y, \end{aligned}$$

where we have used the fact that $|\hat{\eta }_{mu}|\leqq C$, $|\hat{\eta }_{m\rho }|\leqq C\rho ^{\theta (\rho )-1}$, and

$$\begin{aligned}&\Big |\varepsilon \int ^{b^+(t)}_x\frac{\rho ^{\alpha +2\theta (\rho )}}{P'(\rho )}u^2_y\,\textrm{d}y\Big |\nonumber \\&\leqq \varepsilon \int ^{b^+(t)}_x\frac{\rho ^{\alpha +2\theta (\rho )}}{P'(\rho )}u^2_y\textbf{I}_{\{\rho \leqq \rho _{*}\}}\,\textrm{d}y+\varepsilon \int ^{b^+(t)}_x\frac{\rho ^{\alpha +2\theta (\rho )}}{P'(\rho )}u^2_y\textbf{I}_{\{\rho _{*}\leqq \rho \leqq \rho ^{*}\}}\,\textrm{d}y\nonumber \\&\quad +\varepsilon \int ^{b^+(t)}_x\frac{\rho ^{\alpha +2\theta (\rho )}}{P'(\rho )}u^2_y\textbf{I}_{\{\rho \geqq \rho ^{*}\}}\,\textrm{d}y\nonumber \\&\leqq \varepsilon C\int ^{b^+(t)}_x\rho ^{\alpha +2\theta (\rho )-(\gamma _1-1)}u^2_y\,\textrm{d}y+\varepsilon C\int ^{b^+(t)}_x\rho ^{\alpha +2\theta (\rho )-(\gamma _2-1)}u^2_y\,\textrm{d}y\nonumber \\&\leqq \varepsilon C\int ^{b^+(t)}_x\rho ^{\alpha }u^2_y\,\textrm{d}y. \end{aligned}$$

Then we have

5.42 $$\begin{aligned} \int ^T_0\int ^{r_2}_{r_1}|J_2|\,\textrm{d}x\textrm{d}t&\leqq \varepsilon \int ^T_0\int ^{r_2}_{r_1}\int ^{b^+(t)}_xP'(\rho )\rho ^{\alpha -2}\rho ^2_y\,\textrm{d}y\textrm{d}x\textrm{d}t\nonumber \\&\quad +\varepsilon \int ^T_0\int ^{r_2}_{r_1}\int ^{b^+(t)}_x\rho ^{\alpha }u^2_y\,\textrm{d}y\textrm{d}x\textrm{d}t\nonumber \\&\leqq C(r_1,r_2). \end{aligned}$$

For $J_1,$ we have

5.43 $$\begin{aligned}&\varepsilon \Big |\int ^T_0\int ^{r_2}_{r_1} \hat{\eta }_m\rho ^{\alpha }u_x\,\textrm{d}x\textrm{d}t\Big |\nonumber \\&\leqq \varepsilon \int ^T_0\int ^{r_2}_{r_1}\rho ^{\alpha }u^2_x\,\textrm{d}x\textrm{d}t +\varepsilon \int ^T_0\int ^{r_2}_{r_1}\rho ^{\alpha }\big (|u|+\rho ^{\theta (\rho )}\big )^2\,\textrm{d}x\textrm{d}t\nonumber \\&\leqq C +\varepsilon \int ^T_0\int ^{r_2}_{r_1}\rho ^{2\theta (\rho )+\alpha }\,\textrm{d}x\textrm{d}t+\varepsilon \int ^T_0\int ^{r_2}_{r_1}\rho ^{\alpha }u^2\,\textrm{d}x\textrm{d}t. \end{aligned}$$

To control (5.43), we need

5.44 $$\begin{aligned} \varepsilon \int ^T_0\int ^{r_2}_{r_1}\rho ^{2\theta (\rho )+\alpha }\,\textrm{d}x\textrm{d}t&=\varepsilon \int ^T_0\int ^{r_2}_{r_1}\rho ^{\gamma _2-1+\alpha }\,\textrm{d}x\textrm{d}t\leqq C(\mathcal {E}^{\varepsilon }_0,\mathcal {E}^{\varepsilon }_1)\int ^T_0\int ^{r_2}_{r_1}\rho ^{\frac{\gamma _2-1}{2}}\,\textrm{d}x\textrm{d}t\nonumber \\&\leqq C \int ^T_0\int ^{r_2}_{r_1}\rho ^{\gamma _2}\,\textrm{d}x\textrm{d}t+C(r_1,r_2) \leqq C(r_1,r_2). \end{aligned}$$

Then we have

5.45 $$\begin{aligned} \varepsilon \int ^T_0\int ^{r_2}_{r_1}\rho ^{\alpha }u^2\,\textrm{d}x\textrm{d}t&\leqq \Big (\int ^T_0\int ^{r_2}_{r_1}\rho |u|^3\,\textrm{d}x\textrm{d}t\Big )^{\frac{2}{3}} \,\Big (\int ^T_0\int ^{r_2}_{r_1}\varepsilon ^3\rho ^{3\alpha -2}\,\textrm{d}x\textrm{d}t\Big )^{\frac{1}{3}}\nonumber \\&\leqq \Big (\int ^T_0\int ^{r_2}_{r_1}\rho |u|^3\,\textrm{d}x\textrm{d}t\Big )^{\frac{2}{3}} \,\Big (\int ^T_0\int ^{r_2}_{r_1}\varepsilon ^3\rho ^{3\beta }\,\textrm{d}x\textrm{d}t+C(r_1,r_2)\Big )^{\frac{1}{3}}\nonumber \\&\leqq C(r_1,r_2) \Big (\int ^T_0\int ^{r_2}_{r_1}\rho |u|^3\,\textrm{d}x\textrm{d}t\Big )^{\frac{2}{3}}, \end{aligned}$$

where we have used $\alpha \geqq \frac{2}{3}.$ Inserting (5.44) and (5.45) into (5.43), we see that, for $\delta >0$,

$$\begin{aligned}&\varepsilon \Big |\int ^T_0\int ^{r_2}_{r_1} \hat{\eta }_m\rho ^{\alpha }u_x\,\textrm{d}x\textrm{d}t\Big | \leqq C(r_1,r_2) \frac{1}{\delta }+\delta \int ^T_0\int ^{r_2}_{r_1}\rho |u|^3\,\textrm{d}x\textrm{d}t. \end{aligned}$$

Using Lemma 5.9 and (2.12), we obtain

$$ |\varepsilon (\hat{\eta }_m\rho ^{\alpha }u_x)(t,b^+(t))|=|(\hat{\eta }_mP)(t,b^+(t))| \leqq C_{\gamma }\big (\rho ^{\gamma _1}|u|+\rho ^{\gamma _1+\theta _1}\big )(t,b^+(t)). $$

Similar again to the argument as in [13, 29] yields

5.46 $$\begin{aligned} \int ^T_0\int ^{r_2}_{r_1}|\varepsilon (\hat{\eta }_m\rho ^{\alpha }u_x)(t,b^+(t))|\,\textrm{d}x\textrm{d}t&\leqq C(r_1,r_2) \int ^T_0\big (\rho ^{\gamma _1}|u|+\rho ^{\gamma _1+\theta _1}\big )(t,b^+(t)))\,\textrm{d}t\nonumber \\&\leqq C(r_1,r_2). \end{aligned}$$

Combining (5.51) with (5.46), we have

5.47 $$\begin{aligned} \Big |\int ^T_0\int ^{r_2}_{r_1}J_1\,\textrm{d}x\textrm{d}t\Big |&\leqq \delta \int ^T_0\int ^{r_2}_{r_1}\rho |u|^3\,\textrm{d}x\textrm{d}t+C(r_1,r_2). \end{aligned}$$

Inserting (5.42) and (5.47) into (5.41), we obtain

5.48 $$\begin{aligned} \Big |\int ^T_0\int ^{r_2}_{r_1}I_3\,\textrm{d}x\textrm{d}t\Big |&\leqq \delta \int ^T_0\int ^{r_2}_{r_1}\rho |u|^3\,\textrm{d}x\textrm{d}t+C(r_1,r_2). \end{aligned}$$

We also obtain the estimates for $I_i, i=4,5,6$:

$$\begin{aligned} \Big |\int ^T_0\int ^{r_2}_{r_1}I_4\,\textrm{d}x\textrm{d}t\Big |&=\Big |\int ^T_0\int ^{r_2}_{r_1}\int ^{b^+(t)}_x\rho |u|\big (|u|+\rho ^{\theta (\rho )}\big ) \,\textrm{d}y\textrm{d}x\textrm{d}t\Big |\nonumber \\&=\Big |\int ^T_0\int ^{r_2}_{r_1}\rho u^2\,\textrm{d}x\textrm{d}t\Big |+\Big |\int ^T_0\int ^{r_2}_{r_1}\rho ^{\gamma _2}\,\textrm{d}x\textrm{d}t\Big | \leqq C(r_1,r_2), \end{aligned}$$

$$\begin{aligned}&\Big |\int ^T_0\int ^{r_2}_{r_1}I_5\,\textrm{d}x\textrm{d}t\Big |\nonumber \\&=\Big |\int ^T_0\int ^{r_2}_{r_1}\int ^{b^+(t)}_x\rho \hat{\eta }_m\partial _xW*\rho \,\textrm{d}y\textrm{d}x\textrm{d}t\Big |\nonumber \\&=\Big |\int ^T_0\int ^{r_2}_{r_1}\int ^{b^+(t)}_x\rho \hat{\eta }_m\Big (M-2\int ^{y}_{b^-(t)}\rho (t,z)\,\textrm{d}z+yM-\int ^{b^+(t)}_{b^{-}(t)}z\rho (z) \,\textrm{d}z\Big )\,\textrm{d}y\textrm{d}x\textrm{d}t\Big |\nonumber \\&\leqq C(r_1,r_2), \end{aligned}$$

and

5.49 $$\begin{aligned}&\Big |\int ^T_0\int ^{r_2}_{r_1}I_6\,\textrm{d}x\textrm{d}t\Big |\nonumber \\&=\Big |\int ^T_0\int ^{r_2}_{r_1}\int ^{b^+(t)}_x\hat{\eta }_m\rho V\,\textrm{d}y\,\textrm{d}x\textrm{d}t\Big |\nonumber \\&=\Big |\int ^T_0\int ^{r_2}_{r_1}\int ^{b^+(t)}_x\hat{\eta }_m\rho \Big (\int ^{b^+(t)}_{b^-(t)}\varpi (y-z)(u(y)-u(z))\rho (t,z)\,\textrm{d}z\Big )\,\textrm{d}y\,\textrm{d}x\textrm{d}t\Big |\nonumber \\&\leqq C(r_1,r_2). \end{aligned}$$

Combining estimates (5.39)–(5.40) and (5.48)–(5.49) leads to

$$ \int _{0}^{T}\int _{r_1}^{r_2}\hat{q}\,\textrm{d}x\textrm{d}t\leqq C(r_1, r_2), $$

which gives

5.50 $$\begin{aligned} \int _{0}^{T}\int _{[r_1, r_2]\cap \{x: |u|\geqq k(\rho )\}}\rho |u|^3\,\text {d}x\text {d}t&\leqq 2\int _{0}^{T}\int _{[r_1, r_2]\cap \{x: |u|\geqq k(\rho )\}}\hat{q}\,\text {d}x\text {d}t\nonumber \\  &=2\int _{0}^{T}\int _{r_1 }^{r_2}\hat{q}\,\text {d}x\text {d}t -2\int _{0}^{T}\int _{[r_1, r_2]\cap \{x: |u|<k(\rho )\}}\hat{q}\,\text {d}x\text {d}t\nonumber \\  &\leqq C(r_1, r_2) +C\int _{0}^{T}\int _{r_1}^{r_2}\big (\rho +\rho ^{\gamma _2+1}\big )\,\text {d}x\text {d}t\nonumber \\  &\leqq C(r_1, r_2) \end{aligned}$$

by using Lemma 3.1. On the other hand, we have

5.51 $$\begin{aligned} \int _{0}^{T}\int _{[r_1, r_2]\cap \{x:\,|u|\leqq k(\rho )\}}\rho |u|^3\,\textrm{d}x\textrm{d}t&\leqq C\int _{0}^{T}\int _{r_1}^{r_2}\rho ^{\gamma (\rho )+\theta (\rho )}\,\textrm{d}x\textrm{d}t \nonumber \\&\leqq C\int _{0}^{T}\int _{r_1}^{r_2}\big (\rho +\rho P(\rho )\big )\,\textrm{d}x\textrm{d}t \leqq C. \end{aligned}$$

Combining (5.50) with (5.51), we obtain that $\int _{0}^{T}\int _{r_1}^{r_2}\rho |u|^3\,\textrm{d}x\textrm{d}t\leqq C(r_1, r_2)$. This completes the proof. $\square$

### $W^{-1,p}_\textrm{loc}(\mathbb {R}_+^2)-$Compactness

In this section, we use the uniform estimates obtained in §5.3 to prove the following key lemma, which states the $W^{-1,p}_\textrm{loc}(\mathbb {R}_+^2)-$compactness of entropy dissipation measures for the approximate solution sequence.

#### Lemma 5.11

Let $\frac{2}{3}\leqq \alpha \leqq \gamma _2$, and let $(\eta ^{\psi },q^{\psi })$ be a weak entropy pair generated by $\psi \in C_0^2(\mathbb {R})$ defined in (2.4)–(2.5). Then, for the solutions $(\rho ^{\varepsilon },u^{\varepsilon })$ with $m^{\varepsilon }=\rho ^{\varepsilon }u^{\varepsilon }$ of CNSEs (1.9) and (2.10)–(2.13),

5.52 $$\begin{aligned} \eta ^{\psi }(\rho ^{\varepsilon },m^{\varepsilon })_t+q^{\psi }(\rho ^{\varepsilon },m^{\varepsilon })_x \qquad \text{ is } \text{ compact } \text{ in } W^{-1,p}_\textrm{loc}(\mathbb {R}_+\times \mathbb {R})\, \text{ for } \text{ any } p\in [1,2). \end{aligned}$$

#### Proof

To prove this lemma, we first recall the entropy pair $(\eta ^{\psi },q^{\psi })$ generated by $\psi \in C_0^2(\mathbb {R})$ (*cf.* [14]). Given any $\psi \in C_{0}^2(\mathbb {R})$, a regular weak entropy pair $(\eta ^{\psi },\,q^{\psi })$ is given by

5.53 $$\begin{aligned} \eta ^{\psi }(\rho ,u)=\int _{\mathbb {R}} \psi (s)\,\chi (\rho ,u,s)\, \textrm{d}s,\qquad q^{\psi }(\rho ,u)=\int _{\mathbb {R}}\psi (s)\,\sigma (\rho ,u,s) \,\textrm{d}s, \end{aligned}$$

and satisfies the following properties ([14]; also see [15, 16, 41, 42]): There exists a constant $C_{\psi }>0$ depending only on $\rho ^{*}$ and $\psi$ such that

(i) $$|\eta ^{\psi }(\rho ,u)|+ |q^{\psi }(\rho ,u)|\leqq C_{\psi }\rho \, $$ for all $$\rho \in [0,2\rho ^{*}]$$.
(ii) If $\eta ^{\psi }$ is regarded as a function of $(\rho ,m)$, then
  $$\begin{aligned} |\eta _{m}^{\psi }(\rho ,m)|+|\rho \eta _{mm}^{\psi }(\rho ,m)|\leqq C_{\psi },\qquad |\eta _{\rho }^{\psi }(\rho ,m)|\leqq C_{\psi }(1+\rho ^{\theta _1}). \end{aligned}$$
(iii) If $\eta _m^{\psi }$ is regarded as a function of $(\rho ,u)$, then
  $$\begin{aligned} |\eta _{mu}^{\psi }(\rho ,u)|+|\rho ^{1-\theta _1}\eta _{m\rho }^{\psi }(\rho ,u)|\leqq C_{\psi }. \end{aligned}$$

A direct computation on (1.9)$_{1}$ $\times \eta ^{\psi }_{\rho }(\rho ^{\varepsilon },m^{\varepsilon }) +(1.9)_2\times \eta ^{\psi }_m(\rho ^{\varepsilon },m^{\varepsilon })$ gives

$$\begin{aligned} \displaystyle \eta ^{\psi }(\rho ^{\varepsilon },m^{\varepsilon })_t+q^{\psi }(\rho ^{\varepsilon },m^{\varepsilon })_x =&\,\varepsilon \Big (\eta ^{\psi }_m(\rho ^{\varepsilon },m^{\varepsilon })(\rho ^{\varepsilon })^{\alpha }u_x^{\varepsilon }\Big )_x -\varepsilon \eta ^{\psi }_{mu}(\rho ^{\varepsilon },m^{\varepsilon })(\rho ^{\varepsilon })^{\alpha }(u_x^{\varepsilon })^2\nonumber \\  &\,-\varepsilon \eta ^{\psi }_{m\rho }(\rho ^{\varepsilon },m^{\varepsilon })(\rho ^{\varepsilon })^{\alpha }\rho _x^{\varepsilon }u_x^{\varepsilon }\nonumber \\  &+\eta ^{\psi }_m\big (\lambda \rho ^{\varepsilon }u^{\varepsilon }+\rho ^{\varepsilon }V-\rho ^{\varepsilon }\partial _xW*\rho ^{\varepsilon }\big ).\nonumber \end{aligned}$$

Let $K\Subset [b^-(t),b^+(t)]$ be compact. Using properties (ii)-(iii) of the weak entropy pair $(\eta ^{\psi },q^{\psi })$ and the Cauchy-Schwartz inequality, we have

$$\begin{aligned} \begin{aligned}&\displaystyle \varepsilon \int _{0}^{T}\int _{K}\big |\eta ^{\psi }_{mu}(\rho ^{\varepsilon },m^{\varepsilon })(\rho ^{\varepsilon })^{\alpha } (u_x^{\varepsilon })^2 +\eta ^{\psi }_{m\rho }(\rho ^{\varepsilon },m^{\varepsilon })(\rho ^{\varepsilon })^{\alpha }\rho _x^{\varepsilon }u_x^{\varepsilon }\big |\,\text {d}x\text {d}t\\  &\displaystyle \leqq C_{\psi }\varepsilon \int _{0}^{T}\int _{K}(\rho ^{\varepsilon })^{\alpha }(u_x^{\varepsilon })^2 \,\text {d}x\text {d}t +C_{\psi }\varepsilon \int _{0}^{T}\int _{K}(\rho ^{\varepsilon })^{\theta _2-1+\alpha }(\rho _x^{\varepsilon })^2 \,\text {d}x\text {d}t \leqq C(K), \end{aligned} \end{aligned}$$

and

$$\begin{aligned} \begin{aligned}&\displaystyle \int _{0}^{T}\int _{K}\Big |\eta ^{\psi }_m\Big (\lambda \rho ^{\varepsilon }u^{\varepsilon }+\rho ^{\varepsilon }V-\rho ^{\varepsilon }\partial _xW*\rho ^{\varepsilon }\Big )\Big | \,\text {d}x\text {d}t\\  &\displaystyle \leqq C_{\psi }\int _{0}^{T}\int _{K}\big (\rho ^{\varepsilon }(u^{\varepsilon })^2+\rho ^{\varepsilon }\big ) \,\text {d}x\text {d}t +C_{\psi }\int _{0}^{T}\int _{K}|\rho ^{\varepsilon }\partial _xW*\rho ^{\varepsilon }| \,\text {d}x\text {d}t \leqq C(K). \end{aligned} \end{aligned}$$

This implies that

$$\begin{aligned}  &   -\varepsilon \eta ^{\psi }_{mu}(\rho ^{\varepsilon },m^{\varepsilon })(\rho ^{\varepsilon })^{\alpha }(u_x^{\varepsilon })^2 -\varepsilon \eta ^{\psi }_{m\rho }(\rho ^{\varepsilon },m^{\varepsilon })(\rho ^{\varepsilon })^{\alpha }\rho _x^{\varepsilon }u_x^{\varepsilon }\\  &   \qquad +\eta ^{\psi }_m(\lambda \rho ^{\varepsilon }u^{\varepsilon }+\rho ^{\varepsilon }V-\rho ^{\varepsilon }\partial _xW*\rho ^{\varepsilon }) \end{aligned}$$

is uniformly bounded in $L^1([0,T]\times K)$, and thus it is compact in $W_\textrm{loc}^{-1,p_1}(\mathbb {R}_+^2)$, for $1<p_1<2$.

If $2\alpha \leqq \gamma _2+1,$ then we obtain that

5.54 $$\begin{aligned} \varepsilon ^{\frac{4}{3}}\int ^T_0\int _{K}(\rho ^{\varepsilon })^{2\alpha }\,\textrm{d}x\textrm{d}t\leqq C(K)\varepsilon ^{\frac{4}{3}}. \end{aligned}$$

If $2\alpha \geqq \gamma _2+1$ and $\alpha <\gamma _2,$ it yields that

5.55 $$\begin{aligned} \varepsilon ^{\frac{4}{3}}\int ^T_0\int _{K}(\rho ^{\varepsilon })^{2\alpha }\,\textrm{d}x\textrm{d}t&\leqq C(K)\varepsilon ^{\frac{1}{3}}\int ^T_0\int _K(\rho ^{\varepsilon })^{\alpha -\frac{\gamma _2-1}{2}}\,\textrm{d}x\textrm{d}t\nonumber \\&\quad +C(K)\varepsilon ^{\frac{1}{3}}\int ^T_0\int _K(\rho ^{\varepsilon })^{\gamma _2+1}\,\textrm{d}x\textrm{d}t. \end{aligned}$$

Moreover, it follows from (5.54) and (5.55) that

$$\begin{aligned} \begin{aligned}&\displaystyle \int _{0}^{t}\int _{K}\Big (\varepsilon \eta ^{\psi }_m(\rho ^{\varepsilon },m^{\varepsilon }) (\rho ^{\varepsilon })^{\alpha }u_x^{\varepsilon }\Big )^{\frac{4}{3}}\, \textrm{d}x\textrm{d}t\\&\displaystyle \leqq \int _{0}^{t}\int _{K}\varepsilon ^{\frac{4}{3}} (\rho ^{\varepsilon })^{^{\frac{4\alpha }{3}}}|u_x^{\varepsilon }|^{\frac{4}{3}}\,\textrm{d}x\textrm{d}t\\&\displaystyle \leqq C\varepsilon ^{\frac{4}{3}}\int _{0}^{t}\int _{K} (\rho ^{\varepsilon })^{\alpha }|u_x^{\varepsilon }|^2\,\textrm{d}x\textrm{d}t+C\varepsilon ^{\frac{4}{3}}\int _{0}^{t}\int _{K} (\rho ^{\varepsilon })^{2\alpha }\,\textrm{d}x\textrm{d}t\\&\displaystyle \leqq C(K)\varepsilon ^{\frac{1}{3}}\rightarrow 0\qquad \text { as }\varepsilon \rightarrow 0^+. \end{aligned} \end{aligned}$$

Then (4.28) and (4.3) yield that

5.56 $$\begin{aligned} \eta ^{\psi }(\rho ^{\varepsilon },m^{\varepsilon })_t+q^{\psi }(\rho ^{\varepsilon },m^{\varepsilon })_x \ \text{ is } \text{ compact } \text{ in } W_\textrm{loc}^{-1,p_2}(\mathbb {R}_+^2) \ \ \text{ for } \text{ some }\ p_2\in (1,2). \nonumber \\ \end{aligned}$$

which also implies that

5.57 $$\begin{aligned} \eta ^{\psi }(\rho ^{\varepsilon },m^{\varepsilon })_t+q^{\psi }(\rho ^{\varepsilon },m^{\varepsilon })_x\qquad \text {is compact in } W_{\textrm{loc}}^{-1,p}(\mathbb {R}_{+}^2)\text { with }1\leqq p\leqq p_{2}. \end{aligned}$$

On the other hand, for $\gamma _2\in (1,3),$ using property (i) of the weak entropy pair $(\eta ^{\psi }, q^{\psi })$, we have

$$\begin{aligned} \int ^T_0\int _K\big (|\eta ^{\psi }(\rho ^{\varepsilon },m^{\varepsilon })|^{\gamma _2+1}+|q^{\psi }(\rho ^{\varepsilon },m^{\varepsilon })|^2\big )\,\textrm{d}x\textrm{d}t \leqq C_{\psi }\int ^T_0\int _K(\rho ^{\varepsilon })^{\gamma _2+1}\,\textrm{d}x\textrm{d}t\leqq C(K), \end{aligned}$$

so that $\eta ^{\psi }(\rho ^{\varepsilon },m^{\varepsilon })$ and $q^{\psi }(\rho ^{\varepsilon },m^{\varepsilon })$ are uniformly bounded in $L_\textrm{loc}^{2}(\mathbb {R}_+^2)$. This yields that

5.58 $$\begin{aligned} \eta ^{\psi }(\rho ^{\varepsilon },m^{\varepsilon })_t+q^{\psi }(\rho ^{\varepsilon },m^{\varepsilon })_x \qquad \text{ is } \text{ uniformly } \text{ bounded } \text{ in } W_\textrm{loc}^{-1, 2}(\mathbb {R}_+^2). \end{aligned}$$

Then the interpolation compactness theorem (*cf.* [12, 25, 26]) indicates that, for $p_{2}>1, p_{1} \in \left( p_{2}, \infty \right]$, and $p_{0} \in \left[ p_{2}, p_{1}\right)$,

$$\begin{aligned} \big (\text {compact set of }W_{\textrm{loc}}^{-1, p_{2}}(\mathbb {R}_{+}^{2})\big ) \cap \big (\text {bounded set of }W_{\textrm{loc}}^{-1, p_{1}}(\mathbb {R}_{+}^{2})\big )\nonumber \\ \quad \quad \quad \quad \quad \quad \subset \big (\text {compact set of }W_{\textrm{loc}}^{-1, p_{0}}(\mathbb {R}_{+}^{2})\big ),\nonumber \end{aligned}$$

which is a generalization of Murat’s lemma in [39, 45]. Combining this theorem for $1<p_{2}<2$ and $p_{1}=2$ with the facts in (5.58) and (5.56), we conclude that

5.59 $$\begin{aligned} \eta ^{\psi }(\rho ^{\varepsilon },m^{\varepsilon })_t+q^{\psi }(\rho ^{\varepsilon },m^{\varepsilon })_x\qquad \text {is compact in }W_{\textrm{loc}}^{-1,p}(\mathbb {R}_{+}^2)\text { with }p_2\leqq p<2. \end{aligned}$$

Combining (5.59) with (5.57), we conclude (5.52). $\square$

### Proof of Theorem 2.3

Recall the following $L^p$ compensated compactness theorem established by Chen-Huang-Li-Wang-Wang [14]:

#### Theorem 5.12

([14], Lemma 2.2) Let $(\rho ^{\varepsilon },m^{\varepsilon })(t,x)=(\rho ^\varepsilon , \rho ^\varepsilon u^\varepsilon )(t,x)$ be a sequence of measurable functions with $\rho ^{\varepsilon }\geqq 0$ a.e. on $\mathbb {R}_{+}^2$ satisfying the following two conditions:

(i) For any $T>0$ and $K\Subset \mathbb {R}_+$, there exists $C(K)>0$ independent of $\varepsilon$ such that
  $$ \int _0^T\int _K\big ((\rho ^\varepsilon )^{\gamma _2+1}+\rho ^{\varepsilon }|u^{\varepsilon }|^3\big )\,\textrm{d}x\textrm{d}t\le C(K). $$
(ii) For any entropy pair $(\eta ^\psi ,q^\psi )$ defined in (5.53) with any smooth function $\psi (s)$ of compact support on $\mathbb {R}$,
  $$ \eta ^{\psi }(\rho ^{\varepsilon },m^{\varepsilon })_t+q^{\psi }(\rho ^{\varepsilon },m^{\varepsilon })_x \qquad \text{ is } \text{ compact } \text{ in } W_{\textrm{loc}}^{-1,1}(\mathbb {R}_{+}^{2}). $$

Then there exists a subsequence (still denoted) $(\rho ^{\varepsilon },m^{\varepsilon })(t,x)$ and a vector-valued function $(\rho ,m)(t,x)$ such that, as $\varepsilon \rightarrow 0^+$,

$$\begin{aligned} \begin{aligned}&\rho ^{\varepsilon }(t,x)\rightarrow \rho (t,x)~~ \text{ in }~L^{q_1}_\textrm{loc}(\mathbb {R}_{+}^2)\qquad \,\,\,\,\text {for }q_1\in [1,\gamma _2+1),\\&m^{\varepsilon }(t,x)\rightarrow m(t,x)~~ \text{ in }~L^{q_2}_\textrm{loc}(\mathbb {R}_{+}^2)\qquad \text {for }q_2\in [1,\frac{3(\gamma _2+1)}{\gamma _2+3}), \end{aligned} \end{aligned}$$

where $L_{\textrm{loc}}^{p}(\mathbb {R}_{+}^{2})$ represents $L^{p}([0, T] \times K)$ for any $T>0$ and compact set $K \Subset \mathbb {R}_+$.

The uniform estimates and compactness properties obtained in §5.1–§5.4 yield that the sequence of solutions $(\rho ^\varepsilon , m^\varepsilon )$ of problem (1.9) and (2.10)–(2.13) satisfies the compensated compactness framework established in [14] so that there exist both a subsequence (still denoted) $(\rho ^{\varepsilon },m^{\varepsilon })$ and a vector function $(\rho , m)$ such that

$$\begin{aligned} (\rho ^{\varepsilon },m^{\varepsilon })\rightarrow (\rho , m)\qquad \,\, a.e.\,\, (t,x)\in \mathbb {R}_+\times \mathbb {R}\,\,\, \text { as }\varepsilon \rightarrow 0^+. \end{aligned}$$

Since

$$ |m^{\varepsilon }|^{\frac{3(\gamma _2+1)}{\gamma _2+3}}\leqq C\big (\rho ^{\varepsilon }|u^{\varepsilon }|^3+(\rho ^{\varepsilon })^{\gamma _2+1}\big ), $$

it follows from Lemma 5.10 and Corollary 5.8 that

$$\begin{aligned} (\rho ^{\varepsilon },m^{\varepsilon })\rightarrow (\rho ,m)\qquad \text { in }L^{q_1}_\textrm{loc}(\mathbb {R}_+\times \mathbb {R})\times L^{q_2}_\textrm{loc}(\mathbb {R}_+\times \mathbb {R}) \end{aligned}$$

for $$q_1\in [1,\gamma _2+1)$$ and $$q_2\in [1,\frac{3(\gamma _2+1)}{\gamma _2+3}).$$

Moreover, we have

$$\begin{aligned} \eta ^{*}(\rho ^{\varepsilon },m^{\varepsilon })\rightarrow \eta ^{*}(\rho ,m)\qquad \text { in }L^1_\textrm{loc}(\mathbb {R}_+\times \mathbb {R})\quad \text { as }\varepsilon \rightarrow 0^+. \end{aligned}$$

Then it is direct to check that $(\rho ,m)$ is a finite-energy entropy solution of the Cauchy problem (1.1). Therefore, the proof of Theorem 2.3 and hence Theorem 2.2 is now completed for the general pressure case. $\square$

## Data Availability

Data sharing is not applicable to this article as no datasets were generated or analyzed during the current study.

## Appendix A: Construction of the Approximate Initial Data Sequence

We now construct the approximate initial data sequence $(\rho ^\varepsilon _0(x),\rho ^\varepsilon _0u^{\varepsilon }_0(x))$ with desired estimates, regularity, and boundary compatibility for the polytropic case. For the general pressure law case, the construction arguments are similar.

### A.1 The initial density

Let *J*(*x*) be the standard mollification function and $\displaystyle J_{\delta }(x):=\frac{1}{\delta }J(\frac{x}{\delta })$ for $\delta \in (0,1)$. For later use, we take $\delta =\varepsilon ^{\frac{1}{2}}$ and define $\tilde{\rho }_{0}^{\varepsilon }(x)$ as

A1 $$\begin{aligned} \tilde{\rho }_{0}^{\varepsilon }(x) :=\Big (\int _{\mathbb {R}} \big ((\rho _0\textbf{I}_{[-b+1,b-1]})(x-y)\big )^{\alpha -\frac{1}{2}}J_{\sqrt{\varepsilon }}(y)\,\textrm{d}y +\varepsilon e^{-x^2}\Big )^\frac{2}{2\alpha -1}, \end{aligned}$$

where we have denoted $\textbf{I}_{[-b+1,b-1]}$ to be the characteristic function of $\{x\in \mathbb {R}\,:\, -b+1\leqq |x|\leqq b-1\}$. Since $\alpha >\frac{2}{3}$, then $\tilde{\rho }_{0}^\varepsilon (x)\geqq \varepsilon ^{\frac{2}{2\alpha -1}} e^{-\frac{2}{2\alpha -1}x^2}>0$.

#### Lemma A.1

Let $q\in \{1, \gamma , 2\alpha -1\}$ and $\frac{2}{3}<\alpha \leqq 1$. Then $\tilde{\rho }_{0}^\varepsilon (x)$ satisfy the following properties: There exists $C_0>0$ independent of $\varepsilon$, but may depend on $\mathcal {E}_0, M, M_2, \gamma$, and $\alpha$, such that

A2 $$\begin{aligned}&\tilde{\rho }^{\varepsilon }_0(b)=\tilde{\rho }^{\varepsilon }_0(-b)\leqq C_0 b^{-\frac{1}{\gamma }}, \end{aligned}$$

A3 $$\begin{aligned}&\Vert \tilde{\rho }_0^\varepsilon \Vert _{L^q}\leqq C_0\big (\Vert \rho _0\Vert _{L^q}+ \varepsilon ^{\frac{2}{2\alpha -1}}\big ) \qquad \,\text{ for } \varepsilon \in (0,1], \end{aligned}$$

A4 $$\begin{aligned}&\lim _{\varepsilon \rightarrow 0+} \big (\Vert \tilde{\rho }_0^\varepsilon -\rho _0\Vert _{L^q} +\Vert (\tilde{\rho }^{\varepsilon }_0)^{\alpha -\frac{1}{2}}-(\rho _0)^{\alpha -\frac{1}{2}}\Vert _{L^{\frac{2q}{2\alpha -1}}}\big )=0, \end{aligned}$$

A5 $$\begin{aligned}&\varepsilon ^2\int _{\mathbb {R}}\Big |\big ((\tilde{\rho }^{\varepsilon }_0(x))^{\alpha -\frac{1}{2}}\big )_x\Big |^2\textrm{d}x \rightarrow 0 \qquad \text{ as } \varepsilon \rightarrow 0^+, \end{aligned}$$

A6 $$\begin{aligned}&\int _{\mathbb {R}} x^2|\tilde{\rho }^{\varepsilon }_0(x)|\,\textrm{d}x\leqq C_0\Big (\int _{\mathbb {R}} x^2|\rho _0(x)|\,\textrm{d}x+\varepsilon \Big ), \end{aligned}$$

A7 $$\begin{aligned}&\lim _{\varepsilon \rightarrow 0+} \int _{\mathbb {R}} x^2|\tilde{\rho }_0^\varepsilon (x)-\rho _0(x)|\,\textrm{d}x= 0, \end{aligned}$$

A8 $$\begin{aligned}&\int _{\mathbb {R}}\big |\tilde{\rho }^{\varepsilon }_0(x)(W*\tilde{\rho }^{\varepsilon }_0)(x)-\rho _0(x)(W*\rho _0)(x)\big |\,\textrm{d}x\rightarrow 0 \qquad \text { as }\varepsilon \rightarrow 0^+. \end{aligned}$$

A9 $$\begin{aligned}&\int _{\mathbb {R}}\tilde{\rho }^{\varepsilon }_0(x)(W*\tilde{\rho }^{\varepsilon }_0)(x) \,\textrm{d}x\leqq C_0\big (1+\varepsilon ^{\beta })\qquad \text { for }\beta :=\frac{2}{2\alpha -1}\in [2,6). \end{aligned}$$

#### Proof

We divide the proof into six steps. In the proof below, for simplicity, $C>0$ is a universal constant independent of $\varepsilon$, which may depend on $\mathcal {E}_0, M, M_2,\gamma$, and $\alpha$, and may be different at each occurrence so that $C_0>0$ can be chosen eventually, depending only on this $C>0$ for the statement in this lemma.

*Step 1.* The proofs of (A3)–(A5) are similar as in [13], so we omit them for brevity. Here, we remark that $\frac{2}{3}<\alpha \leqq 1,$ thus, $\beta :=\frac{2}{2\alpha -1}\in [2,\,6)$. Indeed, in the proof of (A5), using Young’s inequality, one has

A10 $$\begin{aligned} \varepsilon ^2\int _{\mathbb {R}}\Big |\big ((\tilde{\rho }^{\varepsilon }_0(x))^{\alpha -\frac{1}{2}}\big )_x\Big |^2\,\textrm{d}x&\leqq C\varepsilon \Big (\Vert \rho _0(x)\Vert ^{2\alpha -1}_{L^{2\alpha -1}}+1\Big ) \nonumber \\&\leqq C\varepsilon \Big (\int _{\mathbb {R}} (1+x^2)\rho _0(x)\,\textrm{d}x+\int _{\mathbb {R}}\frac{1}{(1+x^2)^{\frac{2\alpha -1}{2-2\alpha }}}\,\textrm{d}x+1\Big )\nonumber \\&\leqq C_0\varepsilon . \end{aligned}$$

*Step 2.* We first prove (A2), which is needed in the proofs of Lemmas 3.3 and 3.5 by (A1) since $\varepsilon <1$:

$$\begin{aligned} \tilde{\rho }^{\varepsilon }_0(b)=\tilde{\rho }^{\varepsilon }_0(-b)=(\varepsilon e^{-b^2})^{\frac{2}{2\alpha -1}}\leqq C b^{-\frac{1}{\gamma }}. \end{aligned}$$

For $b=\varepsilon ^{-p}$ and $0<\varepsilon <1,$ it is clear that there exists a positive constant C such that

$$ (\varepsilon e^{-\varepsilon ^{-2p}})^{\frac{2}{2\alpha -1}}\leqq C\varepsilon ^{\frac{p}{\gamma }}, $$

where *C* is independent of $\varepsilon \in (0,1]$.

*Step 3.* We are now ready to prove (A6). By Young’s inequality, we have

A11 $$\begin{aligned} \tilde{\rho }_{0}^{\varepsilon }(x)&=\Big (\int _{\mathbb {R}} \big ((\rho _0\textbf{I}_{[-b+1,b-1]})(x-y)\big )^{\alpha -\frac{1}{2}}J_{\sqrt{\varepsilon }}(y)\,\textrm{d}y +\varepsilon e^{-x^2}\Big )^\frac{2}{2\alpha -1}\nonumber \\&\leqq 2^{\frac{2}{2\alpha -1}}\bigg (\Big (\int _{\mathbb {R}} \big ((\rho _0\textbf{I}_{[-b+1,b-1]})(x-y)\big )^{\alpha -\frac{1}{2}}J_{\sqrt{\varepsilon }}(y)\,\textrm{d}y \Big )^\frac{2}{2\alpha -1} +(\varepsilon e^{-x^2})^{\frac{2}{2\alpha -1}}\bigg )\nonumber \\&\leqq 2^{\frac{2}{2\alpha -1}}\Big (\int _{\mathbb {R}} J_{\sqrt{\varepsilon }}(y)\rho _0(x-y)\,\textrm{d}y +(\varepsilon e^{-x^2})^{\frac{2}{2\alpha -1}}\Big ). \end{aligned}$$

Therefore, using (A11), we obtain

$$ x^2\tilde{\rho }_{0}^{\varepsilon }(x)\leqq Cx^2\Big (\int _{\mathbb {R}} J_{\sqrt{\varepsilon }}(y)\rho _0(x-y)\,\textrm{d}y +(\varepsilon e^{-x^2})^{\frac{2}{2\alpha -1}}\Big ), $$

so that

$$ \int _{\mathbb {R}} x^2|\tilde{\rho }_{0}^{\varepsilon }(x)|\, \textrm{d}x \leqq C\Big (\int _{\mathbb {R}}\int _{\mathbb {R}} J_{\sqrt{\varepsilon }}(y)x^2\rho _0(x-y)\,\textrm{d}y\textrm{d}x +\int _{\mathbb {R}} x^2(\varepsilon e^{-x^2})^{\frac{2}{2\alpha -1}}\,\textrm{d}x\Big ). $$

Notice that

A12 $$\begin{aligned} \int _{\mathbb {R}^2} J_{\sqrt{\varepsilon }}(y)x^2\rho _0(x-y)\,\textrm{d}y\textrm{d}x&=\int _{\mathbb {R}^2} J_{\sqrt{\varepsilon }}(x-y)x^2\rho _0(y)\,\textrm{d}y\textrm{d}x\nonumber \\&=\int _{\mathbb {R}}\Big (\int _{\mathbb {R}}x^2J_{\sqrt{\varepsilon }}(x-y) \,\textrm{d}x\Big )\rho _0(y)\,\textrm{d}y. \end{aligned}$$

For the integrand in (A12), we have

$$ \int _{\mathbb {R}}x^2J_{\sqrt{\varepsilon }}(x-y)\,\textrm{d}x=\int _{\mathbb {R}}(s+y)^2J_{\sqrt{\varepsilon }}(s)\,\textrm{d}s =\int _{\mathbb {R}}(s^2+2sy+y^2)J_{\sqrt{\varepsilon }}(s)\,\textrm{d}s. $$

Since

$$\begin{aligned} \int _{\mathbb {R}}sJ_{\sqrt{\varepsilon }}(s)\,\textrm{d}s=0,\quad \int _{\mathbb {R}}s^2J_{\sqrt{\varepsilon }}(s)\,\textrm{d}s=\int _{\mathbb {R}}s^2\frac{1}{\sqrt{\varepsilon }}J(\frac{s}{\sqrt{\varepsilon }})\,\textrm{d}s =\varepsilon \int _{\mathbb {R}}t^2J(t)\,\textrm{d}t\leqq C\varepsilon , \end{aligned}$$

then, for (A12), we have

$$ \int _{\mathbb {R}^2}J_{\sqrt{\varepsilon }}(y)x^2\rho _0(x-y)\,\textrm{d}y\textrm{d}x\leqq C\Big (\int _{\mathbb {R}}y^2\rho _0(y)\,\textrm{d}y+\varepsilon \Big ). $$

Therefore, we obtain (A6):

$$ \int _{\mathbb {R}} x^2|\tilde{\rho }^{\varepsilon }_0(x)|\,\textrm{d}x \leqq C\Big (\int _{\mathbb {R}} x^2|\rho _0(x)|\,\textrm{d}x+\varepsilon +\varepsilon ^{\frac{2}{2\alpha -1}}\Big ) \leqq C\Big (\int _{\mathbb {R}} x^2|\rho _0(x)|\,\textrm{d}x+\varepsilon \Big ). $$

*Step 4.* We now prove (A7) in three substeps.

*Step 4(i).* Since $M_2<\infty$, then $x^2 \rho _0(x)\in L^1$. By the standard properties of mollifier $J_{\sqrt{\varepsilon }}$, we have

$$ \int _{\mathbb {R}} x^2\big |\big ((\rho _0\textbf{I}_{[-b+1,b-1]})*J_{\sqrt{\varepsilon }}\big )(x)-x^2\rho _0(x)\big |\,\textrm{d}x \rightarrow 0 \qquad \text { as }\varepsilon \rightarrow 0^+. $$

*Step 4(ii).* We write

$$\begin{aligned}&x^2\big (\big ((\rho _0{\textbf {I}}_{[-b+1,b-1]})*J_{\sqrt{\varepsilon }}\big )(x)-\rho _0(x)\big )\\&=x^2\big ((\rho _0{\textbf {I}}_{[-b+1,b-1]})*J_{\sqrt{\varepsilon }}\big )(x)-\big ((x^2\rho _0{\textbf {I}}_{[-b+1,b-1]})*J_{\sqrt{\varepsilon }}\big )(x) \\  &\quad \, +\big ((x^2\rho _0{\textbf {I}}_{[-b+1,b-1]})*J_{\sqrt{\varepsilon }}\big )(x)-x^2\rho _0(x). \end{aligned}$$

Then

$$\begin{aligned}&\int _{\mathbb {R}} x^2\big |\big ((\rho _0{\textbf {I}}_{[-b+1,b-1]})*J_{\sqrt{\varepsilon }}\big )(x)-\rho _0(x)\big |\,\text {d}x\nonumber \\  &\leqq \int _{\mathbb {R}} x^2\big |\big ((\rho _0{\textbf {I}}_{[-b+1,b-1]})*J_{\sqrt{\varepsilon }}\big )(x) -\big ((x^2\rho _0{\textbf {I}}_{[-b+1,b-1]})*J_{\sqrt{\varepsilon }}\big )(x)\big |\,\text {d}x\nonumber \\  &\quad \, +\int _{\mathbb {R}} x^2\big |\big ((\rho _0{\textbf {I}}_{[-b+1,b-1]})*J_{\sqrt{\varepsilon }}\big )(x)-\rho _0(x)\big |\,\text {d}x:=I_1+I_2. \end{aligned}$$

It follows from Step 4(i) that $I_2\rightarrow 0$ as $\varepsilon \rightarrow 0^+$.

Now, we prove that $I_1\rightarrow 0$ as $\varepsilon \rightarrow 0^+$. We write

$$\begin{aligned} I_1&=\int _{\mathbb {R}}\Big |x^2\int _{\mathbb {R}}(\rho _0{\textbf {I}}_{[-b+1,b-1]})(y)J_{\sqrt{\varepsilon }}(x-y)\,\text {d}y\nonumber \\&\qquad \qquad -\int _{\mathbb {R}}y^2(\rho _0{\textbf {I}}_{[-b+1,b-1]})(y)J_{\sqrt{\varepsilon }}(x-y)\,\text {d}y\Big |\,\text {d}x\nonumber \\  &=\int _{\mathbb {R}}\Big |\int _{\mathbb {R}}(x^2-y^2)(\rho _0{\textbf {I}}_{[-b+1,b-1]})(y)J_{\sqrt{\varepsilon }}(x-y)\,\text {d}y\Big |\,\text {d}x\nonumber \\  &\leqq \int _{\mathbb {R}}\Big (\int _{\mathbb {R}}|x^2-y^2|J_{\sqrt{\varepsilon }}(x-y)\,\text {d}x\Big )|\rho _0(y)|\,\text {d}y.\end{aligned}$$

When $|y|<1,$ we have

$$\begin{aligned}&\int _{\mathbb {R}}|x^2-y^2|J_{\sqrt{\varepsilon }}(x-y)\,\textrm{d}x\nonumber \\&=\int _{|x-y|<\sqrt{\varepsilon }}|x^2-y^2|J_{\sqrt{\varepsilon }}(x-y)\,\textrm{d}x\nonumber \\&\leqq C\int _{|x-y|<\sqrt{\varepsilon }}|x-y|(|x|+|y|)J_{\sqrt{\varepsilon }}(x-y)\,\textrm{d}x\nonumber \\&\leqq C\sqrt{\varepsilon }. \end{aligned}$$

When $|y|\geqq 1,$ we have

$$\begin{aligned} \int _{\mathbb {R}}|x^2-y^2|J_{\sqrt{\varepsilon }}(x-y)\,\textrm{d}x&=|y|^2\int _{\mathbb {R}}\big |1-(\frac{x}{y})^2\big |J_{\sqrt{\varepsilon }}(x-y)\,\textrm{d}x\nonumber \\&=|y|^2\int _{\mathbb {R}}\big |1-(1+\frac{s}{y})^2\big |J_{\sqrt{\varepsilon }}(s)\,\textrm{d}s\nonumber \\&=|y|^2\int _{|s|<\sqrt{\varepsilon }}\big |1-(1+\frac{s}{y})^2\big |J_{\sqrt{\varepsilon }}(s)\,\textrm{d}s\nonumber \\&\leqq C\sqrt{\varepsilon }|y|^2. \end{aligned}$$

Since $M_2<\infty$, we conclude that

$$\begin{aligned} \int _{\mathbb {R}} x^2\big |\big ((\rho _0{\textbf {I}}_{[-b+1,b-1]})*J_{\sqrt{\varepsilon }}\big )(x)-\rho _0(x)\big |\,\text {d}x\rightarrow 0 \qquad \text{ as } \varepsilon \rightarrow 0^+. \end{aligned}$$

*Step 4(iii).* Using the properties of the mollifier, we have

$$\begin{aligned}&\big \Vert \big ((\rho _0{\textbf {I}}_{[-b+1,b-1]})^{\frac{1}{\beta }}*J_{\sqrt{\varepsilon }}+\varepsilon e^{-x^2}\big )^{\beta } -(\rho _0{\textbf {I}}_{[-b+1,b-1]})*J_{\sqrt{\varepsilon }}\big \Vert _{L^1}\nonumber \\  &\leqq \big \Vert \big ((\rho _0{\textbf {I}}_{[-b+1,b-1]})^{\frac{1}{\beta }}*J_{\sqrt{\varepsilon }}+\varepsilon e^{-x^2}\big )^{\beta } -\rho _0{\textbf {I}}_{[-b+1,b-1]}\big \Vert _{L^1}\nonumber \\  &\quad \, +\big \Vert (\rho _0{\textbf {I}}_{[-b+1,b-1]})-(\rho _0{\textbf {I}}_{[-b+1,b-1]})*J_{\sqrt{\varepsilon }}\big \Vert _{L^1} \rightarrow 0 \qquad \text{ as } \varepsilon \rightarrow 0^+.\end{aligned}$$

We now prove

$$\begin{aligned} \int _{\mathbb {R}}x^2\big |\big ((\rho _0\textbf{I}_{[-b+1,b-1]})^{\frac{1}{\beta }}*J_{\sqrt{\varepsilon }})(x) +\varepsilon e^{-x^2}\big )^{\beta }-\rho _0(x)\big |\,\textrm{d}x\rightarrow 0\qquad \text { as }\varepsilon \rightarrow 0^+. \end{aligned}$$

Notice first that

A13 $$\begin{aligned}&\int _{\mathbb {R}} x^2\big |\big (((\rho _0{\textbf {I}}_{[-b+1,b-1]})^{\frac{1}{\beta }}*J_{\sqrt{\varepsilon }})(x)+\varepsilon e^{-x^2}\big )^{\beta } -\rho _0(x)\big |\,\text {d}x\nonumber \\  &\leqq \int _{\mathbb {R}}x^2\big |\big (((\rho _0{\textbf {I}}_{[-b+1,b-1]})^{\frac{1}{\beta }}*J_{\sqrt{\varepsilon }})(x)+\varepsilon e^{-x^2}\big )^{\beta } -\big ((\rho _0{\textbf {I}}_{[-b+1,b-1]})*J_{\sqrt{\varepsilon }}\big )(x)\big |\,\text {d}x\nonumber \\  &\quad +\int _{\mathbb {R}}x^2\big |\big ((\rho _0{\textbf {I}}_{[-b+1,b-1]})*J_{\sqrt{\varepsilon }}\big )(x)-\rho _0(x)\big |\, \text {d}x \rightarrow 0\qquad \,\, \text{ as } \varepsilon \rightarrow 0^+.\end{aligned}$$

As in Step 4(ii), we can prove for the second term on the right-hand side of (A13) that

$$ \int _{\mathbb {R}} x^2\big |\big ((\rho _0\textbf{I}_{[-b+1,b-1]})*J_{\sqrt{\varepsilon }}\big )(x)-\rho _0(x)\big |\,\textrm{d}x\rightarrow 0 \qquad \text { as }\varepsilon \rightarrow 0^+. $$

For the first term in (A13), we have

$$\begin{aligned}&\big ((\rho _0\textbf{I}_{[-b+1,b-1]})^{\frac{1}{\beta }}*J_{\sqrt{\varepsilon }}+\varepsilon e^{-x^2}\big )^{\beta } -(\rho _0\textbf{I}_{[-b+1,b-1]})*J_{\sqrt{\varepsilon }}\nonumber \\&=\big ((\rho _0\textbf{I}_{[-b+1,b-1]})^{\frac{1}{\beta }}*J_{\sqrt{\varepsilon }}+\varepsilon e^{-x^2}\big )^{\beta } -\big ((\rho _0\textbf{I}_{[-b+1,b-1]})^{\frac{1}{\beta }}*J_{\sqrt{\varepsilon }}\big )^{\beta }\nonumber \\&\quad +\big ((\rho _0\textbf{I}_{[-b+1,b-1]})^{\frac{1}{\beta }}*J_{\sqrt{\varepsilon }}\big )^{\beta } -(\rho _0\textbf{I}_{[-b+1,b-1]})*J_{\sqrt{\varepsilon }}. \end{aligned}$$

Notice that

$$\begin{aligned}&\Big |\big ((\rho _0\textbf{I}_{[-b+1,b-1]})^{\frac{1}{\beta }}*J_{\sqrt{\varepsilon }}+\varepsilon e^{-x^2}\big )^{\beta } -\big ((\rho _0\textbf{I}_{[-b+1,b-1]})^{\frac{1}{\beta }}*J_{\sqrt{\varepsilon }}\big )^{\beta }\Big |\nonumber \\&\leqq \beta \varepsilon e^{-x^2}\big ((\rho _0\textbf{I}_{[-b+1,b-1]})^\frac{1}{\beta }*J_{\sqrt{\varepsilon }}+\varepsilon e^{-x^2}\big )^{\beta -1}, \end{aligned}$$

which leads directly to

$$\begin{aligned}&\lim _{\varepsilon \rightarrow 0+} \int _{\mathbb {R}} x^2\big |\big (((\rho _0{\textbf {I}}_{[-b+1,b-1]})^{\frac{1}{\beta }}*J_{\sqrt{\varepsilon }})(x)+\varepsilon e^{-x^2}\big )^{\beta }\nonumber \\  &\quad \qquad \qquad \qquad \qquad -\big (((\rho _0{\textbf {I}}_{[-b+1,b-1]})^{\frac{1}{\beta }}*J_{\sqrt{\varepsilon }})(x)\big )^{\beta }\big |\,\text {d}x=0. \end{aligned}$$

Thus, it remains to prove

$$ \lim _{\varepsilon \rightarrow 0+} \int _{\mathbb {R}} x^2\big |\big (((\rho _0\textbf{I}_{[-b+1,b-1]})^{\frac{1}{\beta }}*J_{\sqrt{\varepsilon }})(x)\big )^{\beta } -\rho _0(x)\big |\,\textrm{d}x=0. $$

Since $M_2<\infty$, then, for any $\varepsilon >0$, there exists $N>1$ such that

$$\begin{aligned} \int _{|x|>N-1}x^2\rho _0(x)\,\textrm{d}x<\varepsilon . \end{aligned}$$

Using the Hölder inequality and the fact that $\int _{\mathbb {R}}J_{\sqrt{\varepsilon }}(y)\,\textrm{d}y=1$ for $0<\varepsilon <1$, we have

$$\begin{aligned}&\int _{|x|>N}x^2\Big (\int _{\mathbb {R}}(\rho _0\textbf{I}_{[-b+1,b-1]})^{\frac{1}{\beta }}(x-y)J_{\sqrt{\varepsilon }}(y)\,\textrm{d}y\Big )^{\beta }\,\textrm{d}x\nonumber \\&\leqq \int _{|x|>N}x^2\int _{\mathbb {R}}\rho _0(x-y)J_{\sqrt{\varepsilon }}(y)\,\textrm{d}y\textrm{d}x=\int _{\mathbb {R}}\Big (\int _{|x|>N}x^2\rho _0(x-y)\,\textrm{d}x\Big )J_{\sqrt{\varepsilon }}(y)\,\textrm{d}y\nonumber \\&=\int _{\mathbb {R}}\Big (\int _{|y+\xi |>N}(y^2+\xi ^2+2y\xi )\rho _0(\xi )\,\textrm{d}\xi \Big )J_{\sqrt{\varepsilon }}(y)\,\textrm{d}y\nonumber \\&\leqq 2\int _{|y|<\sqrt{\varepsilon }}\Big (\int _{|y+\xi |>N}(\varepsilon ^2+\xi ^2)\rho _0(\xi )\,\textrm{d}\xi \Big )J_{\sqrt{\varepsilon }}(y)\,\textrm{d}y< 2(\varepsilon ^2+\varepsilon )\rightarrow 0 \quad \text {as }\varepsilon \rightarrow 0^+.\nonumber \end{aligned}$$

Therefore, it suffices to show

A14 $$\begin{aligned} \lim _{\varepsilon \rightarrow 0+}\int _{|x|\leqq N}x^2\Big |\Big (\int _{\mathbb {R}}(\rho _0\textbf{I}_{[-b+1,b-1]})^{\frac{1}{\beta }}(x-y)J_{\sqrt{\varepsilon }}(y)\,\textrm{d}y\Big )^{\beta }-\rho _0(x)\Big |\,\textrm{d}x=0. \end{aligned}$$

Note that

$$\begin{aligned}&\int _{|x|\leqq N}x^2\Big |\Big (\int _{\mathbb {R}}(\rho _0\textbf{I}_{[-b+1,b-1]})^{\frac{1}{\beta }}(x-y)J_{\sqrt{\varepsilon }}(y)\,\textrm{d}y\Big )^{\beta }-\rho _0(x)\Big |\,\textrm{d}x\nonumber \\&\leqq N^2\int _{|x|<N} \Big |\Big (\int _{|y|<\sqrt{\varepsilon }}\rho _0^{\frac{1}{\beta }}(x-y)J_{\sqrt{\varepsilon }}(y)\,\textrm{d}y\ \Big )^{\beta }-\rho _0(x)\Big |\,\textrm{d}x. \end{aligned}$$

Using Hölder’s inequality, we have

$$\begin{aligned} \Big (\int _{|y|<\sqrt{\varepsilon }}\rho _0^{\frac{1}{\beta }}(x-y)J_{\sqrt{\varepsilon }}(y)\,\textrm{d}y\Big )^{\beta }&\leqq \int _{|y|<\sqrt{\varepsilon }}\rho _0(x-y)J_{\sqrt{\varepsilon }}(y)\,\textrm{d}y\nonumber \\&\leqq \sup _{0<\varepsilon<1}\int _{|y|<\sqrt{\varepsilon }}\rho _0(x-y)J_{\sqrt{\varepsilon }}(y)\,\textrm{d}y:=\Phi ^{*}\rho _0(x). \end{aligned}$$

Notice that

$$ \Phi ^{*}\rho _0(x)\leqq C\Vert J\Vert _{L^1}\mathcal {M}\rho _0(x), $$

where $\mathcal {M}$ is the Hardy-Littlewood maximal operator that is of strong (*p*, *p*) type for $1<p<\infty$. Then, using Lebesgue’s dominated convergence theorem and

$$ \lim _{\varepsilon \rightarrow 0^+}\big ((\rho _0{\textbf {I}}_{[-b+1,b-1]})^{\frac{1}{\beta }}*J_{\sqrt{\varepsilon }}\big )(x) =\rho _0^{\frac{1}{\beta }}(x), $$

we conclude (A14), which implies (A7).

*Step 5.* We now prove (A8). We first note that

$$\begin{aligned}&\int _{\mathbb {R}}\Big |\tilde{\rho }^{\varepsilon }_0(x)(W*\tilde{\rho }^{\varepsilon }_0)(x)-\rho _0(x)(W*\rho _0)(x)\Big |\,\textrm{d}x\nonumber \\&\leqq \int _{\mathbb {R}}|\tilde{\rho }^{\varepsilon }_0(x)-\rho _0(x)||(W*\tilde{\rho }^{\varepsilon }_0)(x)|\,\textrm{d}x +\int _{\mathbb {R}}\rho _0(x)|(W*(\tilde{\rho }_0-\rho ))(x)|\,\textrm{d}x:=I+II. \end{aligned}$$

Since

$$\begin{aligned}&|(W*\tilde{\rho }^{\varepsilon }_0)(x)|=\Big |\int _{\mathbb {R}}W(x-y)\tilde{\rho }^{\varepsilon }_0(x)\,\textrm{d}x\Big | \leqq \int _{\mathbb {R}}\big (x^2+y^2+\frac{1}{2}\big )\tilde{\rho }^{\varepsilon }_0(y)\,\textrm{d}y,\\&\tilde{\rho }^{\varepsilon }_0(x)=\Big (\int _{\mathbb {R}}((\rho _0\textbf{I}_{[-b+1,b-1]})(x-\bar{y}))^{\alpha -\frac{1}{2}}J_{\sqrt{\varepsilon }}(\bar{y})\,\textrm{d}\bar{y} +\varepsilon e^{-x^2}\Big )^{\beta }\nonumber \\&\qquad \,\,\leqq C\Big (\int _{\mathbb {R}}(\rho _0\textbf{I}_{[-b+1,b-1]})^{\frac{1}{\beta }}(x-\bar{y})J_{\sqrt{\varepsilon }}(\bar{y})\,\textrm{d}\bar{y}\Big )^{\beta } +C\big (\varepsilon e^{-x^2}\big )^{\beta }, \end{aligned}$$

we have

A15 $$\begin{aligned}&\int _{\mathbb {R}}\left( x^2+y^2+\frac{1}{2}\right) \tilde{\rho }^{\varepsilon }_0(y)\,\text {d}y\nonumber \\  &=\left( x^2+\frac{1}{2}\right) \int _{\mathbb {R}}\tilde{\rho }^{\varepsilon }_0(y)\,\text {d}y+\int _{\mathbb {R}}y^2\tilde{\rho }^{\varepsilon }_0(y)\,\text {d}y\nonumber \\  &\leqq C\left( x^2+\frac{1}{2}\right) \Big (\int _{\mathbb {R}}\Big (\int _{\mathbb {R}}(\rho _0{\textbf {I}}_{[-b+1,b-1]})^{\frac{1}{\beta }}(y-\bar{y})J_{\sqrt{\varepsilon }}(\bar{y})\,\text {d}\bar{y}\Big )^{\beta }\,\text {d}y+\int _{\mathbb {R}}(\varepsilon e^{-y^2})^{\beta }\,\text {d}y\Big )\nonumber \\  &\quad \, + C\int _{\mathbb {R}}\Big (\int _{\mathbb {R}}|y|^{\frac{2}{\beta }}(\rho _0{\textbf {I}}_{[-b+1,b-1]})^{\frac{1}{\beta }}(y-\bar{y})J_{\sqrt{\varepsilon }}(\bar{y})\,\text {d}\bar{y}\Big )^{\beta }\,\text {d}y+ C\int _{\mathbb {R}}y^2(\varepsilon e^{-y^2})^{\beta }\,\text {d}y\nonumber \\  &\leqq C\left( x^2+\frac{1}{2}\right) \Big (\int _{\mathbb {R}}\Big (\int _{\mathbb {R}}\rho _0(y-\bar{y})(J_{\sqrt{\varepsilon }}(\bar{y}))^{\beta }\,\text {d}y\Big )^{\frac{1}{\beta }}\,\text {d}\bar{y}\Big )^{\beta }+\varepsilon ^{\beta }\Big )\nonumber \\  &\quad \, + C\Big (\int _{\mathbb {R}}\Big (\int _{\mathbb {R}}|y|^2\rho _0(y-\bar{y})(J_{\sqrt{\varepsilon }}(\bar{y}))^{\beta }\,\text {d}y\Big )^{\frac{1}{\beta }}\,\text {d}\bar{y}\Big )^{\beta }+C \varepsilon ^{\beta }\nonumber \\  &\leqq CM^{\beta }(x^2+\frac{1}{2})+C\varepsilon ^{\beta }(x^2+\frac{1}{2})+C\int _{\mathbb {R}}\Big (|\bar{y}|^{\frac{2}{\beta }}M+M_2\Big )J_{\sqrt{\varepsilon }}(\bar{y})\,\text {d}\bar{y}\nonumber \\  &\leqq C(x^2+1). \end{aligned}$$

Using (A15), we have

A16 $$\begin{aligned} I&=\int _{\mathbb {R}}|\tilde{\rho }^{\varepsilon }_0(x)-\rho _0(x)||(W*\tilde{\rho }^{\varepsilon }_0)(x)|\,\textrm{d}x\nonumber \\&\leqq C\int _{\mathbb {R}}(x^2+1)|\tilde{\rho }^{\varepsilon }_0(x)-\rho _0(x)|\,\textrm{d}x\rightarrow 0\qquad \text { as }\varepsilon \rightarrow 0^+. \end{aligned}$$

Since

$$\begin{aligned} \big |(W*(\tilde{\rho }^{\varepsilon }_0(x)-\rho _0))(x)\big |&=\Big |\int _{\mathbb {R}}W(x-y)(\tilde{\rho }^{\varepsilon }_0(y)-\rho _0(y))\,\textrm{d}y\Big |\nonumber \\&\leqq \int _{\mathbb {R}}(x^2+y^2+1)|\tilde{\rho }^{\varepsilon }_0(y)-\rho _0(y)|\,\textrm{d}y\nonumber \\&=(x^2+1)\int _{\mathbb {R}}|\tilde{\rho }^{\varepsilon }_0(y)-\rho _0(y)|\,\textrm{d}y+\int _{\mathbb {R}}y^2|\tilde{\rho }^{\varepsilon }_0(y)-\rho _0(y)|\,\textrm{d}y, \end{aligned}$$

then

A17 $$\begin{aligned} II&=\int _{\mathbb {R}}\rho _0(x)\big |(W*(\tilde{\rho }_0-\rho ))(x)\big |\,\text {d}x\nonumber \\  &\leqq \int _{\mathbb {R}}(x^2+1)\rho _0(x)\,\text {d}x\int _{\mathbb {R}}|\tilde{\rho }^{\varepsilon }_0(y)-\rho _0(y)|\,\text {d}y+\int _{\mathbb {R}}\rho _0(x)\,\text {d}x \nonumber \\  &\quad \, + \int _{\mathbb {R}}y^2|\tilde{\rho }^{\varepsilon }_0(y)-\rho _0(y)|\,\text {d}y\nonumber \\  &=(M_2+M)\Vert \tilde{\rho }^{\varepsilon }_0-\rho _0\Vert _{L^1}+M\Vert y^2(\tilde{\rho }^{\varepsilon }_0(y)-\rho _0(y))\Vert _{L^1}\rightarrow 0 \qquad \text{ as } \varepsilon \rightarrow 0^+. \end{aligned}$$

Combining (A16) and (A17), we conclude (A8).

*Step 6.* We now prove (A9). For $\tilde{W}:=W+\frac{1}{2}$, then $0\leqq \tilde{W}(x-y)\leqq x^2+y^2+1.$ Define

$$ \hat{\rho }_0(x):=\Big (\int _{\mathbb {R}}(\rho _0\textbf{I}_{[-b+1,b-1]})^{\alpha -\frac{1}{2}}(x-y)J_{\sqrt{\varepsilon }}(y)\,\textrm{d}y\Big )^{\beta }. $$

Then we have

A18 $$\begin{aligned} \tilde{\rho }^{\varepsilon }_0(x)\leqq C\big (\hat{\rho }_0(x)+(\varepsilon e^{-x^2})^{\beta }\big ). \end{aligned}$$

It follows from (A18) that

A19 $$\begin{aligned}&\int _{\mathbb {R}}\tilde{\rho }^{\varepsilon }_0(x)(W*\tilde{\rho }^{\varepsilon }_0)(x) \,\text {d}x \leqq \int _{\mathbb {R}}\tilde{\rho }^{\varepsilon }_0(x)(\tilde{W}*\tilde{\rho }^{\varepsilon }_0)(x) \,\text {d}x\nonumber \\  &\leqq C\int _{\mathbb {R}}(\hat{\rho }_0(x)+\varepsilon ^{\beta }e^{-\beta x^2})\Big (\int _{\mathbb {R}}\tilde{W}(x-y)\hat{\rho }_0(y)\,\text {d}y+\varepsilon ^{\beta }\int _{\mathbb {R}}\tilde{W}(x-y)e^{-\beta y^2}\,\text {d}y\Big )\,\text {d}x\nonumber \\  &=C\int _{\mathbb {R}}\hat{\rho }_0(x)\int _{\mathbb {R}}\tilde{W}(x-y)\hat{\rho }_0(y)\,\text {d}y \text {d}x+C\varepsilon ^{\beta }\int _{\mathbb {R}}\hat{\rho }_0(x)\int _{\mathbb {R}}\tilde{W}(x-y)e^{\beta y^2}\,\text {d}y \text {d}x\nonumber \\  &\quad \, +C\varepsilon ^{\beta }\int _{\mathbb {R}}e^{-\beta x^2}\int _{\mathbb {R}}\tilde{W}(x-y)\hat{\rho }_0(y)\,\text {d}y\text {d}x+C\varepsilon ^{2\beta }\int _{\mathbb {R}}e^{-\beta x^2}\int _{\mathbb {R}}\tilde{W}(x-y)e^{-\beta y^2}\,\text {d}y\text {d}x\nonumber \\  &=\sum ^4_i\mathcal {T}_i. \end{aligned}$$

Using Hölder’s inequality, we have

$$\begin{aligned} \hat{\rho }_0(x)&=\Big (\int _{\mathbb {R}}(\rho \textbf{I}_{[-b+1,b-1]})^{\alpha -\frac{1}{2}}(x-y)J^{\frac{1}{\beta }}_{\sqrt{\varepsilon }}(y)J^{\frac{1}{\beta '}}_{\sqrt{\varepsilon }}(y)\,\textrm{d}y\Big )^{\beta }\nonumber \\&\leqq \Big (\int _{\mathbb {R}}\rho _0(x-y)J_{\sqrt{\varepsilon }}(y)\,\textrm{d}y\Big )\Big (\int _{\mathbb {R}}J_{\sqrt{\varepsilon }}(y)\,\textrm{d}y\Big )^{\frac{\beta }{\beta '}}\leqq \int _{\mathbb {R}}\rho _0(x-y)J_{\sqrt{\varepsilon }}(y)\,\textrm{d}y, \end{aligned}$$

where we have used $\frac{1}{\beta }+\frac{1}{\beta '}=1,$ and the property of mollifier: $\int _{\mathbb {R}}J_{\sqrt{\varepsilon }}(y)\,\textrm{d}y=1.$ Then

$$ \int _{\mathbb {R}}\hat{\rho }_0(x)\,\textrm{d}x\leqq \int _{\mathbb {R}}\int _{\mathbb {R}}\rho _0(x-y)J_{\sqrt{\varepsilon }}(y)\,\textrm{d}x\textrm{d}y=M, $$

and

$$\begin{aligned} \int _{\mathbb {R}}x^2\hat{\rho }_0(x)\,\textrm{d}x&\leqq \int _{\mathbb {R}}x^2\int _{\mathbb {R}}\rho _0(x-y)J_{\sqrt{\varepsilon }}(y)\,\textrm{d}y\textrm{d}x \\&=\int _{\mathbb {R}}\left( \int _{\mathbb {R}}x^2\rho _0(x-y)\,\textrm{d}x\right) J_{\sqrt{\varepsilon }}(y)\,\textrm{d}y\nonumber \\&=\int _{\mathbb {R}}\left( \int _{\mathbb {R}}(y+\bar{x})^2\rho _0(\bar{x})\,\textrm{d}\bar{x}\right) J_{\sqrt{\varepsilon }}(y)\,\textrm{d}y\nonumber \\&\leqq 2\int _{\mathbb {R}}(y^2M+M_2)J_{\sqrt{\varepsilon }}(y)\,\textrm{d}y\leqq C, \end{aligned}$$

where we have used $\int _{\mathbb {R}}y^2J_{\sqrt{\varepsilon }}(y)\,\textrm{d}y\leqq \varepsilon .$

Now, we estimate (A19) term by term.

$$\begin{aligned} \mathcal {T}_1&\leqq C\int _{\mathbb {R}}\hat{\rho }_0(x)\int _{\mathbb {R}}(x^2+y^2+1)\hat{\rho }_0(y)\,\textrm{d}y\textrm{d}x\nonumber \\&\leqq CM\int _{\mathbb {R}}\hat{\rho }_0(x)(x^2+1)\,\textrm{d}x+C\int _{\mathbb {R}}\hat{\rho }_0(x)M_2\,\textrm{d}x\leqq C(M,M_2).\\ \mathcal {T}_2&\leqq C\varepsilon ^{\beta }\int _{\mathbb {R}}\hat{\rho }_0(x)\int _{\mathbb {R}}(x^2+y^2+1)e^{-\beta y^2}\,\textrm{d}y\textrm{d}x \\&\leqq C\varepsilon ^{\beta }\int _{\mathbb {R}}\hat{\rho }_0(x)(x^2+1)\,\textrm{d}x\leqq C\varepsilon ^{\beta }.\\ \mathcal {T}_3&\leqq C\varepsilon ^{\beta }\int _{\mathbb {R}}e^{-\beta x^2}\int _{\mathbb {R}}(x^2+y^2+1)\hat{\rho }_0(y)\,\textrm{d}y\textrm{d}x\nonumber \\&\leqq C\varepsilon ^{\beta }\int _{\mathbb {R}}e^{-\beta x^2}(x^2M+M_2+M)\,\textrm{d}x\leqq C\varepsilon ^{\beta }.\\ \mathcal {T}_4&\leqq C\varepsilon ^{2\beta }\int _{\mathbb {R}}e^{-\beta x^2}\int _{\mathbb {R}}(x^2+y^2+1)e^{-\beta x^2}\,\textrm{d}y\textrm{d}x\leqq C\varepsilon ^{2\beta }. \end{aligned}$$

Combining these estimates together, we conclude (A9). $\square$

Now we define $\bar{\rho }_{0}^{\varepsilon }(x)$ by

A20 $$\begin{aligned} \bar{\rho }_{0}^{\varepsilon }(x)&:=\tilde{\rho }_{0}^{\varepsilon }(x)\,\textbf{I}_{[-b,b]}(x). \end{aligned}$$

#### Remark A.1

We have multiplied by a cut-off function $\textbf{I}_{[-b,b]}(x)$ in (A20) such that $(W*\bar{\rho }_{0}^{\varepsilon }) (x)$ is meaningful. Otherwise, when $|y|>b$, $\bar{\rho }_{0}^{\varepsilon }(y)=b^{-\frac{1}{\gamma }}$ so that $(W*\bar{\rho }_{0}^{\varepsilon })(x)\sim \int _{|y|>b}W(x-y)\,\textrm{d}y \, b^{-\frac{1}{\gamma }}=\infty$.

Then we can obtain the following lemma:

#### Lemma A.2

Let $b=\varepsilon ^{-p}, p>\frac{\gamma }{\gamma -\alpha },$ and $q\in \{1,\gamma ,2\alpha -1\}.$ The smooth function $\bar{\rho }_{0}^{\varepsilon }(x)$ defined in (A20) satisfies the following properties: There exists $C_0>0$ independent of $\varepsilon$ but may depend on $(\mathcal {E}_0, M, M_2, \gamma ,\alpha )$ such that

$$\begin{aligned}&\bar{\rho }^{\varepsilon }_0(b)=\bar{\rho }^{\varepsilon }_0(-b)\leqq C_0 b^{-\frac{1}{\gamma }},\\&\int ^{b}_{-b} |\tilde{\rho }_0^{\varepsilon ,b}(x)-\bar{\rho }_0^\varepsilon (x)|^q\,\textrm{d}x +\int ^{b}_{-b} \Big | (\tilde{\rho }_{0}^{\varepsilon }(x))^{\alpha -\frac{1}{2}} -(\bar{\rho }_{0}^{\varepsilon }(x))^{\alpha -\frac{1}{2}}\Big |^{\frac{2q}{2\alpha -1}} \,\textrm{d}x\rightarrow 0 \quad \text{ as } \varepsilon \rightarrow 0^+,\\&\int ^b_{-b}\bar{\rho }^{\varepsilon }_0(x)\,\textrm{d}x\rightarrow \int _{\mathbb {R}}\rho _0(x)\,\textrm{d}x=M\qquad \text { as } \varepsilon \rightarrow 0^+,\\&\varepsilon ^2\int ^b_{-b}\Big |\big ((\bar{\rho }_0^{\varepsilon }(x))^{\alpha -\frac{1}{2}}\big )_x\Big |^2 \,\textrm{d}x \leqq C_0\varepsilon , \\&\int ^{b}_{-b} x^2|\tilde{\rho }_0^{\varepsilon }(x)-\bar{\rho }_0^\varepsilon (x)|\,\textrm{d}x\rightarrow 0 \qquad \,\,\text{ as } \varepsilon \rightarrow 0^+,\\&\int ^b_{-b}|\bar{\rho }^{\varepsilon }_0(x)W*\bar{\rho }^{\varepsilon }_0(x)-\tilde{\rho }^{\varepsilon }_0(x)W*\tilde{\rho }^{\varepsilon }_0(x)|\,\textrm{d}x\rightarrow 0 \qquad \,\,\text { as }\varepsilon \rightarrow 0^+. \end{aligned}$$

Since $\displaystyle \int ^b_{-b} \tilde{\rho }_{0}^{\varepsilon }(x)\,\textrm{d}x\ne M$ in general, we define

A21 $$\begin{aligned} \rho _0^{\varepsilon }(x):=\frac{M }{\int ^b_{-b} \bar{\rho }_{0}^{\varepsilon }(x)\,\textrm{d}x} \bar{\rho }_{0}^{\varepsilon }(x). \end{aligned}$$

Combining (A21) with Lemmas A.1 and A.2, we have

#### Lemma A.3

Let $q \in \{1,\gamma ,2\alpha -1\}$ and $\frac{2}{3}<\alpha \leqq 1.$ The smooth function $\rho _{0}^{\varepsilon }(x)$ defined in (A21) satisfies the following properties: There exists $C_0>0$ independent of $\varepsilon \in (0,1]$ but may depend on $(\mathcal {E}_0, M, M_2, \gamma ,\alpha )$ such that

$$\begin{aligned}&(\rho ^{\varepsilon }_0(b))^{\gamma }\, b=(\rho ^{\varepsilon }_0(-b))^{\gamma }\, b\leqq C_0,\\&\int ^b_{-b} \big |\rho _0^{\varepsilon }(x)-\rho _0(x)\big |^q\, \textrm{d}x+\int ^b_{-b} \Big | (\rho _{0}^{\varepsilon }(x))^{\alpha -\frac{1}{2}} -(\rho _{0}(x))^{\alpha -\frac{1}{2}}\Big |^{\frac{2q}{2\alpha -1}} \,\textrm{d}x\rightarrow 0 \qquad \text{ as } \varepsilon \rightarrow 0^+,\\&\varepsilon ^2\int ^b_{-b}\Big |\big ((\rho ^{\varepsilon }_0(x))^{\alpha -\frac{1}{2}}\big )_x\Big |^2 \textrm{d}x \leqq C_0\varepsilon , \\&\int ^{b}_{-b} x^2|\rho _0^{\varepsilon }(x)-\rho _0(x)|\,\textrm{d}x\rightarrow 0\qquad \text{ as } \varepsilon \rightarrow 0^+,\\&\int ^b_{-b}|\rho ^{\varepsilon }_0(x)(W*\rho ^{\varepsilon }_0)(x)-\rho _0(x)(W*\rho _0)(x)|\,\textrm{d}x\rightarrow 0 \quad \text { as }\varepsilon \rightarrow 0^+. \end{aligned}$$

### A.2 The initial velocity

Next, we construct the approximate initial data for the velocity. We first define

A22 $$\begin{aligned}&\bar{u}_{0}^{\varepsilon }(x) :=\frac{1}{\sqrt{\rho _0^{\varepsilon }(x)}}\int _{\mathbb {R}}\Big (\frac{m_0\textbf{I}_{[-b+2,b-2]}}{\sqrt{\rho _0}}\Big ) (x-y)\,J_{\sqrt{\varepsilon }}(y)\,\textrm{d}y, \end{aligned}$$

for which the following properties are valid:

#### Lemma A.4

Let $\frac{2}{3}<\alpha \leqq 1$. Then $\bar{u}^\varepsilon _0(x)$ defined in (A22) satisfies

$$\begin{aligned}&\textrm{supp}\, \bar{u}^{\varepsilon }_0(x) \subset \{x\in \mathbb {R}\, :\,-b+1\leqq x\leqq b-1\},\\&\int _{\mathbb {R}}\rho _0^\varepsilon (x) |\bar{u}_0^\varepsilon (x)|^2\,\textrm{d}x \equiv \int _{\mathbb {R}}\frac{|m_0(x)|^2}{\rho _0(x)}\,\textrm{d}x \qquad \,\,\,\text{ for } \text{ any } \varepsilon \in (0,1], \\&\lim _{\varepsilon \rightarrow 0^+} \Vert \rho ^\varepsilon _0 \bar{u}^\varepsilon _0-m_0\Vert _{L^1(\mathbb {R})}=0. \end{aligned}$$

The proof of Lemma A.4 is similar to that in [14, Lemma A.8] and [29, Lemma 5.4], so we omit it for brevity.

We still need to modify $\bar{u}_{0}^{\varepsilon }(x)$ so that it satisfies the stress-free boundary condition (2.8) at $x=\pm b$. To this end, we define a cut-off function $S=S(z)\in C^\infty (\mathbb {R})$ that satisfies

A23 $$\begin{aligned} S(z)={\left\{ \begin{array}{ll} 0\quad & \text{ if } z\in (-\infty ,0),\\ 1\quad & \text{ if } z\in (2,\infty ),\\ \text{ monotonically } \text{ increasing } & \text{ if } z\in [0,2]. \end{array}\right. } \end{aligned}$$

Then we define

A24 $$\begin{aligned} u_{0}^{\varepsilon }(x)&:=\bar{u}_{0}^{\varepsilon }(x) -\frac{1}{\varepsilon }\,S(4(x-(b-\frac{1}{2}))) \int ^b_x\frac{P(\rho ^{\varepsilon }_0(z))}{\mu (\rho ^{\varepsilon }_0(z))}\,\textrm{d}z\nonumber \\&\quad -\frac{1}{\varepsilon }\,S(-4(x-(b-\frac{1}{2}))) \int ^{-b}_{x}\frac{P(\rho ^{\varepsilon }_0(z))}{\mu (\rho ^{\varepsilon }_0(z))}\,\textrm{d}z. \end{aligned}$$

It is direct to check that $u_{0}^{\varepsilon }(x)\in C^{\infty }([-b,b])$ satisfies the following boundary conditions:

$$\begin{aligned} u_{0}^{\varepsilon }(\pm b)=0,\qquad \big (P(\rho ^{\varepsilon }_0(x))-\varepsilon \mu (\rho ^{\varepsilon }_0(x))(u^{\varepsilon }_0(x))_x\big )\big |_{x=\pm b}=0. \end{aligned}$$

A direct calculation following (A24) implies that

A25 $$\begin{aligned}&\int ^b_{-b}\Big |\sqrt{\rho _{0}^{\varepsilon }(x)}u_{0}^{\varepsilon }(x)-\sqrt{\rho _{0}^{\varepsilon }(x)}\bar{u}_{0}^{\varepsilon }(x)\Big |^2 \,\textrm{d}x\nonumber \\&\leqq \frac{C}{\varepsilon ^2}\Big (\int ^{-b+\frac{1}{2}}_{-b}\rho ^{\varepsilon }_0(x)\big |S(-4x-4b+2)\int ^x_{-b}(\rho ^{\varepsilon }_0(z))^{\gamma -\alpha }\,\textrm{d}z\big |^2\,\textrm{d}x\nonumber \\&\qquad \qquad \quad +\int ^b_{b-\frac{1}{2}}\rho ^{\varepsilon }_0(x)\big |S(4x-4b+2)\int ^b_x(\rho ^{\varepsilon }_0(z))^{\gamma -\alpha }\,\textrm{d}z\big |^2 \,\textrm{d}x\Big )\nonumber \\&\leqq \frac{C}{\varepsilon ^2}\Big (\int ^b_{b-\frac{1}{2}}b^{-\frac{1}{^{\gamma }}}\big |\int ^b_xb^{-\frac{\gamma -\alpha }{\gamma }}\,\textrm{d}z\big |^2\,\textrm{d}x+\int ^{-b+\frac{1}{2}}_{-b}b^{-\frac{1}{^{\gamma }}}\big |\int ^{x}_{-b}b^{-\frac{\gamma -\alpha }{\gamma }}\,\textrm{d}z\big |^2\,\textrm{d}x\Big )\nonumber \\&\leqq \frac{C}{\varepsilon ^2}b^{-\frac{2(\gamma -\alpha )+1}{\gamma }}\leqq C\varepsilon ^{{\frac{2(\gamma -\alpha )+1}{\gamma }p}-2}\rightarrow 0 \qquad \text { as } \varepsilon \rightarrow 0^+, \end{aligned}$$

for $p>\frac{\gamma }{\gamma -\alpha },$ where we have used (A23) and $b=\varepsilon ^{-p}.$

Therefore, similarly to those as in [13, 29], using Lemma A.4 and (A25), we conclude

#### Lemma A.5

For fixed $$\varepsilon >0$$,

$$\begin{aligned}&\lim _{\varepsilon \rightarrow 0^+}\int ^b_{-b}\big |\sqrt{\rho _{0}^{\varepsilon }(x)}u_{0}^{\varepsilon }(x)\big |^2\,\textrm{d}x =\int _{\mathbb {R}} \big |\frac{m_0(x)}{\sqrt{\rho _0(x)}}\big |^2\,\textrm{d}x,\\&\lim _{\varepsilon \rightarrow 0^+}\int ^b_{-b}|\rho ^{\varepsilon }_0(x)u^{\varepsilon }_0(x)-m_0(x)|\,\textrm{d}x=0. \end{aligned}$$

In the previous argument, we have already defined the cut-off for the density. Now, with $\rho ^{\varepsilon }_0(x)$ and $u_0^{\varepsilon }(x)$ defined respectively in (A21) and (A24), we also need to define the cut-off for the velocity function. For $b\gg 1$, define

A26 $$\begin{aligned} \big (\rho ^{\varepsilon }_0, u^{\varepsilon }_0\big )(x):=\big (\rho _{0}^{\varepsilon }(x), u^{\varepsilon }_0(x)\big ) \textbf{I}_{[-b,b]}(x). \end{aligned}$$

Then, collecting all the above estimates, we have the following results:

#### Lemma A.6

Let $\frac{2}{3}<\alpha \leqq 1,$ $b=\varepsilon ^{-p},$ $p>\frac{\gamma }{\gamma -\alpha }$, $q\in \{1,\gamma \}$, and $\varepsilon \in (0,1].$ Let $\big (\rho ^{\varepsilon }_0, u^{\varepsilon }_0\big )(x)$ be the functions defined in (A26) so that $(\rho ^{\varepsilon }_0, u^{\varepsilon }_0)\in C^\infty ([-b,b]).$ Then

(i) For all $$\varepsilon \in (0,1]$$,
  $$\begin{aligned}&\,\, \, b^{\frac{1}{\gamma }}\rho ^{\varepsilon }_0(-b)=b^{\frac{1}{\gamma }}\rho ^{\varepsilon }_0(b)\leqq C_0,\quad u^{\varepsilon }_0(\pm b)=0,\nonumber \\  &\quad \big (P(\rho ^{\varepsilon }_0(x))-\varepsilon \mu (\rho ^{\varepsilon }_0(x))(u^{\varepsilon }_0(x))_x\big )\big |_{x=\pm b}=0,\\  &\,\,\,\int ^b_{-b}\rho ^{\varepsilon }_0(x)\,\text {d}x=M, \qquad \mathcal {E}^{\varepsilon }_0+\varepsilon ^{-1}\mathcal {E}^{\varepsilon }_1\le C_0, \end{aligned}$$
  where $C_0>0$ is a constant independent of $\varepsilon \in (0,1]$ but may depend on $(\mathcal {E}_0, M, M_2, \gamma , \alpha )$.
(ii) As $$\varepsilon \rightarrow 0^+$$,
  $$\begin{aligned}&(\mathcal {E}_0^{\varepsilon }, \mathcal {E}_1^{\varepsilon })\rightarrow (\mathcal {E}_0, 0), \\&\int ^{\infty }_{-\infty }x^2\rho ^{\varepsilon }_0(x)\,\textrm{d}x\rightarrow \int ^{\infty }_{-\infty }x^2\rho _0 (x) \,\textrm{d}x=M_2, \\&(\rho _0^{\varepsilon }, m_0^{\varepsilon })\rightarrow (\rho _0, m_0)(x) \qquad \, \text{ in } L^q_\textrm{loc}(\mathbb {R})\times L^1_\textrm{loc}(\mathbb {R}), \end{aligned}$$
  where $\mathcal {E}_0^{\varepsilon }$ and $\mathcal {E}_1^{\varepsilon }$ are defined in (2.14) and (2.15), respectively.
